# Dissecting Solvent Effects on Hydrogen Bonding

**DOI:** 10.1002/anie.202206604

**Published:** 2022-06-14

**Authors:** Nicole Y. Meredith, Stefan Borsley, Ivan V. Smolyar, Gary S. Nichol, Christopher M. Baker, Kenneth B. Ling, Scott L. Cockroft

**Affiliations:** ^1^ EaStCHEM School of Chemistry University of Edinburgh Joseph Black Building, David Brewster Road Edinburgh EH9 3FJ UK; ^2^ Syngenta Jealott's Hill International Research Centre Bracknell, Berkshire RG42 6EY UK

**Keywords:** Electrostatic Interactions, Hydrogen Bonds, Noncovalent Interactions, Solvent Effects

## Abstract

The experimental isolation of H‐bond energetics from the typically dominant influence of the solvent remains challenging. Here we use synthetic molecular balances to quantify amine/amide H‐bonds in competitive solvents. Over 200 conformational free energy differences were determined using 24 H‐bonding balances in 9 solvents spanning a wide polarity range. The correlations between experimental interaction energies and gas‐phase computed energies exhibited wild solvent‐dependent variation. However, excellent correlations were found between the same computed energies and the experimental data following empirical dissection of solvent effects using Hunter's α/β solvation model. In addition to facilitating the direct comparison of experimental and computational data, changes in the fitted donor and acceptor constants reveal the energetics of secondary local interactions such as competing H‐bonds.

## Graphical abstract

The perturbing effects of solvation obscure the energetics of H‐bonding interactions. Experimental H‐bond energies were determined in a wide range of competitive solvents using synthetic molecular balances. Dissection of the H‐bond and solvation energetics using Hunter's solvation model enabled the direct comparison of experimental and gas‐phase computational energies and the rationalization of competing secondary electrostatic interactions.

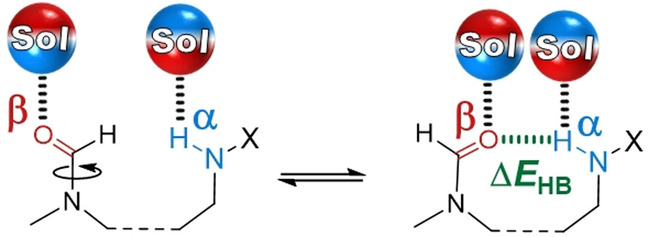

Hydrogen bonding is central to biology and countless synthetic systems.^[1]^ The nature of H‐bonds has been the subject of historic debate.[^2^, ^3^] Although purely electrostatic descriptions do not fully account for the characteristics of H‐bonds, the electrostatic contributions are often major.^[4]^ Accordingly, H‐bonds are particularly sensitive to solvent effects[^5^, ^6^, ^7^, ^8^] and competing interactions.[^9^, ^10^, ^11^] While biology has mastered the use of H‐bonding in a highly competitive solvent,^[12]^ synthetic chemists have yet to develop comparable levels of control. Several synthetic systems have repurposed biological H‐bonding motifs, but controlling and predicting the behavior of de novo H‐bonding systems in competitive solvents is more difficult. This situation has been highlighted in medicinal chemistry,[^13^, ^14^, ^15^, ^16^] the construction of H‐bonding chains,[^17^, ^18^, ^19^, ^20^, ^21^, ^22^, ^23^] and sugar binding[^24^, ^25^, ^26^, ^27^, ^28^] in competitive solvents. Therefore, it remains desirable to quantify individual H‐bonds in competitive solvents to better understand their behavior.

At a basic level, H‐bonds are stronger in apolar solvents and weaker in polar solvents. Hunter has quantitatively related empirical parameters for the H‐bond donor (α) and acceptor (β) abilities of functional groups to the free energy of H‐bonds in solution.^[31]^ The role of solvent is modelled by considering the competing H‐bond donor (α_s_) and acceptor (β_s_) abilities of the solvent, and has been shown to account for the energetics of a very strong H‐bond donor/acceptor pair in a range of solvents and solvent mixtures.[^5^, ^32^] However, the assessment of weaker H‐bonds, especially those occurring in competitive media has tended to rely on perturbations in host–guest complexation, which makes it difficult to deconvolute secondary influences such as limited solvent accessibility within inclusion complexes.[^24^, ^29^, ^30^]

Molecular balances present an alternative to supramolecular complexation for the study of molecular recognition.[^33^, ^34^, ^35^, ^36^, ^37^, ^38^] Molecular balances exploit conformational changes that report the strength of intramolecular interactions that are present in one conformation but absent in another (e.g. Figure 1). The approach offers several advantages over intermolecular complex formation between two (or more) species.^[33]^ Molecular balances generally provide a high‐level of control over interaction geometries. The entropic cost of bringing molecules together is also negated, which means that interactions that are repulsive, weak, or strongly susceptible to solvent disruption can still be measured. Moreover, since the position of a conformational equilibrium can often be determined with high accuracy from a single NMR spectrum, the measurement of very weak interactions at low mM concentrations is facilitated across a wide range of solvents. Accordingly, molecular balances have been used to evaluate diverse interactions[^39^, ^40^, ^41^, ^42^, ^43^, ^44^, ^45^, ^46^, ^47^, ^48^, ^49^, ^50^, ^51^, ^52^, ^53^, ^54^] and solvent effects.[^35^, ^37^, ^38^, ^55^, ^56^, ^57^, ^58^, ^59^] To date, molecular balance studies of H‐bonding in amides[^60^, ^61^] or alcohols^[62]^ have been confined to non‐competitive apolar solvents. Meanwhile, systematic examinations of solvent effects have been limited to molecular balances lacking H‐bonding motifs.[^31^, ^35^, ^39^]

**Figure 1:**
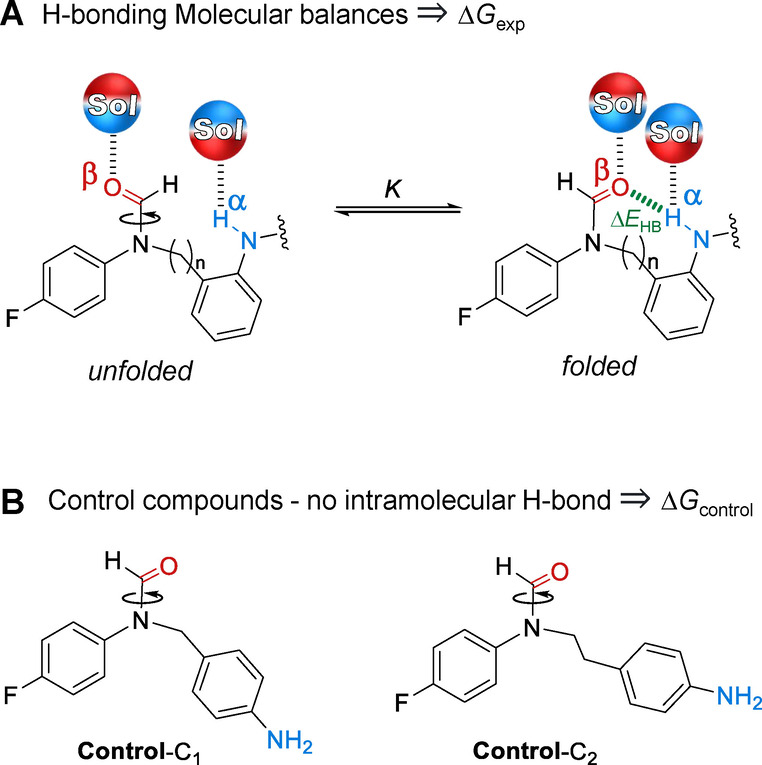
A) Molecular balance design employed in the present investigation to measure H‐bonding interactions in solution. All compound variants are depicted in Figure 2 and included a variable C_*n*_ linker (C_1_=CH_2_, or C_2_=CH_2_CH_2_). B) Control compounds quantify steric and other secondary contributions to the position of the conformational equilibrium. Application of Hunter's solvation model^[31]^ enabled further dissection of the solvent‐independent H‐bond energy (Δ*E*_HB_) and the difference between the H‐bond donor (α) and acceptor (β) abilities of the molecular balance in the folded and unfolded conformations (see Section S3.4, Supporting Information).

Here we have used 24 synthetic molecular balances (Figures 1 and 2) to investigate competitive solvation of amine and amide H‐bonds. The geometries of the intramolecular H‐bonds were examined computationally and in the solid state. The conformational free energies of the molecular balances were measured in 9 solvents spanning a large polarity range (Figure 2). Application of Hunter's H‐bond solvation model^[31]^ enabled the empirical dissection of the intramolecular H‐bond energies from the modulating influence of the solvent effects (Table 1). Comparison of the computationally determined interaction energies and those dissected from the experimental data enabled an assessment of the utility of the Hunter solvation model for both revealing and rationalizing H‐bond energetics in a wide range of solvents (Figure 3).

**Figure 2:**
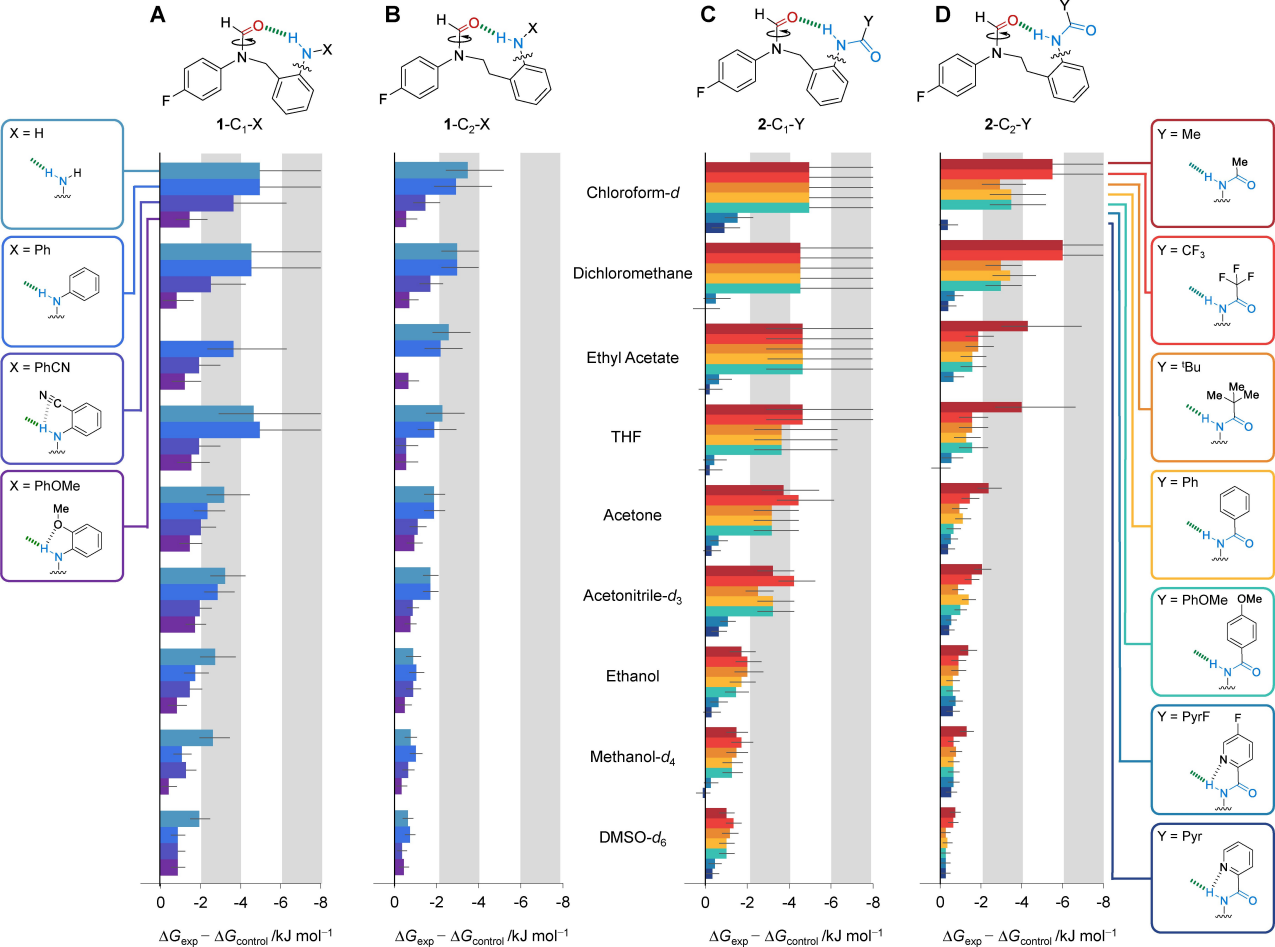
Experimental conformational free energies (Δ*G*_exp_) were measured in nine different solvents by ^19^F{^1^H} NMR spectroscopy (376.5 MHz, 298 K) for H‐bonding balance series A) **1**‐C_1_‐X, B) **1**‐C_2_‐X, C) **2**‐C_1_‐Y, D) **2**‐C_2_‐Y. Steric and secondary contributions to the conformational free energy differences were accounted for by subtracting the conformational free energies of the respective methylene (**Control**‐C_1_) or ethylene‐linker (**Control**‐C_2_) balances measured in each solvent (Δ*G*_control_, Figure 1B) from the Δ*G*_exp_ values of the H‐bonding balances. Negative Δ*G*_exp_ values are defined as a preference for the folded (H‐bonded) conformation. All data and errors are tabulated in Section S3.1, Supporting Information.

**Figure 3:**
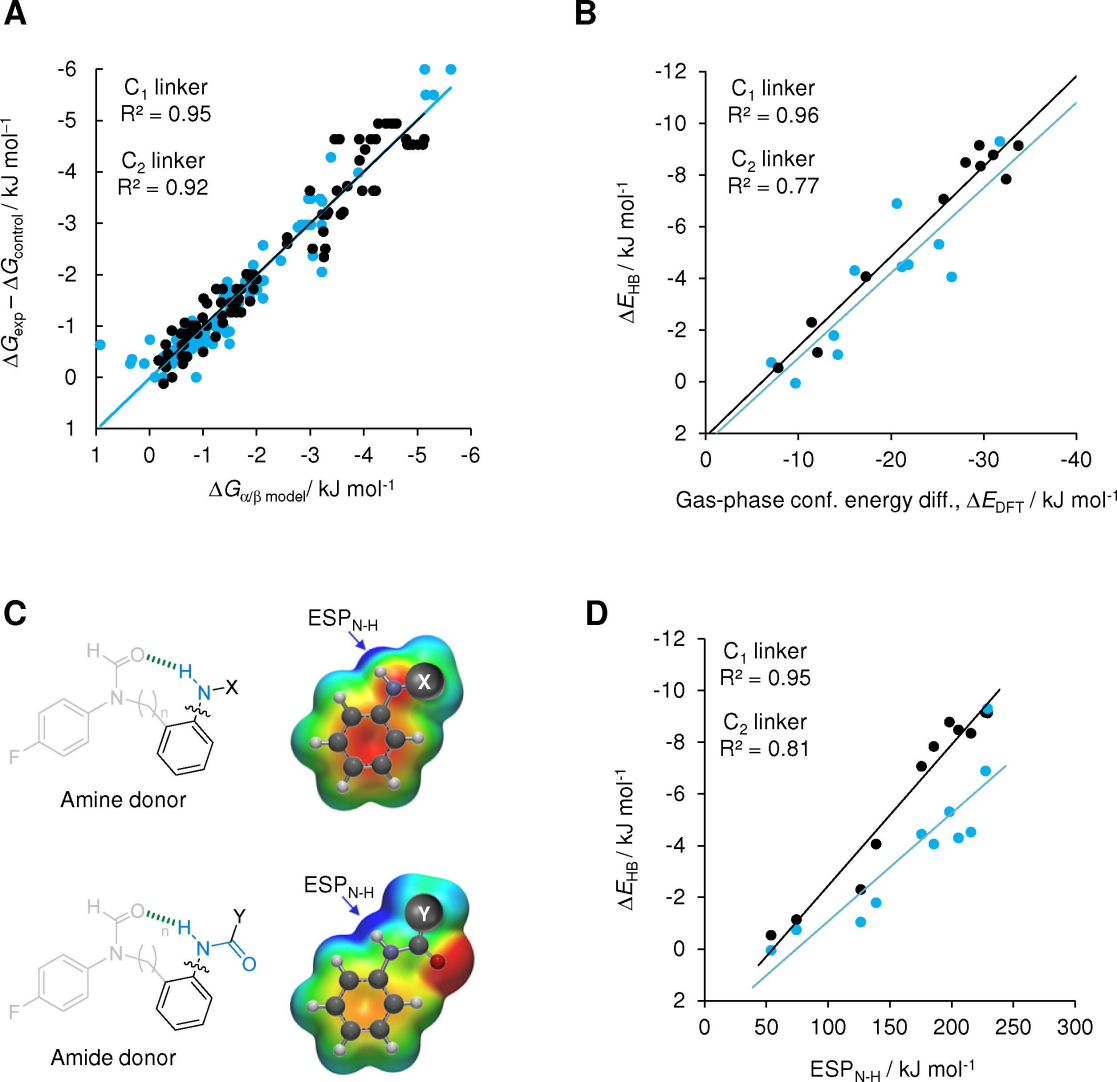
A) Experimental measurements of the intramolecular H‐bonding interactions determined in nine solvents using balance series **1**‐n‐X and **2**‐n‐Y (approximated by Δ*G*_exp_−Δ*G*_control_) plotted against those determined by fitting against the Hunter solvation model Δ*G*_α/β model_=Δα β_s_+Δβ α_s_+Δ*E*_HB_, where α_s_ and β_s_ are the known H‐bond donor and acceptor constants of the solvent, Δα and Δβ are the changes in the H‐bond donor and acceptor constants of the balance upon forming the intramolecular H‐bond, and Δ*E*_HB_ is the solvent‐independent energy of the intramolecular H‐bond. B) Correlations of the empirically dissected solvent‐independent H‐bond energies, Δ*E*_HB_ with the gas‐phase energy difference between the folded and unfolded conformers calculated via a conformer distribution search (DFT/B3LYP/6‐31G*). C) Calculated electrostatic surface potentials (DFT/B3LYP/6‐31G*) at the 0.002 electron Å^−3^ isosurface along the N−H bond axis correlate strongly with D) the empirically dissected solvent‐independent H‐bond energies, Δ*E*_HB_ determined by fitting with the Hunter solvation model. All data and errors are tabulated in Tables S10–S32, Supporting Information.

Molecular balance series **1**‐C_*n*_‐X and **2**‐C_*n*_‐Y were designed and synthesized to study H‐bonds with amine and amide donors, respectively (Figures 1 and 2, see Section S2, Supporting Information). Both series included a variable C_*n*_ linker (C_1_=CH_2_, or C_2_=CH_2_CH_2_) between the H‐bond donor and acceptor sites. Minimized structures of the folded and unfolded conformations of these balances were determined using DFT calculations (see Section S4, Supporting Information). All balances formed the desired intramolecular H‐bond in the folded conformation indicating the suitability of the designs (O to H distances of <2.4 Å). An X‐ray crystal structure of balance **2**‐C_1_‐Me in the folded conformation was obtained by slow evaporation from CH_2_Cl_2_ (see Section S2.4, Supporting Information, CSD deposition no. 2157270). The O to H distance of the intramolecular H‐bond in the crystal structure compared favorably with the calculated structure (1.893 Å vs. 1.914 Å DFT/B3LYP/6‐31G*, Figure S51, Supporting Information).

The minimal design of the balances simplifies the analysis of the experimentally determined conformational preferences. Rotation around the formamide C−N bond is slow on the NMR timescale, which provides discrete peaks corresponding to the folded and unfolded conformers. Conformers were initially assigned using HMBC/NOESY NMR spectroscopy (see Section S2.3, Supporting Information). The assignment of conformers was greatly simplified by the different steric demands on each side of the balance (phenyl group vs. methyl/ethyl benzene), which favored the folded conformation in all solvents examined. Nine common laboratory solvents were selected that spanned a range of H‐bond donor and acceptor constants (α_s_ and β_s_, respectively, Table S35). Integration of the conformer peaks observed in ^19^F NMR spectra provided access to the conformational equilibrium constant, *K*, and therefore the conformational free energy difference from Δ*G*_exp_=−*R* *T* ln*K* (see Section S3, Supporting Information). Since the preference for the folded conformation was not entirely governed by the intramolecular H‐bond, control balances that lacked the ability to form intramolecular H‐bonds with both methylene and ethylene linkers were also synthesized (**Control**‐C_1_ and **Control**‐C_2_, Figure 1B). The conformational free energy of each H‐bonding balance (Δ*G*_exp_) was then corrected for steric effects (and any other secondary interactions) by subtracting the conformational free energy differences of the respective control (Δ*G*_control_) in each solvent. The Δ*G*_exp_−Δ*G*_control_ energy differences in Figure 2 therefore approximate the energies of the intramolecular H‐bonds for each balance/solvent combination.

The intramolecular H‐bond energies approximated by Δ*G*_exp_−Δ*G*_control_ were most favored in apolar solvents and least favored in polar solvents (0 to −6 kJ mol^−1^, Figure 2). Similarly, wild solvent‐dependent variation in the gradients was observed when Δ*G*_exp_−Δ*G*_control_ was plotted against the computed gas‐phase conformational energy differences of the balances (Δ*E*_DFT_, B3LYP/6‐31G*) (R^2^=0.35–0.94, Figure S53). Hence, we sought to examine whether Hunter's empirical solvation model could be used to dissect out the solvent effects to gain further insight into the data.[^5^, ^31^, ^32^] Indeed, Hunter's model has previously been used to rationalize solvent competition in the conformational equilibria of molecular balances lacking H‐bonding motifs.[^31^, ^54^] Dissection of solvent effects in the present work was performed by performing a linear regression of the H‐bond energies approximated by Δ*G*_exp_−Δ*G*_control_ against those predicted by the model (Δ*G*_α/β model_) as the H‐bond donor and acceptor constants of the solvent were varied (see Section S3.4, Supporting Information). Fitting the data in this manner gave excellent agreement between the experimental and modelled free energies for both the methylene and ethylene‐linked series (R^2^=0.95 and 0.92, Figure 3A). The fitting revealed the dissected solvent‐independent H‐bond energies (Δ*E*_HB_) and the terms encoding the change in the H‐bond donor/acceptor constants (Δα and Δβ) of the balance upon forming the intramolecular H‐bond (Table 1).

Good correlations were observed between the empirically dissected solvent‐independent H‐bond energies, Δ*E*_HB_ and the calculated gas‐phase energy difference between the folded and unfolded conformers (DFT/B3LYP/6‐31G*, R^2^=0.96 and 0.77 for the methylene and ethylene linker series respectively, Figure 3B). These correlations contrast with the aforementioned wild solvent‐dependent variation in the gradients seen when the same computed energies were plotted against the experimental Δ*G*_exp_−Δ*G*_control_ values (R^2^=0.34–0.94, Figure S53). Hence, the improved correlations are consistent with solvent effects being dissected out.

The dissected Δ*E*_HB_, Δα, and Δβ values provide numerous insights into the nature of the intramolecular H‐bonds in the molecular balances (Table 1). The most favorable Δ*E*_HB_ energies were associated with the largest changes in H‐bond donor/acceptor constants (Δα and Δβ) of the balance upon forming the intramolecular H‐bond. This is consistent with stronger H‐bonds being most strongly perturbed by the competing effects of the solvent. The dissected Δβ values for each balance are 2–3 times larger than the corresponding Δα values. This is commensurate with the relative values of the α/β donor/acceptor constants;^[31]^ the formyl oxygen acceptor in the balances has β≈8.3 relative α=2.0 to 2.9 for the aniline/amide NH donors. All of the dissected Δα and Δβ values are smaller than these maximal α and β values of the H‐bond donor and acceptor sites, which suggests that solvation of these sites is not fully switched on or off in the unfolded or folded conformations. Correspondingly, the Δα values for the more flexible ethyl‐linked balances are proportionally larger (in relation to the intramolecular H‐bond Δ*E*_HB_ energies) than the methylene‐linker series. Similarly, the dissected intramolecular Δ*E*_HB_ H‐bond energies of the ethylene‐linker balances are ≈1 to 3.1 kJ mol^−1^ less favorable than the methylene‐linked variants containing the same H‐bond donor groups. Additionally, the correlations in Figure 3 for the ethylene‐linker balances (blue) are more scattered than the methylene‐linker variants (black). Similar scattering persisted when Δ*G*_exp_ values were plotted that were not corrected with Δ*G*_control_. These observations are consistent with the increased flexibility of the ethylene linker and the estimated cost of restricting the rotation of a C_sp_^3^−C_sp_^3^ bond of between 1 and 7 kJ mol^−1^ at 298 K,^[64]^ based on the properties of alkanes,^[65]^ ring‐closing reactions,[^66^, ^67^, ^68^, ^69^, ^70^] and molecular recognition processes.[^71^, ^72^, ^73^, ^74^, ^75^, ^76^, ^77^, ^78^, ^80^]

**Table 1:**
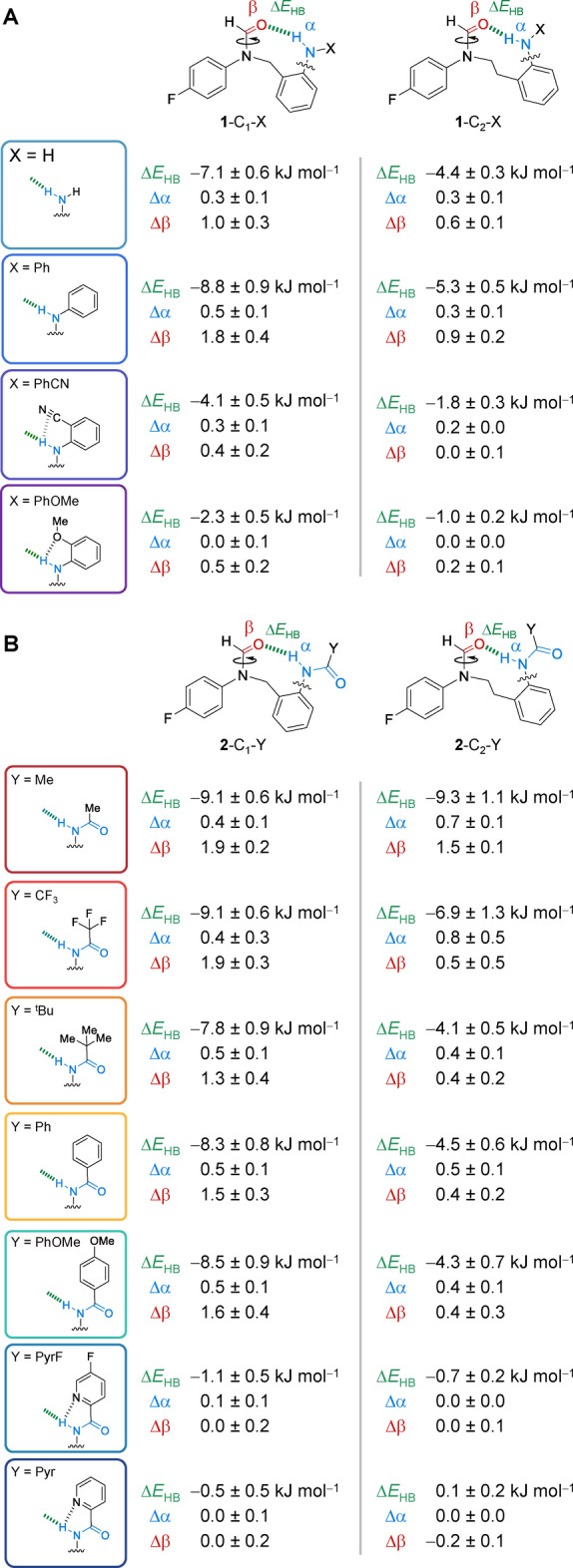
Dissected solvent‐independent H‐bond energy (Δ*E*_HB_), and the change in H‐bond donor (Δα) and acceptor (Δβ) abilities of the molecular balances determined by fitting experimental conformational free energies to the Hunter solvation model Δ*G*α/β model=Δα β_s_+Δβ α_s_+Δ*E*_HB_ (see Section S3.4, Supporting Information).[^5^, ^31^, ^32^]

Most importantly, the dissected Δ*E*_HB_, Δα, and Δβ values reveal the nature of the local chemical environments of the intramolecular H‐bonds. Balances bearing *ortho*‐positioned substituents or pyridine nitrogen atoms on the X/Y ring (**1**‐n‐OMe, **2**‐n‐Pyr, **2**‐n‐PyrF, **1**‐n‐PhCN) had substantially diminished Δ*E*_HB_ H‐bond energies. Similarly, the Δα and Δβ values for these compounds were very close to zero, indicating that solvation of the H‐bond donors and acceptors changed little between the folded and unfolded conformations. These observations can be rationalized as arising from competing H‐bond interactions involving the *ortho*‐functional groups (depicted in the structures in Table 1). However, such secondary interactions might be more accurately described as additive through‐space electrostatic interactions.^[63]^ Supporting this hypothesis, calculated electrostatic potentials taken along the N−H bond axis at the 0.002 electron/Å^3^ isosurface (ESP_N−H_) for the H‐bond donor aniline / phenyl amide fragments shown in Figure 3C (B3LYP/6‐31G*) correlated strongly with the empirically dissected intramolecular Δ*E*_exp_ values (R^2^=0.95 and 0.81, Figure 3D). In contrast, the gradients of the correlations between the same N−H electrostatic potentials and the same experimental Δ*G*_exp_−Δ*G*_control_ energy differences once again exhibited wild solvent‐dependent variation (Figure S54, R^2^=0.28 to 0.92). The ability to relate local structural information combined with the strong correlations seen in Figure 3 demonstrate the excellent utility of the α/β solvation model in dissecting and rationalizing solvent effects.

In summary, we have performed a combined experimental and theoretical investigation of amide and amine H‐bonding interactions in solution using molecular balances. Application of Hunter's solvation model[^5^, ^31^, ^32^] allowed solvent effects to be dissected. The approach distils disparate behavior observed in different solvents to enable the direct comparison of dissected experimental energies with computational equivalents. The dissected changes in the H‐bond donor and acceptor constants Δα and Δβ encode the changes in the solvation of the solute upon forming H‐bonding interactions. The study demonstrates the utility of Hunter's solvation model for deconvoluting and rationalizing the behavior of molecular systems, in particular the ability to reveal the associated secondary interactions and solvation changes in the local chemical environment. The validity of the approach across a range of highly competitive polar solvents augers well for understanding biologically relevant interactions in competitive solvents, such as the study of H‐bond chains^[62]^ and arrays.^[79]^ We hope that the approach will be applied more broadly to leverage the computational understanding and analysis of solution‐phase experimental behaviour.

## Conflict of interest

The authors declare no conflict of interest.

## Supporting information

As a service to our authors and readers, this journal provides supporting information supplied by the authors. Such materials are peer reviewed and may be re‐organized for online delivery, but are not copy‐edited or typeset. Technical support issues arising from supporting information (other than missing files) should be addressed to the authors.

Supporting Information

Supporting Information

## Data Availability

The data that support the findings of this study are available in the Supporting Information of this article.
