# Supplementary material for: Dissecting Solvent Effects on Hydrogen Bonding
Source: Angew Chem Int Ed Engl. 2022 Jun 14;61(30):e202206604. doi: 10.1002/anie.202206604 (PMC9400978; doi:10.1002/anie.202206604)
Supplement: Supplementary file 1 — Supporting Information [file ANIE-61-0-s002.pdf]

Angewandte Chemie
Eine Zeitschrift der Gesellschaft Deutscher Chemiker

Supporting Information

Dissecting Solvent Effects on Hydrogen Bonding

*N. Y. Meredith, S. Borsley, I. V. Smolyar, G. S. Nichol, C. M. Baker, K. B. Ling, S. L. Cockroft**

Contents

S1. General experimental procedures 3
S2. Molecular torsion balances investigated 4
S2.1 Full structures of all balances considered 4
S2.2 Synthetic procedures and standard characterisation data 5
S2.2.1 Methylene (C_1) linker series 5
S2.2.2 Ethylene (C_2) linker series 35
S2.3 Conformer assignment by NMR spectroscopy 66
S2.4 Crystal Structure of **2**-C_1-Me 70
S3. Experimental conformational free energies, ΔG_{exp} and $\Delta G_{control}$ 78
S3.1 NMR determination of conformational free energies, ΔG_{exp} and $\Delta G_{control}$ 78
S3.3 Van't Hoff analyses of compounds **2**-C_1-Ph and **2**-C_2-Ph 93
S3.4 Dissection of interaction energies using Hunter's solvation model 95
S4. Computational methods and data 103
S4.1 Geometry minimisation and calculated conformational free energies 103
S4.2 Barrier to rotation calculation 110
S3.2 Experimental energies vs. calculated properties 112
S5. References 114

S1. General experimental procedures

Unless stated otherwise, all chemicals were purchased from commercial sources (Sigma Aldrich UK, Acros UK, VWR UK or Fluorochem UK) and used without further purification. Dry solvents were obtained by means of a "Glass Contour" brand solvent purification system, where solvents were passed through filter columns and dispensed under an argon atmosphere. Flash column chromatography was performed using Geduran® Si60 (40-63 mm, Merck, Germany) as the stationary phase, and thin-layer chromatography (TLC) was performed on pre-coated silica gel plates (0.25 mm thick, 60F254, Merck, Germany) and observed under UV light (l_{max} 254 nm). Mass spectrometry was performed by the University of Edinburgh technician-supported mass spectrometry service, using a Bruker micrOTOF II or ThermoElectron MAT XP spectrometer for EI or ESI-HRMS. Melting points were measured in a Gallenkamp melting point apparatus, and are reported uncorrected. ^{1}H and ^{13}C NMR spectra were recorded on Bruker Ultrashield 400 MHz, Bruker Ascend 500 MHz equipped with a DCH cryoprobe and Bruker Ascend 500 MHz with prodigy cryoprobe and Bruker Ultrashield 600 MHz with TCI cryoprobe, at a constant temperature of 25 °C, unless otherwise stated. ^{1}H, ^{13}C, ^{19}F and ^{195}Pt chemical shifts are reported in parts per million (ppm) from low to high field. ^{1}H and ^{13}C values are referenced to the literature values for chemical shifts of residual non-deuterated solvent, with respect to tetramethylsilane. ^{19}F is referenced externally to $CFCl_3$ at 0 ppm and ^{195}Pt is referenced externally to K_2PtCl_6 at 0 ppm. Standard abbreviations indicating multiplicity are used as follows: bs (broad singlet), d (doublet), dd (doublet of doublets), m (multiplet), q (quartet), s (singlet), t (triplet), tt (triplet of triplets), *J* (coupling constant). All spectra were analysed using MestReNova (Version 11.0.0). NMR tubes, precision glassware and glass syringes were dried under vacuum before use. Deuterated solvents were stood over oven-dried activated 4 Å molecular sieves for a minimum of 24 h prior to use. Non-deuterated anhydrous solvents were used directly as commercially obtained anhydrous solvents, or were redistilled under reduced pressure from analytical-grade solvents.

S2. Molecular torsion balances investigated

S2.1 Full structures of all balances considered

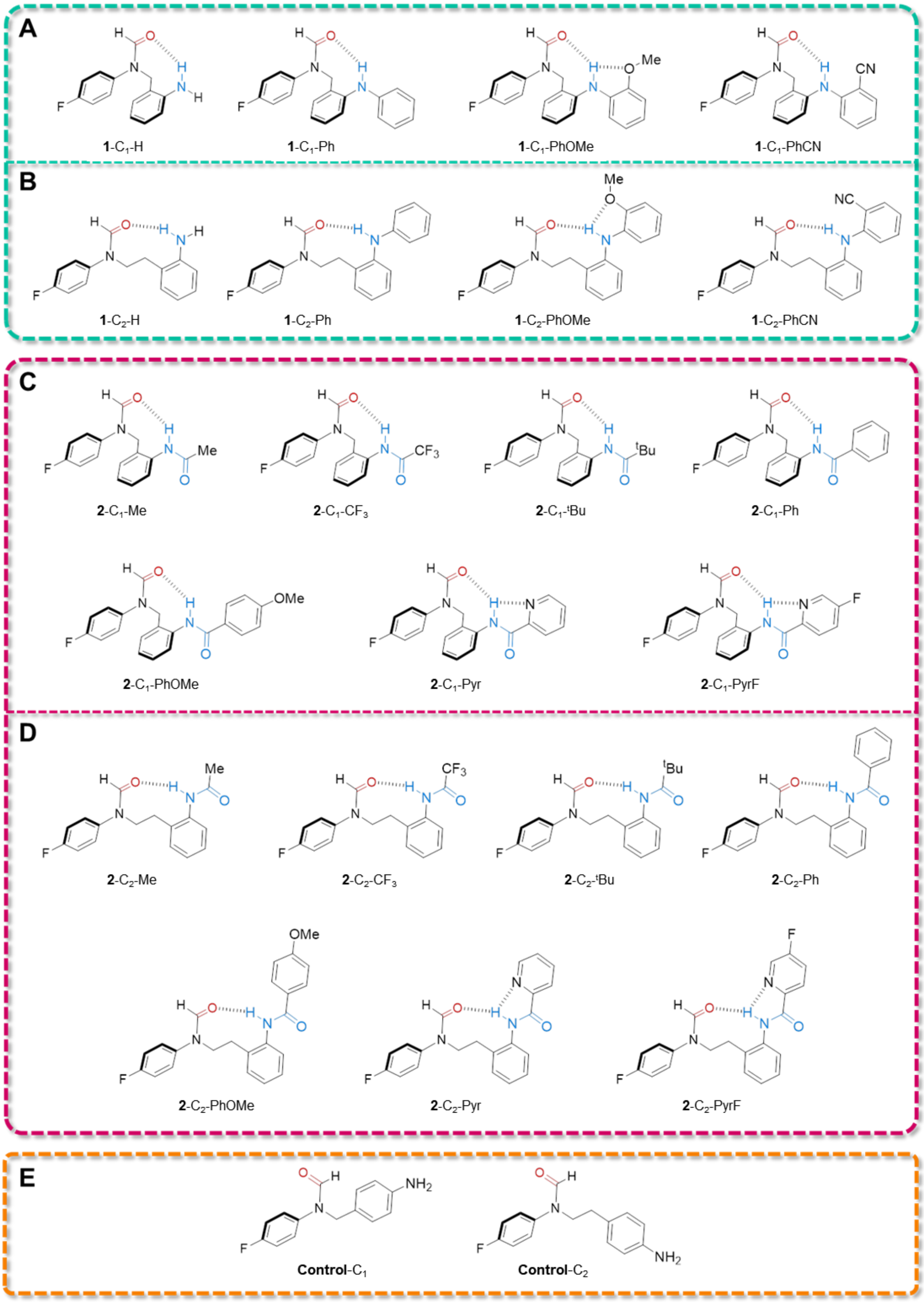

Figure S1 Full structures of all balances. (**A**) Aniline balances containing methylene (C_1) linkers. (**B**) Aniline balances containing ethylene (C_2) linkers. (**C**) Amide balances containing methylene (C_1) linkers. (**D**) Amide balances containing ethylene (C_2) linkers. (**E**) Control balances containing methylene (C_1) and ethylene (C_2) linkers that are sterically prohibited from forming intramolecular hydrogen bonds.

S2.2 Synthetic procedures and standard characterisation data

S2.2.1 Methylene (C_1) linker series

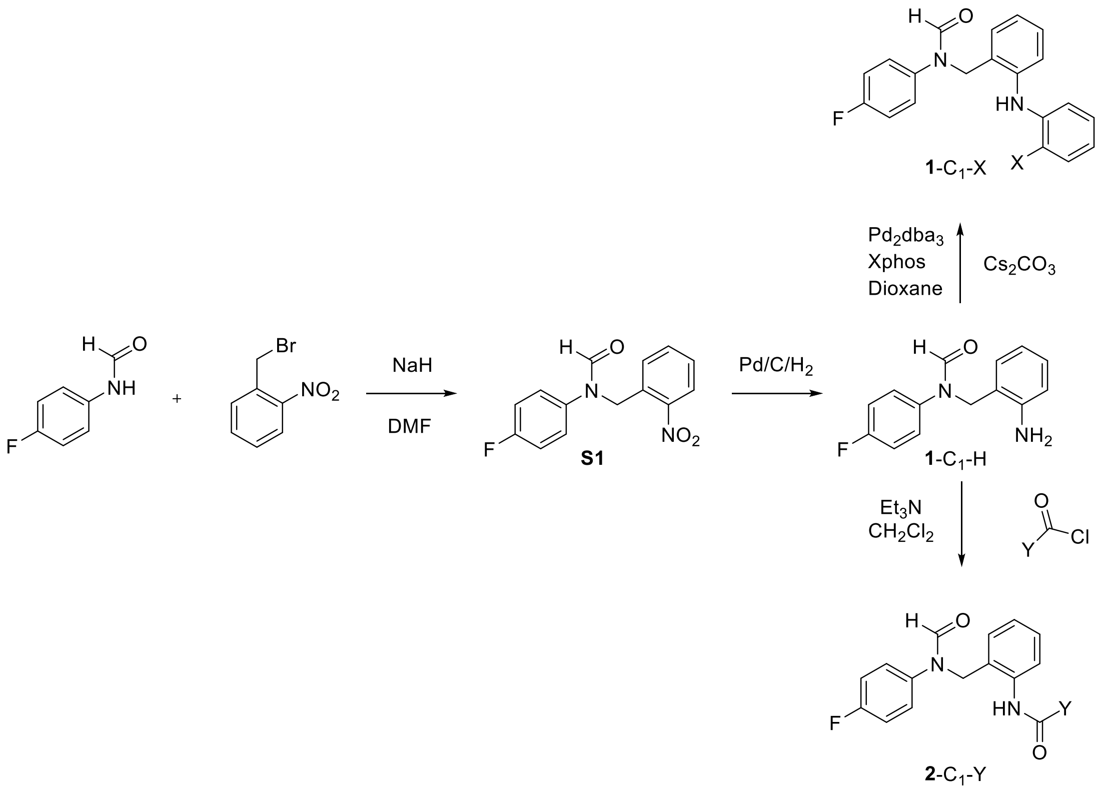

Figure S2 General scheme for the synthesis of balance series **1**-C_1-X and **2**-C_1-Y.

N-(4-fluorophenyl)-*N*-[(2-nitrophenyl)methyl]formamide **S1**

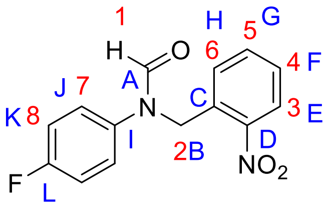

To a flask under a nitrogen atmosphere was added a solution of *N*-(4-fluorophenyl) formamide (870 mg, 6.25 mmol), in dry DMF (30 mL), 2-nitrobenzyl bromide (1.35 g, 6.25 mmol) was then added and the mixture cooled to 0 °C. Sodium hydride (300 mg, 7.50 mmol) was then carefully added and the mixture allowed to warm to room temperature. The reaction mixture was stirred overnight, diluted with CH_2Cl_2 (10 mL) and quenched with water (10 mL). The organics were then dried with $MgSO_4$, reduced *in vacuo*, and purified by flash column chromatography (SiO_2, *n*-Hex/EtOAc, 7:3, *v*/*v*) to yield a white solid (800 mg, 2.91 mmol, 50%).

^{1}H NMR (500 MHz, $CDCl_3$) δ = 8.61 (1, s, 1H), 8.52 (1’, s, 1H), 8.10 (3’, dd, *J* = 8.2, 1.4 Hz, 1H), 8.05 (3, dt, *J* = 7.6, 1.2 Hz, 1H), 7.65–7.60 (5, m, 1H), 7.53–7.49 (4’, m, 1H), 7.48–7.44 (5’, 6, 4, m, 3H), 7.41 (6’, m, 1H), 7.26–7.22 (7’, m, 2H), 7.15–7.06 (7, 8, m, 4H), 7.05–7.01 (8’, m, 2H), 5.39 (2, s, 2H), 5.29 (2’, s, 2H).

^{13}C NMR (126 MHz, $CDCl_3$) δ = 162.99 (A’, s), 162.46 (A, s), 161.41 (L, d, *J* = 247.8 Hz), 148.30 (D, s), 148.10 (D’, s), 136.75 (I, d, *J* = 3.1 Hz), 134.29 (I’, d, *J* = 3.3 Hz), 133.87 (G’, s), 133.75 (G, s), 131.97 (C’, s), 131.78 (C, s), 129.81 (H’, s), 129.22 (F’, s), 129.03 (H, s), 128.47 (F, s), 126.82 (J’, d, *J* = 8.4 Hz), 125.63 (E’, s), 125.43 (J, d, *J* = 8.5 Hz), 125.30 (E, s), 116.79 (K, d, *J* = 22.9 Hz), 116.19 (K’, d, *J* = 22.6 Hz), 51.10 (B’, s) 46.67 (B, s).

^{19}F{^{1}H} NMR (471 MHz, $CDCl_3$) δ = −114.02 (major), −114.24 (minor).

EI HRMS: obtained m/z 274.07607 M^+ (expected m/z 274.07482 M^+).

MP: 115–116 °C.

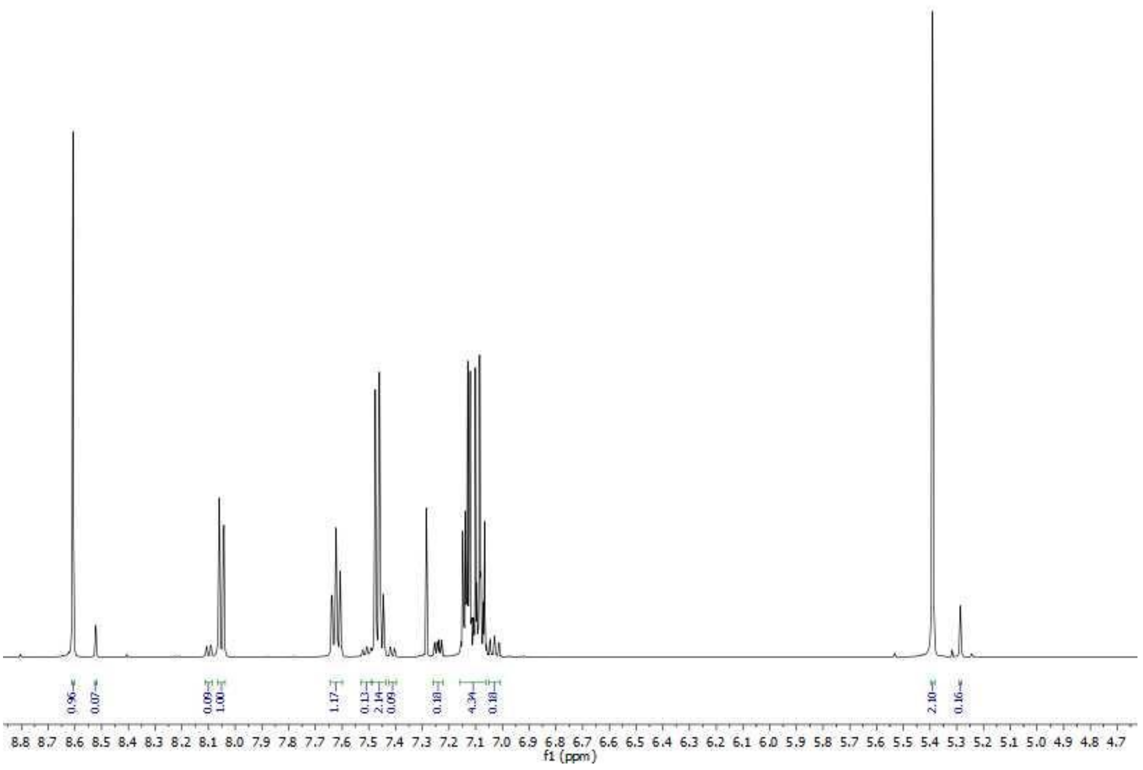

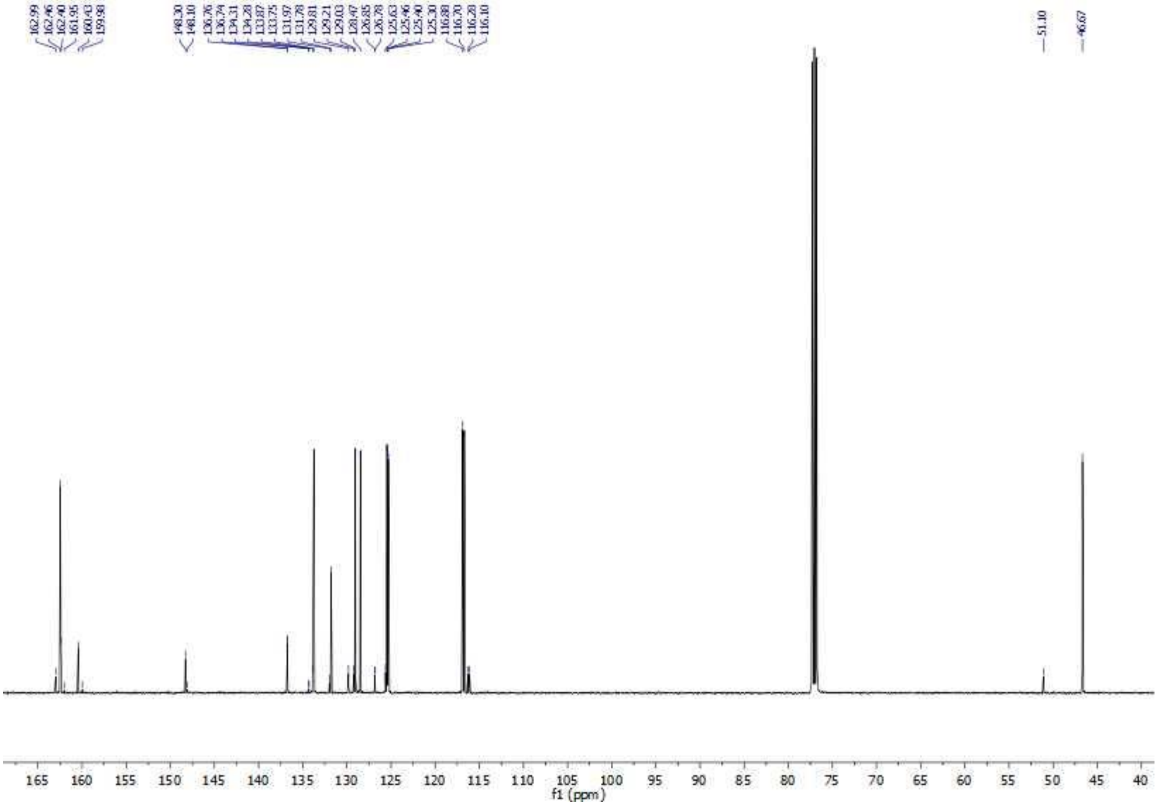

Figure S3 (Top) Partial ^{1}H NMR spectrum (500 MHz, 298 K, $CDCl_3$) and (Bottom) partial ^{13}C NMR spectrum (126 MHz, 298 K, $CDCl_3$) of compound **S1**.

N-[2-(2-aminophenyl)ethyl]-*N*-(4-fluorophenyl)formamide **1**-C_1-H

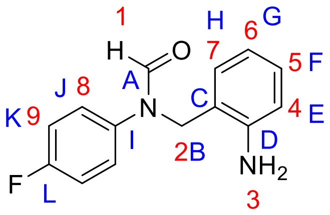

To a reaction flask under a nitrogen atmosphere was added a solution of *N*-(4-fluorophenyl)-*N*-[(2-nitrophenyl)methyl]formamide (200 mg, 0.730 mmol) in THF (10 mL) and EtOH (10 mL). Palladium on carbon (10 *wt*%) was then added and the reaction mixture placed under a hydrogen environment. The reaction mixture was monitored to completion via TLC and then filtered through Celite. The crude mixture was reduced under pressure and purified by flash column chromatography (SiO_2, CH_2Cl_2/MeOH, 49:1, *v*/*v*) to yield a white solid (140 mg, 79%).

^{1}H NMR (601 MHz, DMSO-d_6) δ = 8.54 (1', s, 1H), 8.51 (1, s, 1H), 7.35-7.31 (8, m, 2H), 7.30-7.28 (8', m, 2H) 7.24–7.19 (9, m, 2H), 7.17–7.13 (9', m, 2H), 6.96–6.95 (6', m, 1H), 6.94–6.90 (6, m, 1H), 6.81–6.80 (7', m, 1H), 6.79–6.76 (7, m, 1H), 6.64–6.65 (4', m, 1H), 6.63–6.61 (4, m, 1H), 6.47–6.44 (5', m, 1H), 6.43–6.39 (5, m, 1H), 5.05 (3, s, 2H), 5.03 (3', s, 2H), 4.82 (2, s, 2H), 4.79 (2', s, 2H).

^{13}C NMR (151 MHz, DMSO-d_6) δ = 163.66 (A', s), 163.14 (A, s), 160.75 (L, d, *J* = 243.2 Hz), 146.68 (D', s), 146.60 (D, s), 137.33 (**I**, d, *J* = 2.7 Hz), 135.77 (I', d, *J* = 3.0 Hz), 129.46 (H, s), 128.93 (H', s), 128.66 (G', s), 128.60 (G, s), 127.70 (J', d, *J* = 8.5 Hz), 126.24 (J, d, *J* = 8.5 Hz), 116.52 (K, d, *J* = 22.7 Hz), 116.65 (F', s), 116.46 (K', d, *J* = 22.7 Hz), 116.28 (F, s), 115.81 (E', s), 115.24 (E, s), 120.08 (C', s), 119.10 (C, s) 49.49 (B', s), 44.67 (B, s).

^{19}F{^{1}H} NMR (471 MHz, DMSO-d_6) δ = −116.18 (major + minor overlapping).

EI HRMS: obtained m/z 244.10012 M^+ (expected m/z 244.10064 M^+).

MP: 72–74 °C.

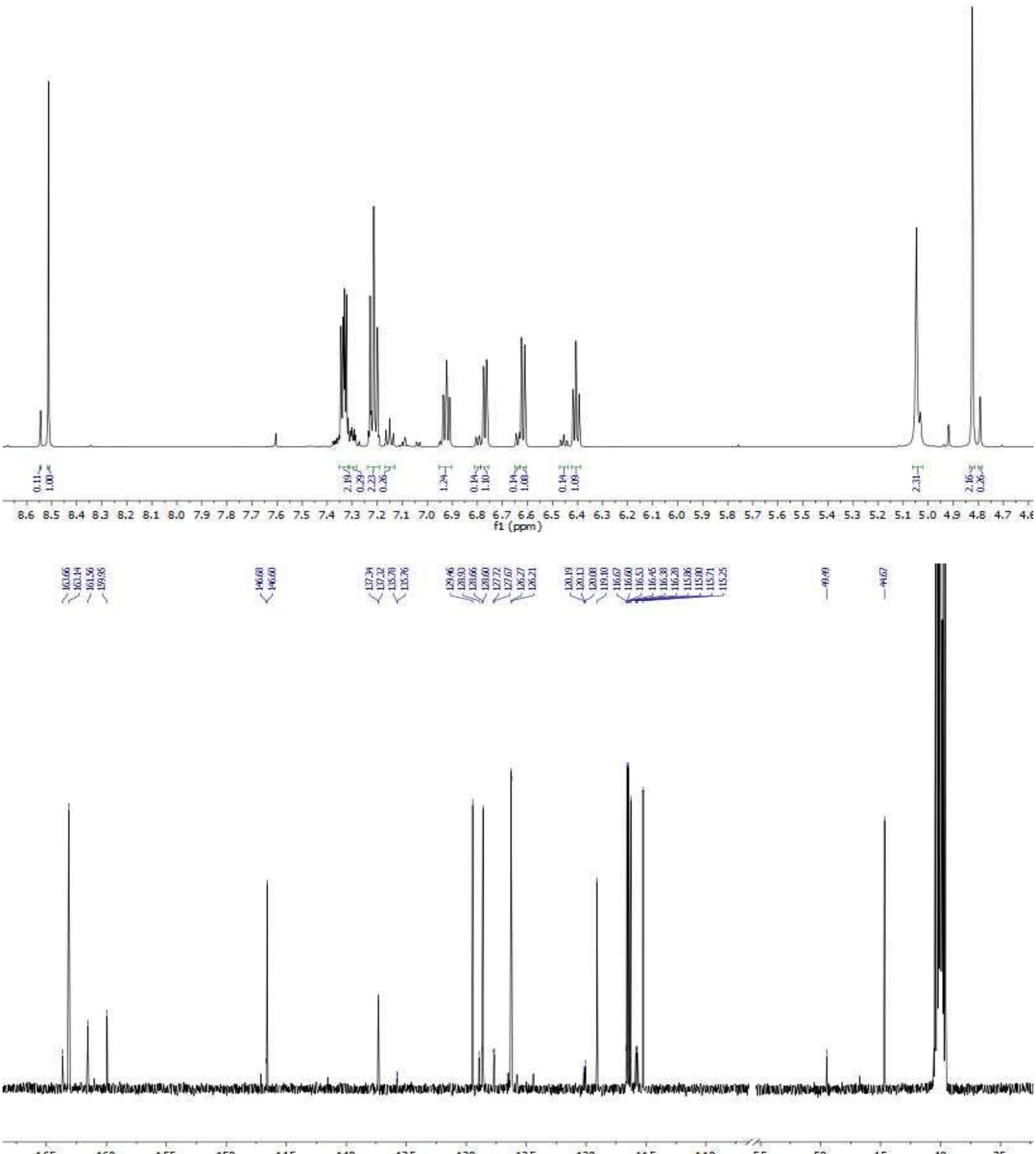

Figure S4 (Top) Partial ^{1}H NMR spectrum (500 MHz, 298 K, DMSO-d_6) and (Bottom) partial ^{13}C NMR spectrum (126 MHz, 298 K, DMSO-d_6) of compound **1**-C_1-H.

N-(4-fluorophenyl)-*N*-[(4-nitrophenyl)methyl]formamide **S2**

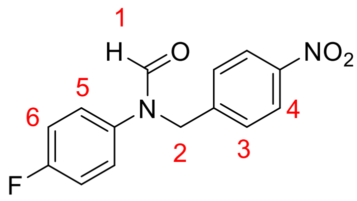

To a flask under a nitrogen atmosphere was added a solution of *N*-(4-fluorophenyl) formamide (400 mg, 2.88 mmol), in dry DMF (10 mL), 4-nitrobenzyl bromide (621 mg, 2.88 mmol) was then added and the mixture cooled to 0 °C. Sodium hydride (138 mg, 3.46 mmol) was then carefully added and the mixture allowed to warm to room temperature. The reaction mixture was stirred overnight, diluted with CH_2Cl_2 (10 mL) and quenched with water (10 mL). The organics were then dried over $MgSO_4$, reduced *in vacuo*, and purified by flash column chromatography (SiO_2, *n*-Hex/EtOAc, 7:3, *v*/*v*) to yield a white solid (513 mg, 65%).

^{1}H NMR (500 MHz, $CDCl_3$) δ = 8.50 (1, s, 1H), 8.23 – 8.14 (4, m, 2H), 7.46–7.38 (3, m, 2H), 7.13–7.05 (5, 6, m, 4H), 5.06 (2, s, 2H).

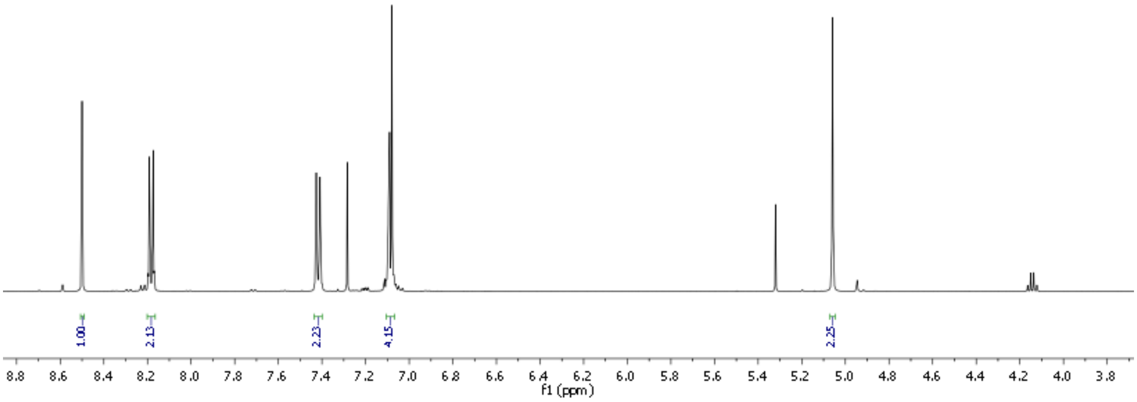

Figure S5 Partial ^{1}H NMR spectrum (500 MHz, 298 K, $CDCl_3$) of compound **S2**.

N-[(4-aminophenyl)methyl]-*N*-(4-fluorophenyl)formamide **Control**-C_1

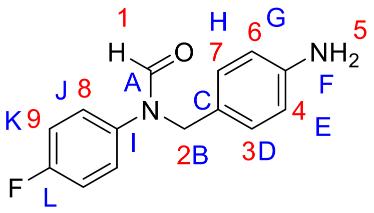

To a reaction flask under a nitrogen atmosphere was added a solution of **S2** (500 mg, 1.82 mmol) in THF (5 mL) and EtOH (5 mL). Palladium on carbon (10 *wt*%) was then added and the reaction mixture placed under a hydrogen environment. The reaction mixture was monitored to completion via TLC and then filtered through Celite. The crude mixture was reduced under pressure and purified by flash column chromatography (SiO_2, CH_2Cl_2/MeOH, 49:1, *v*/*v*) to yield a white solid (329 mg, 74%).

^{1}H NMR (601 MHz, DMSO-d_6) δ = 8.53 (1', s, 1H), 8.47 (1, s, 1H), 7.33–7.28 (8, m, 2H), 7.27–7.24 (8', m, 2H), 7.22–7.17 (9, m, 2H), 7.16–7.12 (9', m, 2H), 6.85 (7', 3', m, 2H), 6.83 (7, 3, m, 2H), 6.46 (6', 4', m, 2H), 6.45–6.42 (6, 4, m, 2H), 5.01 (5', s, 2H), 4.96 (5, s, 2H), 4.79 (2, s, 2H), 4.72 (2', s, 2H).

^{13}C NMR (126 MHz, DMSO-d_6) δ = 163.18 (s), 162.61 (s), 160.61 (d, *J* = 243.2 Hz) 160.26 (d, *J* = 243.2 Hz), 148.56 (s), 148.25 (s), 137.62 (d, *J* = 2.7 Hz), 135.64 (d, *J* = 2.5 Hz), 129.00 (s), 128.97 (s), 128.11 (d, *J* = 8.4 Hz), 126.22 (d, *J* = 8.5 Hz), 123.95 (s), 123.90 (s), 116.45 (d, *J* = 22.6 Hz), 115.80 (d, *J* = 22.4 Hz), 114.29 (s), 114.26 (s), 52.81 (s), 47.13 (s).

^{19}F NMR (376 MHz, DMSO-d_6) δ = −116.10 to −116.19 (minor, m), −116.50 to −116.58 (major, m).

EI HRMS: obtained m/z 244.10072 M^+ (expected m/z 244.10064 M^+).

MP: 64–67 °C

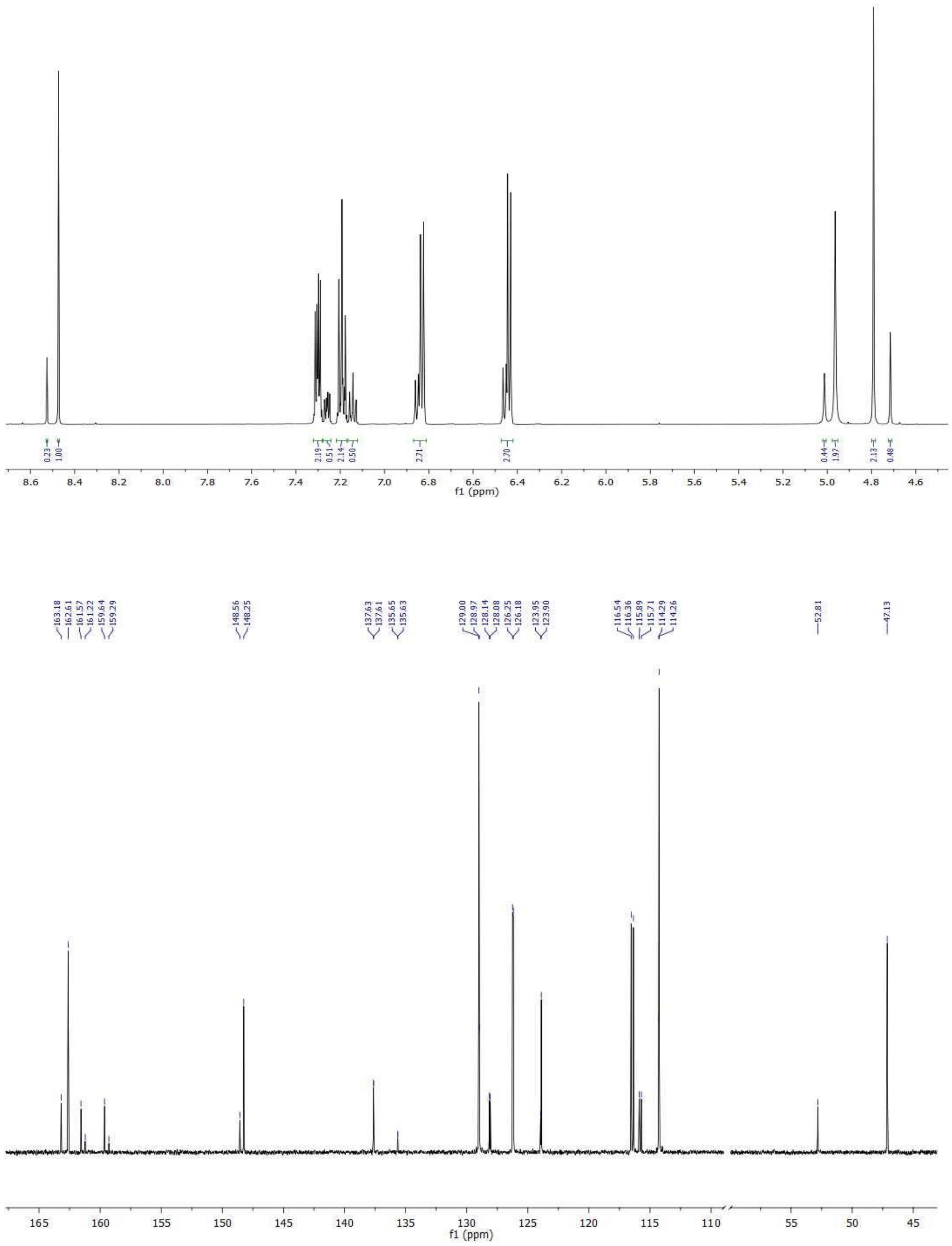

Figure S6 (Top) Partial ^{1}H NMR spectrum (500 MHz, 298 K, DMSO-d_6) and (Bottom) partial ^{13}C NMR spectrum (126 MHz, 298 K, DMSO-d_6) of compound **Control**-C_1.

General Buchwald-Hartwig coupling

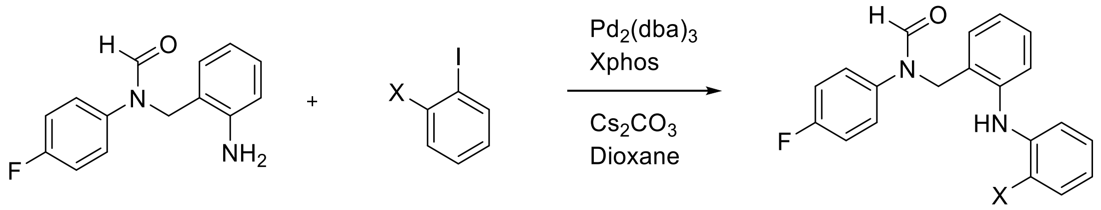

Figure S7 General scheme for Buchwald-Hartwig couplings.

To a flask under a nitrogen atmosphere was added a solution of **1**-C_1-H in degassed dioxane (10 mL). The appropriately substituted iodobenzene was then added along with caesium carbonate, XPhos and $Pd_2(dba)_3$. The reaction mixture was then refluxed overnight and then filtered through celite. Water was added and then the reaction mixture was extracted with CH_2Cl_2 (2 × 10 mL), dried over $MgSO_4$, concentrated under reduced pressure and purified by flash column chromatography.

N-[(2-anilinophenyl)methyl]-*N*-(4-fluorophenyl)formamide **1**-C_1-Ph

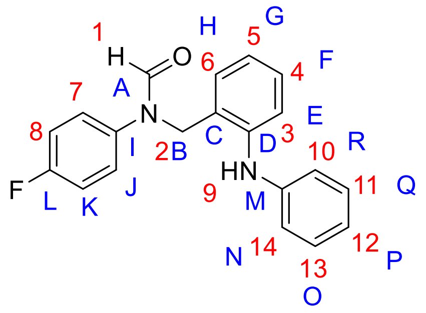

Prepared according to the general Buchwald-Hartwig coupling procedure described above. Dioxane (10 mL), **1**-C_1-H (200 mg, 0.819 mmol), iodobenzene (284 mg, 155 μL, 1.39 mmol), Cs_2CO_3 (800 mg, 2.46 mmol), XPhos (39.0 mg, 0.0820 mmol) and $Pd_2(dba)_3$ (0.0450 mg, 0.0491 mmol). Purification by flash column chromatography (SiO_2, *n*-Hex/EtOAc, 1:1 *v*/*v*) yielded a yellow solid (163 mg, 62%).

^{1}H NMR (500 MHz, DMSO-d_6) δ = 8.58 (1, s, 1H), 8.46 (1', s, 1H), 7.57 (9, s, 1H), 7.36–7.31 (7, m, 2H), 7.27–7.23 (7', m, 2H), 7.23–7.09 (8, m, 2H, 8', m, 2H, 3, m, 1H, 3', m, 1H, 14, 10, m, 2H, 14', 10', m, 2H), 7.08–7.04 (6, m, 1H, 6', m, 1H), 6.96–6.85 (5, m, 1H, 5', m, 1H, 11, 13, m, 2H, 11', 13', m, 2H), 6.83–6.75 (12, m, 1H, 12', m, 1H), 4.97 (2, s, 2H), 4.94 (2', s, 2H).

^{13}C NMR (126 MHz, DMSO-d_6) δ = 163.43 (s), 163.27 (s), 160.67 (d, *J* = 243.2 Hz), 145.54 (s), 145.00 (s), 141.55 (s), 141.35 (s), 137.53 (d, *J* = 2.8 Hz), 135.52 (d, *J* = 2.5 Hz), 129.81 (s), 129.61 (s), 129.42 (s), 128.96 (s), 128.89 (s), 128.44 (s), 128.27–128.14 (m), 127.77 (s), 127.72 (s), 125.81 (d, *J* = 8.5 Hz), 122.99 (s), 122.24 (s), 122.06 (s), 120.32 (s), 119.82 (s), 119.44 (s), 116.73 (s), 116.56 (d, *J* = 22.6 Hz), 116.11 (s), 115.77 (d, *J* = 22.3 Hz), 49.52 (s), 44.59 (s).

^{19}F{^{1}H} NMR (471 MHz, DMSO-d_6) δ −116.11 (minor), −116.30 (major).

EI HRMS: obtained m/z 320.13189 M^+ (expected m/z 320.13194 M^+).

MP: 97–99 °C

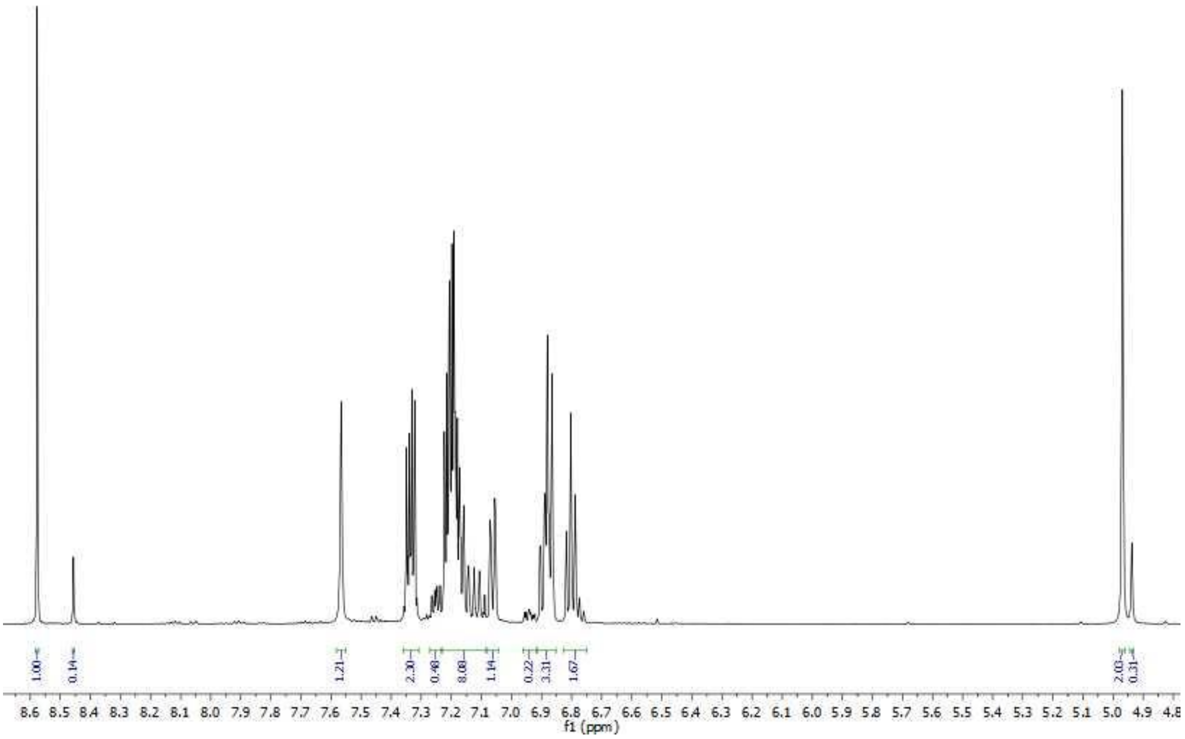

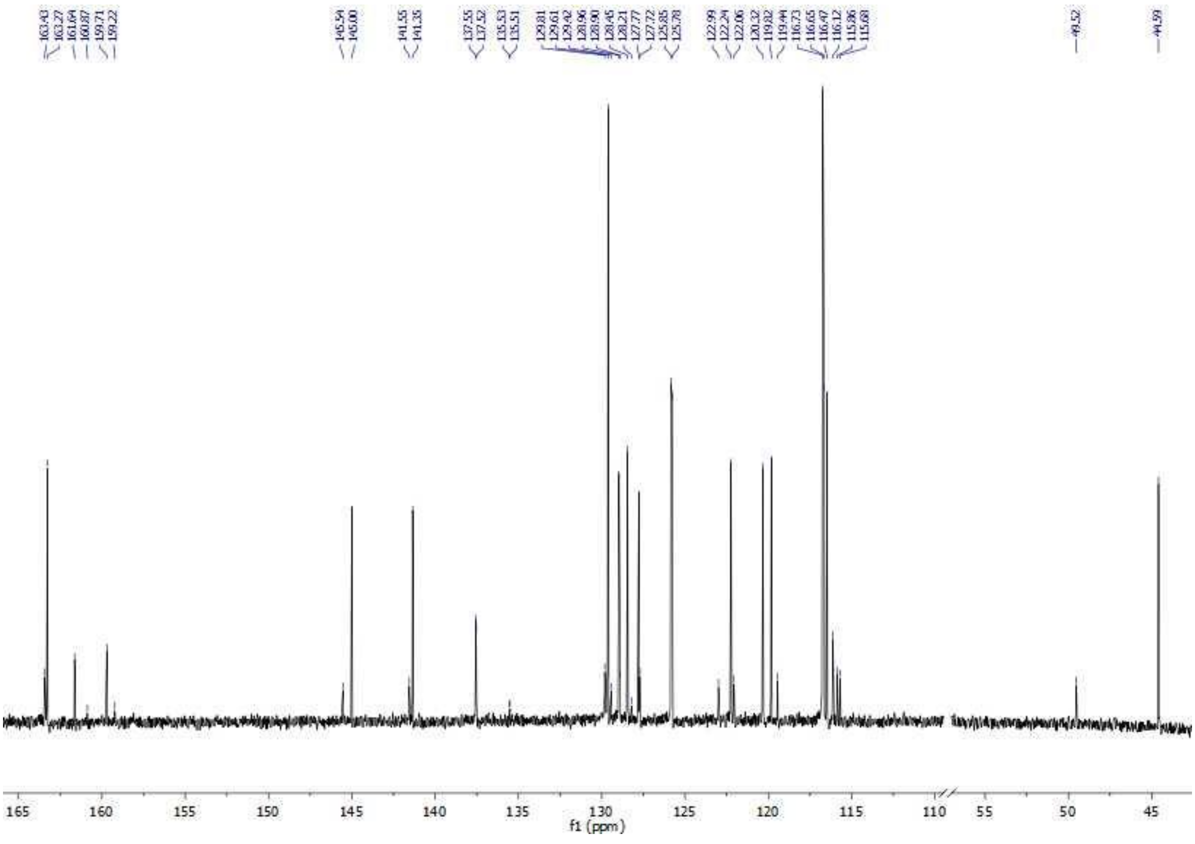

Figure S8 (Top) Partial ^{1}H NMR spectrum (500 MHz, 298 K, DMSO-d_6) and (Bottom) partial ^{13}C NMR spectrum (126 MHz, 298 K, DMSO-d_6) of compound **1**-C_1-Ph.

N-(4-fluorophenyl)-*N*-[[2-(2-methoxyanilino)phenyl]methyl]formamide **1**-C_1-PhOMe

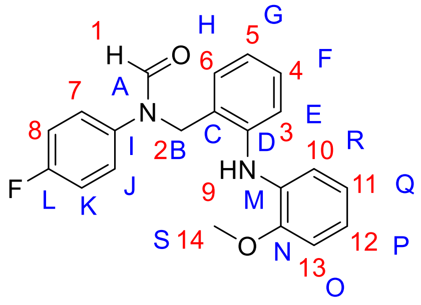

Prepared according to the general Buchwald-Hartwig coupling procedure described above. Dioxane (10 mL), **1**-C_1-H (200 mg, 0.819 mmol), 2-methoxyiodobenzene (325 mg, 182 µL, 1.39 mmol), Cs_2CO_3 (800 mg, 2.46 mmol), XPhos (39.0 mg, 0.0820 mmol) and $Pd_2(dba)_3$ (0.0450 mg, 0.0491 mmol). Purification by flash column chromatography (SiO_2, *n*-Hex/EtOAc, 1:1 *v*/*v*) yielded a brown oil (278 mg, 76%)

^{1}H NMR (500 MHz, DMSO-*d*$_6$) δ = 8.54 (1, s, 1H), 8.47 (1', s, 1H), 7.35–7.30 (7, m, 2H), 7.28–7.24 (7', m, 2H), 7.23–7.17 (8, m, 2H), 7.16–7.09 (8', m, 2H, 6', m, 1H, 4, m, 1H, 4', m, 1H), 7.08–7.04 (6, m, 1H), 7.04–6.98 (3, m, 1H, 3', m, 1H, 10, m, 1H, 10', m, 1H), 6.95–6.77 (5, m, 1H, 13, m, 1H, 11, m, 1H, 12, m, 1H, 5', m, 1H, 13', m, 1H, 11', m, 1H, 12', m, 1H), 6.70–6.67 (13', m, 1H), 4.97 (2, s, 2H), 4.96 (2,' s, 2H), 3.84 (14', s, 1H), 3.83 (14, s, 3H).

^{13}C NMR (126 MHz, DMSO-*d*$_6$) δ = 163.41 (s), 163.10 (s), 160.74 (d, *J* = 243.3 Hz), 160.27 (d, *J* = 243.2 Hz), 149.70 (s), 149.01 (s), 141.84 (s), 137.34 (d, *J* = 2.8 Hz), 135.39 (d, 2.5 Hz), 134.16 (s), 133.62 (s), 130.17 (s), 129.74 (s), 128.91 (s), 128.84 (s), 128.54 (s), 127.91 (d, *J* = 8.5 Hz), 127.14 (s), 126.21 (d, *J* = 8.6 Hz), 122.67 (s), 121.85 (s), 121.77 (s), 121.15 (s), 121.10 (s), 120.97 (s), 120.32 (s), 119.91 (s), 116.81 (s), 116.54 (d, *J* = 22.6 Hz), 115.76 (d, *J* = 22.5 Hz), 115.39 (s), 111.90 (s), 111.71 (s), 56.03 (s), 49.91 (s), 44.68 (s).

^{19}F{^{1}H} NMR (471 MHz, DMSO-*d*$_6$) δ = −116.05 (minor), −116.16 (major).

EI HRMS: obtained m/z 350.14288 M$^+$ (expected m/z 350.14251 M$^+$).

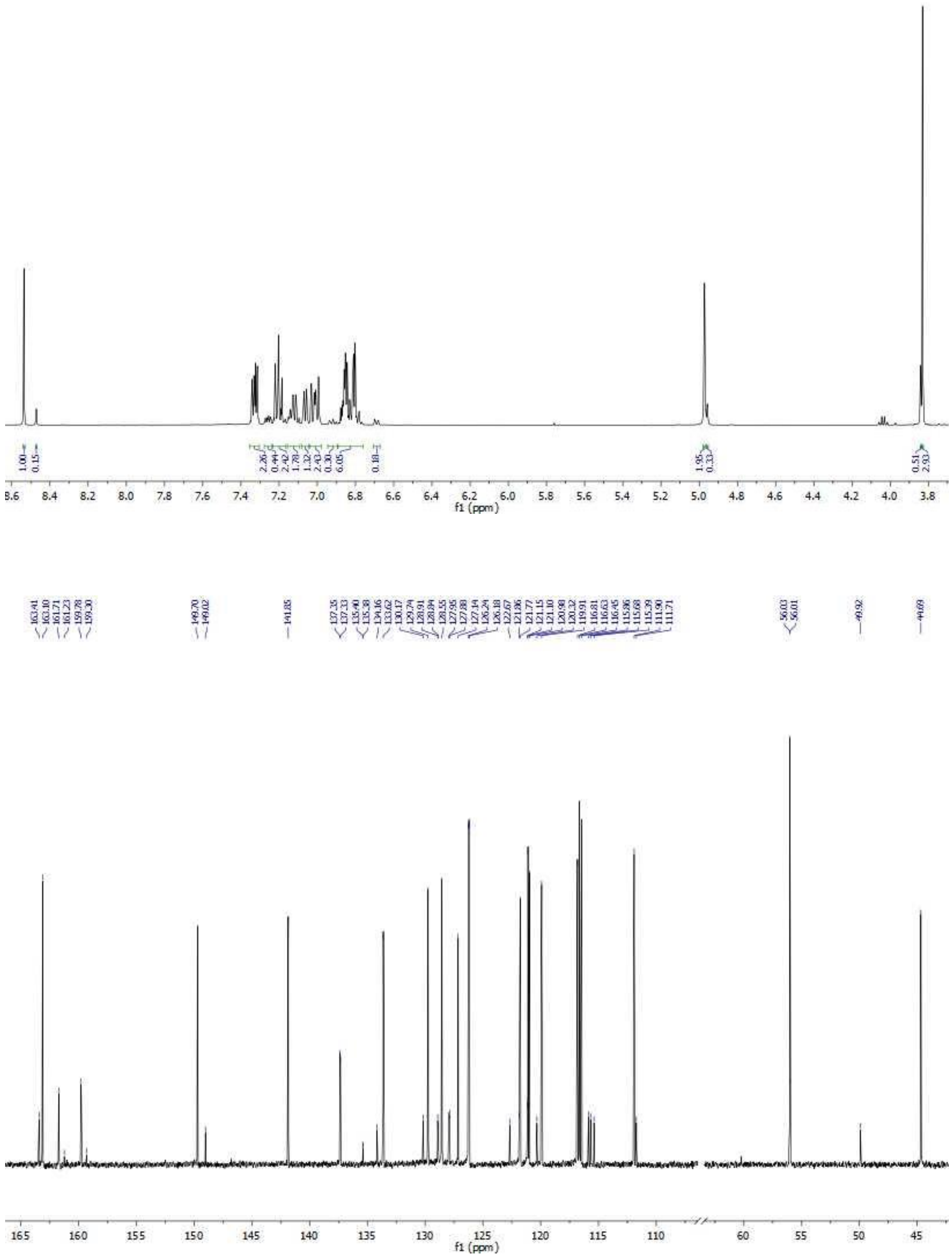

Figure S9 (Top) Partial ^{1}H NMR spectrum (500 MHz, 298 K, DMSO-d_6) and (Bottom) partial ^{13}C NMR spectrum (126 MHz, 298 K, DMSO-d_6) of compound **1**-C_1-PhOMe.

N-[[2-(2-cyanoanilino)phenyl]methyl]-*N*-(4-fluorophenyl)formamide **1**-C_1-PhCN

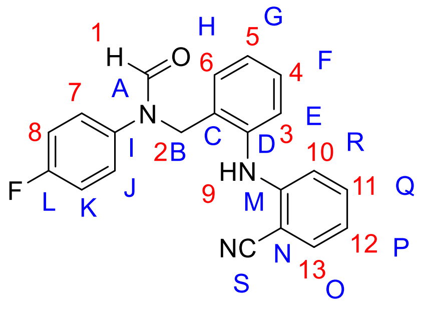

Prepared according to the general Buchwald-Hartwig coupling procedure described above. Dioxane (10 mL) **1**-C_1-H (200 mg, 0.819 mmol), 2-iodobenzonitrile (318 mg, 1.39 mmol), Cs_2CO_3 (800 mg, 2.46 mmol), XPhos (39.0 mg, 0.0820 mmol) and $Pd_2(dba)_3$ (0.0450 mg, 0.0491 mmol). Purification by flash column chromatography (SiO_2, *n*-Hex/EtOAc, 1:1 *v*/*v*) yielded a brown oil (206 mg, 73%).

^{1}H NMR (500 MHz, DMSO-d_6) δ = 8.54 (1, s, 1H), 8.40 (1', s, 1H), 8.02 (9', s, 1H), 7.98 (9, s, 1H), 7.63–7.60 (13, m, 1H, 13', m, 1H), 7.43–7.35 (11, m, 1H, 11', m, 1H), 7.33–7.28 (7, m, 2H, 7', m, 2H), 7.28–7.23 (4, m, 2H, 4', m, 2H), 7.20–7.06 (6, m, 1H, 8, m, 2H, 5, m, 1H, 3, m, 1H, 6', m, 1H, 8', m, 2H, 5', m, 1H, 3', m, 1H), 6.91–6.85 (12, m, 1H, 12', m, 1H), 6.61–6.58 (10, m, 1H), 6.48–6.46 (10', s, 1H), 4.95 (2, s, 2H), 4.93 (2', s, 2H).

^{13}C NMR (126 MHz, DMSO-d_6) δ = 163.21 (s), 163.09 (s), 160.62 (d, *J* = 243.2 Hz), 160.20 (d, *J* = 243.2 Hz), 149.19 (s), 148.91 (s), 139.77 (s), 139.53 (s), 137.52 (d, *J* = 2.7 Hz), 135.40 (d, 2.5 Hz), 134.66 (s), 134.26 (s), 134.23 (s), 132.47 (s), 131.34 (s), 130.10 (s), 129.26 (s), 129.21 (s), 128.78 (s), 127.73 (d, *J* = 8.5 Hz), 126.22 (s), 125.76 (s), 125.63 (d, *J* = 8.5 Hz), 125.31 (s), 125.04 (s), 119.66 (s), 119.41 (s), 118.20 (s), 118.10 (s), 116.52 (d, *J* = 22.7 Hz), 116.20 (s), 115.87 (s), 115.75 (d, *J* = 22.5 Hz), 98.93 (s), 98.31 (s), 49.43 (s), 44.44 (s).

^{19}F{^{1}H} NMR (471 MHz, DMSO-d_6) δ = −116.10 (minor), −116.42 (major).

EI HRMS: obtained m/z 345.12864 M^+ (expected m/z 345.12719 M^+).

MP: 90–92 °C.

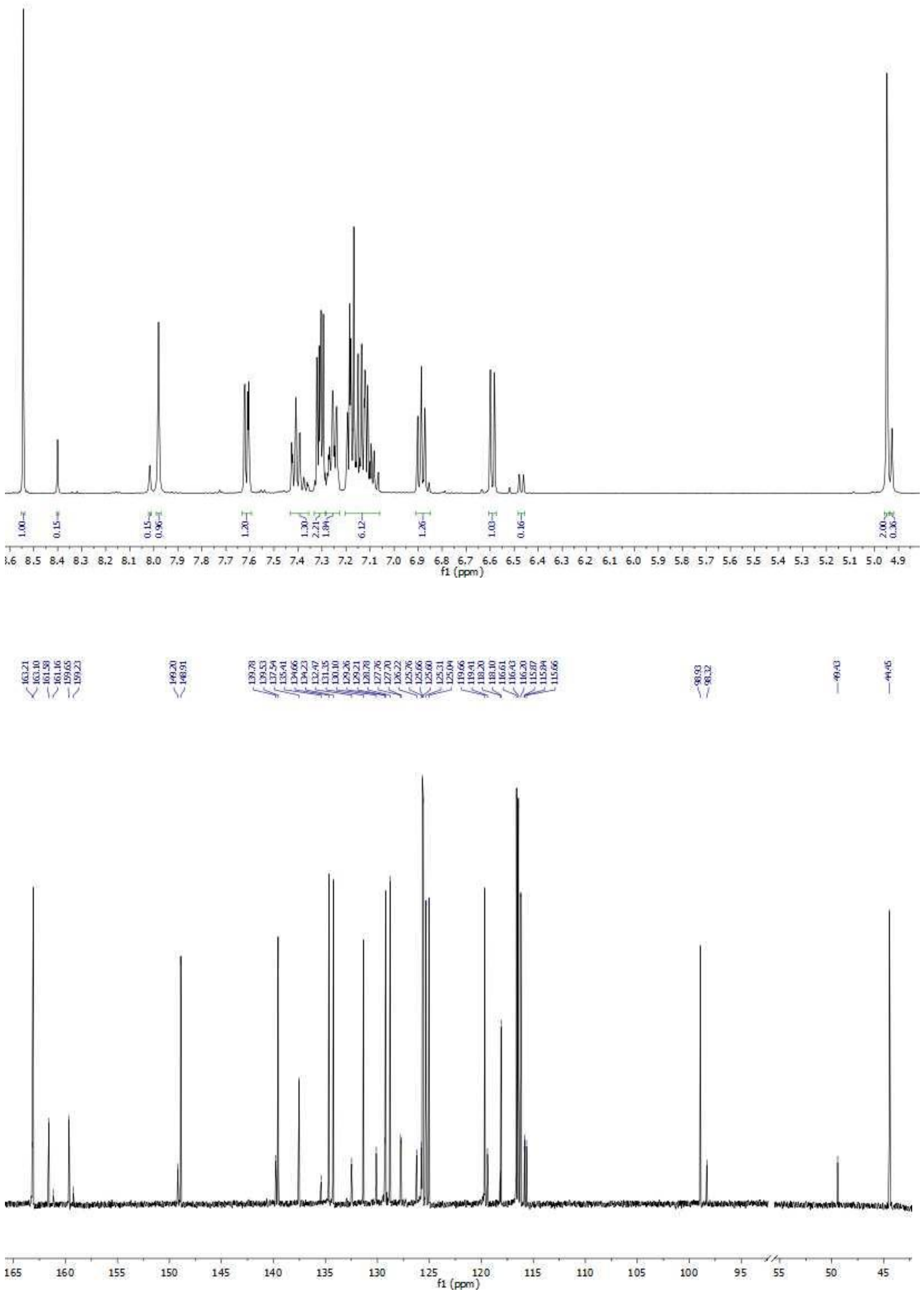

Figure S10 (Top) Partial ^{1}H NMR spectrum (500 MHz, 298 K, DMSO-d_6) and (Bottom) partial ^{13}C NMR spectrum (126 MHz, 298 K, DMSO-d_6) of compound **1**-C_1-PhCN.

General amide couplings

General acid chloride coupling

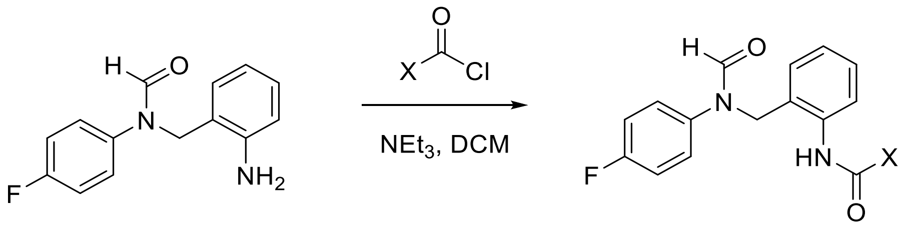

Figure S11 General scheme for acid chloride coupling.

To a solution of **1**-C_1-H in dry CH_2Cl_2 was added an appropriately substituted acid chloride. Triethylamine was then added and the reaction mixture heated to reflux. The reaction mixture was monitored to completion via TLC and then diluted with CH_2Cl_2 and washed with saturated Na_2HCO_3. The aqueous phase was extracted with CH_2Cl_2 and the combined organic phases were then washed with water and brine, dried over $MgSO_4$ and concentrated *in vacuo*. The crude product was then purified by flash column chromatography.

General propyl phosphonic anhydride coupling

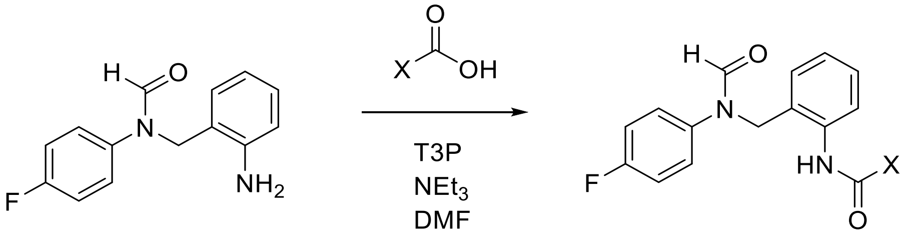

Figure S12 General scheme for propylphosphonic acid coupling.

To a solution of **1**-C_1-H in dry DMF was added an appropriately substituted acid. Propylphosphonic anhydride (T3P) solution was then added followed by triethylamine. The reaction mixture was stirred overnight at room temperature and then extracted with CH_2Cl_2, washed with 2 M HCl, water and brine. The combined organic phases were then dried over $MgSO_4$ and concentrated *in vacuo*. The crude product was then purified by flash column chromatography.

N-[2-[(4-fluoro-*N*-formyl-anilino)methyl]phenyl]acetamide **2**-C_1-Me

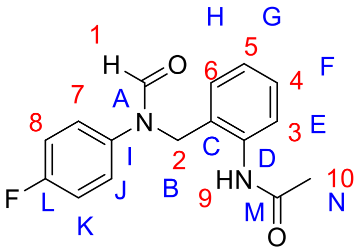

Prepared according to general acid chloride coupling procedure described above. CH_2Cl_2 (10 mL), **1**-C_1-H (670 mg, 2.74 mmol), acetyl chloride (290 µL, 4.12 mmol) and triethylamine (770 µL, 5.49 mmol). Purification by flash column chromatography (SiO_2, *n*-Hex/EtOAc, 1:1, *v*/*v*) yielded a white solid (490 mg, 62%).

^{1}H NMR (601 MHz, DMSO-d_6) δ = 9.58 (9', s, 1H) 9.49 (9, s, 1H), 8.55 (1, s, 1H), 8.49 (1', s, 1H), 7.40 (3, d, *J* = 7.9 Hz, 1H), 7.37–7.32 (7, m, 2H), 7.28–7.25 (7', m, 2H), 7.24–7.19 (8, 2H, 5, 1H, 3', 1H, 5', 1H), 7.15-7.10 (8', m, 2H, 6', m, 1H, 4', m, 1H), 7.05–7.09 (6, 1H, 4, 1H), 4.95 (s, 2, 2H), 4.90 (s, 2', 2H), 2.05 (10, s, 3H), 2.05 (10', s, 3H).

^{13}C NMR (126 MHz, DMSO-d_6) δ = 168.89 (M, s), 163.42 (A', s), 163.19 (A, s), 160.70 (L, d, *J* = 243.2 Hz), 160.27 (L', d, *J* = 244.4 Hz), 137.48 (I, d, *J* = 2.9 Hz), 136.49 (D', s), 136.45 (D, s), 135.44 (I, d, *J* = 2.5 Hz), 131.81 (C', s), 130.48 (C, s), 129.16 (H', s), 128.53 (H, s), 128.33 (G', s), 127.97 (G, s) 127.88 (J', d, *J* = 8.4 Hz), 126.95 (E', s), 126.10 (F', s), 125.95 (J, d, *J* = 8.5 Hz), 125.68 (E, s) 125.54 (F, s) 116.56 (K, d, *J* = 22.6 Hz) 115.80 (K', d) 50.02 (B', s), 44.69 (B, s), 23.78 (N, s), 23.54 (N', s).

^{19}F{^{1}H} NMR (471 MHz, DMSO-d_6) δ = −116.02 (minor), −116.27 (major).

EI HRMS: obtained m/z 309.10150 M^+ (expected m/z 309.10098 M^+).

MP: 106–108 °C.

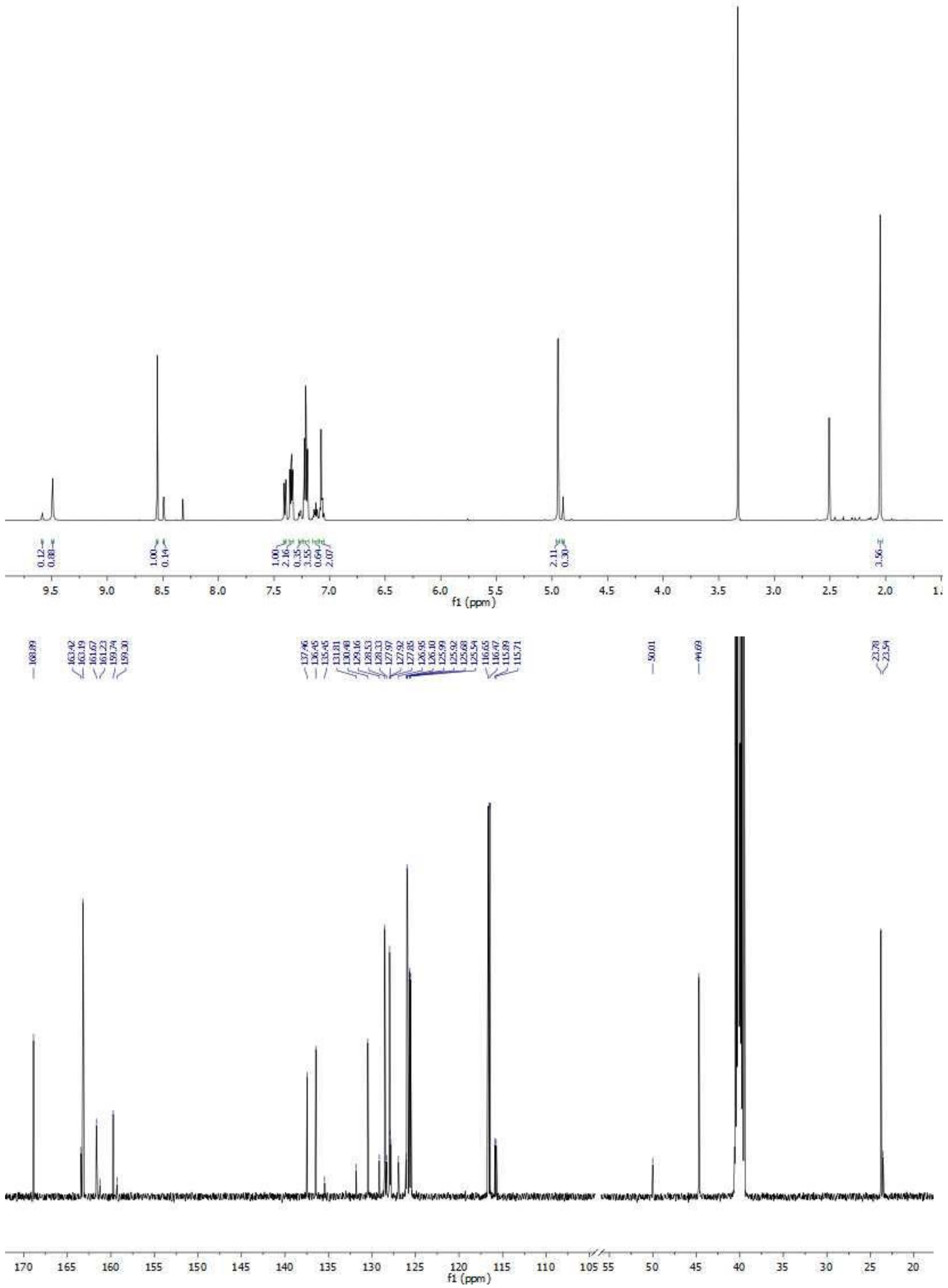

Figure S13 (Top) Partial ^{1}H NMR spectrum (500 MHz, 298 K, DMSO-d_6) and (Bottom) partial ^{13}C NMR spectrum (126 MHz, 298 K, DMSO-d_6) of compound **2**-C_1-Me.

2,2,2-trifluoro-*N*-[2-[(4-fluoro-*N*-formyl-anilino)methyl]phenyl]acetamide **2**-C_1-CF_3

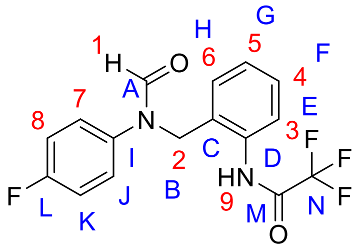

Prepared according to general acid chloride coupling procedure described above. CH_2Cl_2 (5 mL), **1**-C_1-H (240 mg, 0.982 mmol), trifluoroacetic anhydride (140 µL, 1.47 mmol) and triethylamine 140 µL, 1.94 mmol). Purification by flash column chromatography (SiO_2, *n*-Hex/EtOAc, 1:1, *v*/*v*) yielded a white solid (180 mg, 55%).

^{1}H NMR (601 MHz, DMSO-d_6) δ = 11.08 (9, s, 1H), 8.50 (1, s, 1H), 8.46 (1', s, 1H), 7.36–7.31 (7, m, 2H, 3, m, 1H, 5, m, 1H, 3', m, 1H), 7.30–7.18 (8, m, 2H, 6, m, 1H, 4, m, 1H, 6', m, 1H, 4', m, 1H), 7.15–7.12 (8', m, 2H), 4.95 (2, s, 2H), 4.93 (2', s, 2H).

^{13}C NMR (126 MHz, DMSO-d_6) δ = 163.23 (A', s), 163.04 (A, s), 160.80 (L, d, *J* = 243.4 Hz), 160.39 (L', d, *J* = 243.2 Hz), 155.94 (q, *J* = 36.8 Hz), 137.26 (I, d, *J* = 2.8 Hz), 135.15 (I', d, *J* = 3.7 Hz), 133.66 (D', s), 133.40 (D, s), 132.97 (C', s), 132.59 (C, s), 129.90 (s), 129.23 (s), 128.91 (s), 128.55 (s), 128.15 (s), 128.07 (s), 127.99 (s), 127.92 (s), 127.41 (s), 126.24 (J, d, *J* = 8.5 Hz), 119.90 (s), 117.61 (s), 116.58 (d, *J* = 22.7 Hz), 115.88 (d, *J* = 22.5 Hz), 115.31 (s), 113.02 (s), 49.60 (s), 44.38 (s).

^{19}F{^{1}H} NMR (471 MHz, DMSO-d_6) δ = −73.71 (C*F*$_3$', minor), −73.87 (C*F*$_3$ major), −115.84 (C*F* minor), −116.09 (C*F* major).

EI HRMS: obtained m/z 340.08245 M^+ (expected m/z 340.08294 M^+).

MP: 130–132 °C.

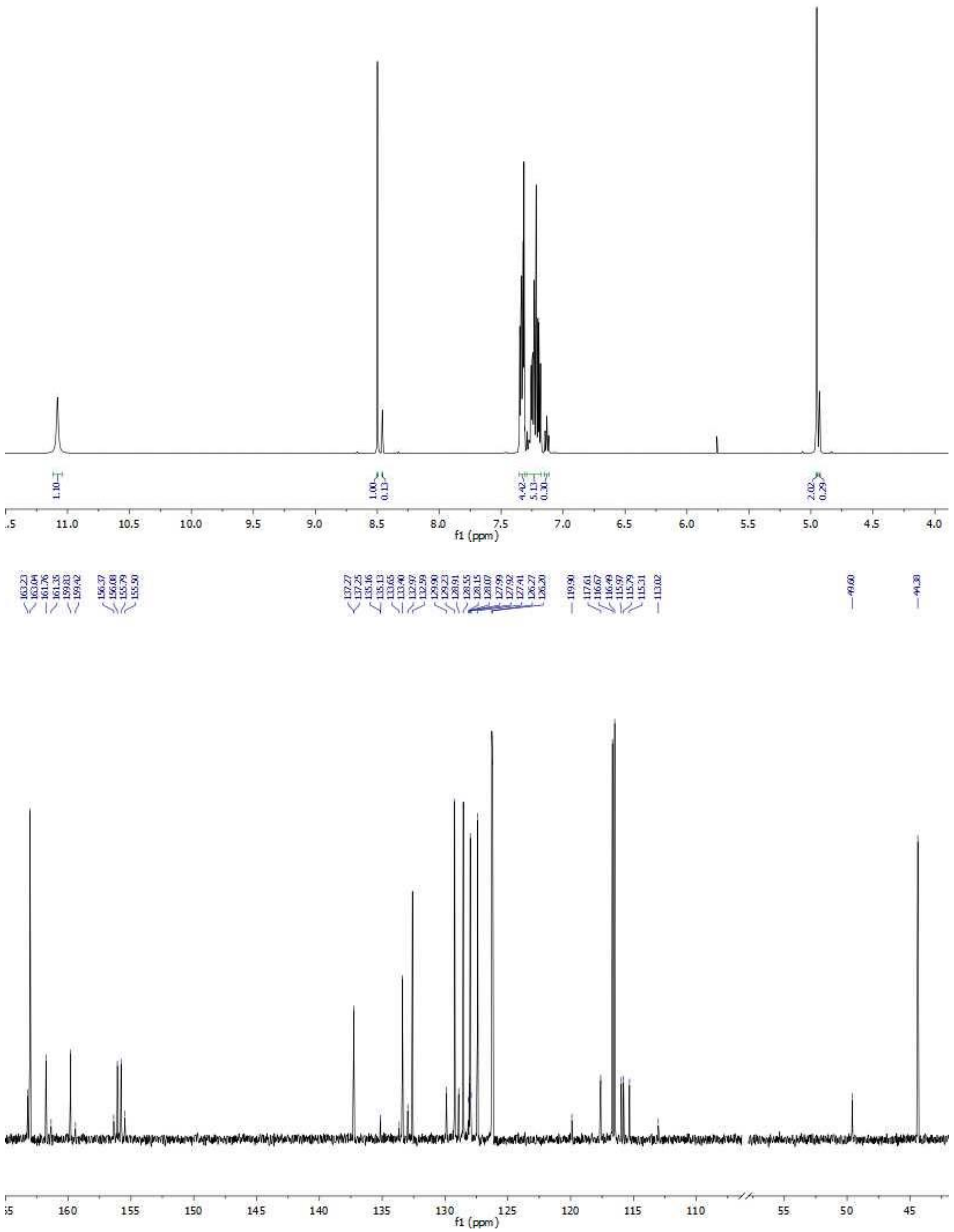

Figure S14 (Top) Partial ^{1}H NMR spectrum (500 MHz, 298 K, DMSO-d_6) and (Bottom) partial ^{13}C NMR spectrum (126 MHz, 298 K, DMSO-d_6) of compound **2**-C_1-CF_3.

N-[2-[(4-fluoro-*N*-formyl-anilino)methyl]phenyl]-2,2-dimethyl-propanamide **2**-C_1-^tBu

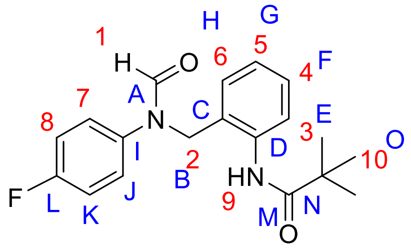

Prepared according to general acid chloride coupling procedure described above. CH_2Cl_2 (10 mL), **1**-C_1-H (700 mg, 2.87 mmol), trimethylacetyl chloride (530 µL, 4.31 mmol) and triethylamine (800 µL, 5.73 mmol). Purification by flash column chromatography (SiO_2, *n*-Hex/EtOAc, 1:1, *v*/*v*) yielded a white solid (850 mg, 90%).

^{1}H NMR (601 MHz, DMSO-d_6) δ = 9.24 (9, s, 1H), 9.15 (9', s, 1H), 8.57 (1, s, 1H), 8.46 (1', s, 1H), 7.36–7.32 (7, m, 2H), 7.30–7.21 (8, m, 2H, 7', m, 2H, 3, m, 1H, 4, m, 1H, 3', m, 1H), 7.17–7.09 (8', m, 2H, 6', m, 1H, 4', m, 1H, 5, m, 1H, 5', m, 1H), 7.05–7.04 (6, m, 1H), 4.87 (2, s, 2H), 4.84 (2', s, 2H), 1.27 (10, s, 9H), 1.25 (10', s, 9H).

^{13}C NMR (126 MHz, DMSO-d_6) δ = 177.37 (M', s), 177.19 (M, s), 163.54 (A', s), 163.22 (A, s), 160.71 (L, d, *J* = 243.2 Hz), 137.66 (I, d, *J* = 2.6 Hz), 136.79 (D', s), 136.48 (D, s), 135.57 (I', d, *J* = 2.5 Hz), 133.23 (C', s), 131.80 (C, s), 128.74 (H', s), 128.17 (E', s), 128.00 (H, s), 127.93 (G', s), 127.77 (F, s), 127.64 (J, d, *J* = 8.4 Hz), 127.21 (E, s), 126.49 (F', s), 126.01 (G, s), 125.82 (J, d, *J* = 8.5 Hz), 116.62 (K, d, *J* = 22.7 Hz), 115.82 (K', d, *J* = 22.5 Hz), 49.86 (B', s), 44.74 (B, s), 39.30 (N, s), 39.16 (N', s), 27.81 (O', s), 27.79 (O, s).

^{19}F{^{1}H} NMR (471 MHz, DMSO-d_6) δ = −116.11 (minor), −116.30 (major).

EI HRMS: obtained m/z 328.15717 M^+ (expected m/z 328.15816 M^+).

MP: 160–161 °C.

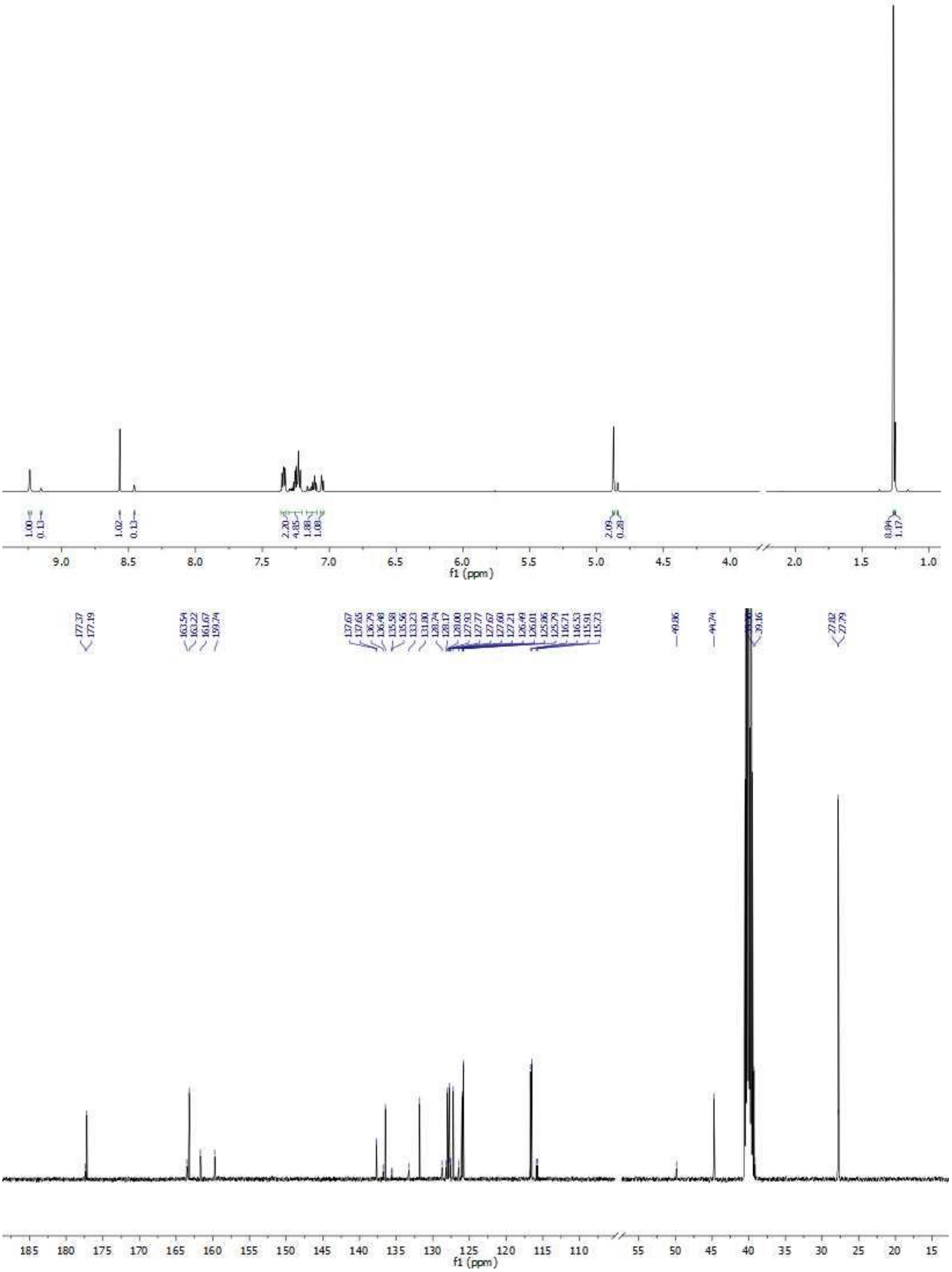

Figure S15 (Top) Partial ^{1}H NMR spectrum (500 MHz, 298 K, DMSO-d_6) and (Bottom) partial ^{13}C NMR spectrum (126 MHz, 298 K, DMSO-d_6) of compound **2**-C_1-^{t}Bu.

N-[2-[(4-fluoro-*N*-formyl-anilino)methyl]phenyl]benzamide **2**-C_1-Ph

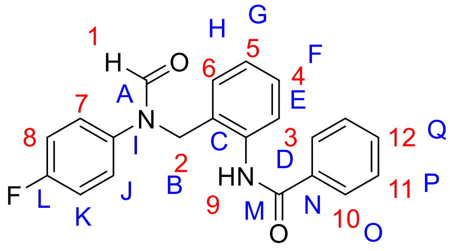

Prepared according to general acid chloride coupling procedure described above. CH_2Cl_2 (10 mL), **1**-C_1-H (1.03 g, 4.21 mmol), benzoyl chloride (730 μL, 6.33 mmol) and triethylamine (1.18 mL, 8.40 mmol). Purification by flash column chromatography (SiO_2, *n*-Hex/EtOAc, 1:1, *v*/*v*) yielded a white solid (840 mg, 57%).

^{1}H NMR (601 MHz, DMSO-d_6) δ = 10.12 (9, s, 1H), 8.54 (1, s, 1H), 8.40 (1', s, 1H), 8.03–8.00 (10', m, 1H), 8.00–7.98 (10', m, 2H), 7.63–7.59 (12, m, 1H, 12', m, 1H), 7.57–7.52 (11, m, 2H, 11', m, 2H), 7.43 (3, m, 1H), 7.39–7.34 (7, m, 2H), 7.32–7.27 (5, m, 1H, 7', m, 2H), 7.23–7.10 (8, m, 2H, 4, m, 1H, 6, m, 1H, 6', m, 1H, 8', m, 2H, 4', m, 1H), 5.01 (2, s, 2H), 4.97 (2', s, 2H).

^{13}C NMR (151 MHz, DMSO-d_6) δ = 166.01 (M, s), 163.31 (A', s), 163.18 (A, s), 160.69 (L, d, *J* = 243.2 Hz), 137.63 (I, d, *J* = 2.7 Hz), 136.28 (D, s), 134.72 (N, s), 132. 10 (Q, s), 131.95 (C, s), 128.89 (s), 128.83 (P, s), 128.42 (s), 128.39 (F, s), 128.22 (O, s), 127.96 (G, s), 127.84 (J', d, *J* = 8.4 Hz), 127.14 (E, s), 126.32 (H, s), 125.95 (J, d, *J* = 8.5 Hz), 116.57 (K, d, *J* = 22.6 Hz), 115.80 (K', d, *J* = 22.4 Hz), 44.91 (B, s), 40.55 (B', s).

^{19}F{^{1}H} NMR (471 MHz, DMSO-d_6) δ = −116.06 (minor), −116.30 (major).

EI HRMS: obtained m/z 348.12830 M^+ (expected m/z 348.12686 M^+).

MP: 183–185 °C.

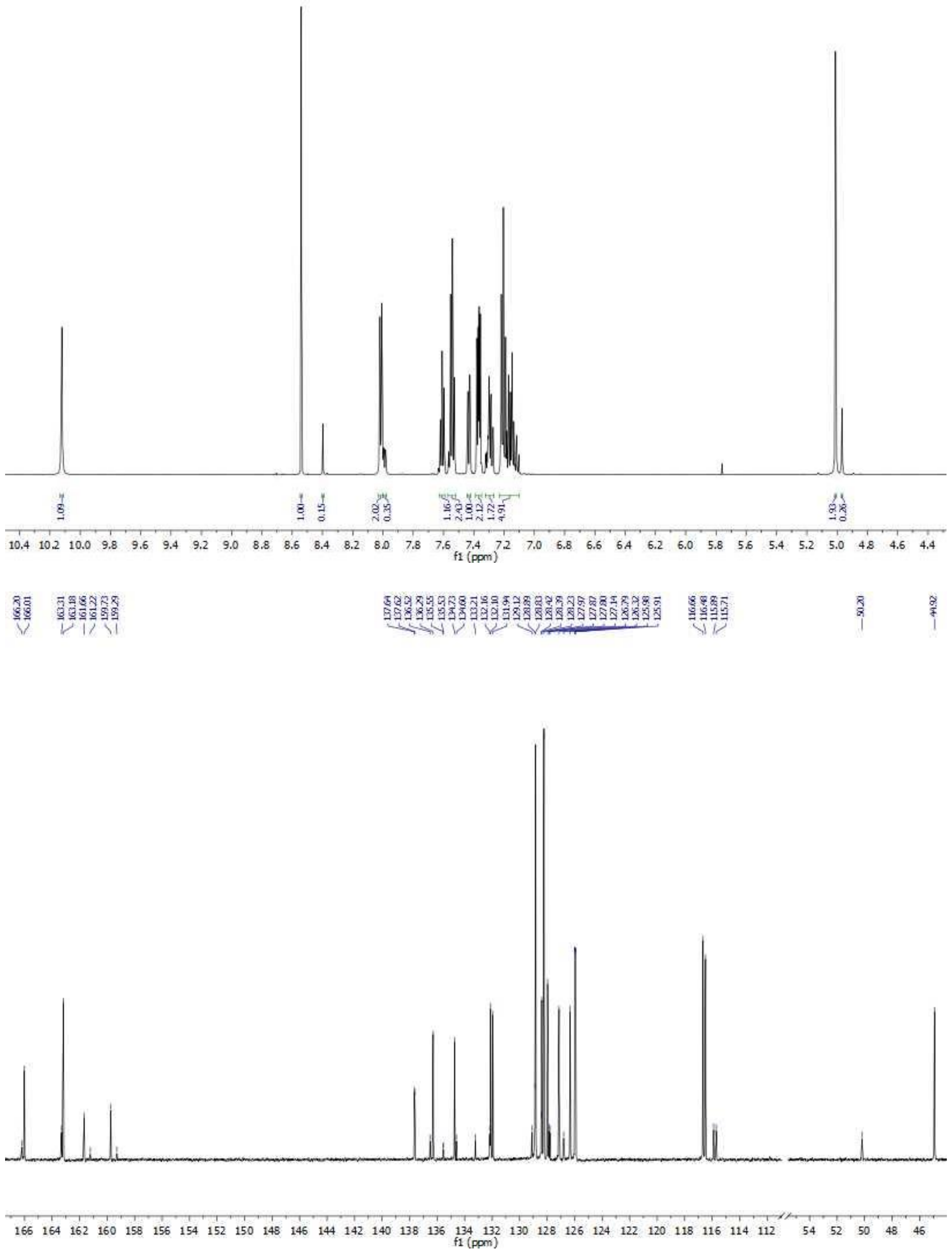

Figure S16 (Top) Partial ^{1}H NMR spectrum (500 MHz, 298 K, DMSO-d_6) and (Bottom) partial ^{13}C NMR spectrum (126 MHz, 298 K, DMSO-d_6) of compound **2**-C_1-Ph.

N-[2-[(4-fluoro-*N*-formyl-anilino)methyl]phenyl]-4-methoxy-benzamide **2**-C_1-PhOMe

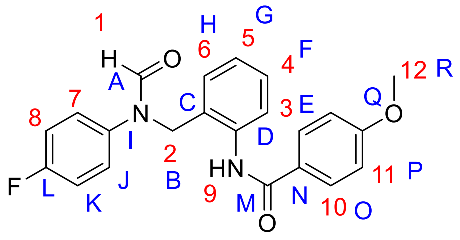

Prepared according to general acid chloride coupling procedure described above. CH_2Cl_2 (15 mL), **1**-C_1-H (700 mg, 2.87 mmol), 4-methoxybenzoyl chloride (730 g, 4.28 mmol) and triethylamine (430 μL, 3.44 mmol). Purification by flash column chromatography (SiO_2, CH_2Cl_2/MeOH 24:1, *v*/*v*) yielded a white solid (800 mg, 74%).

^{1}H NMR (601 MHz, DMSO-*d*$_6$) δ = 9.98 (9, s, 1H), 8.54 (1, s, 1H), 8.38 (1', s, 1H), 8.01–7.97 (10, m, 2H, 10', m, 2H), 7.43–7.42 (3, m, 1H), 7.38–7.34 (7, m, 2H), 7.31–7.26 (4, m, 1H, 7', m, 2H, 3', m, 1H), 7.23–7.18 (8, m, 2H, 6', m, 1H, 4', m, 1H), 7.17–7.11 (8', m, 2H, 6, m, 2H, 5, m, 1H, 5', m, 1H), 7.10–7.05 (11, m, 2H, 11', m, 2H), 4.99 (2, s, 1H), 4.95 (2', s, 1H), 3.86 (12', s, 3H), 3.85 (12, s, 3H).

^{13}C NMR (126 MHz, DMSO-*d*$_6$) δ = 165.58 (M', s), 165.41 (M, s), 163.27 (A', s), 163.18 (A, s), 162.46 (Q', s), 162.43 (Q, s), 160.69 (L, d, *J* = 243.2 Hz), 160.25 (L', d, *J* = 243.2 Hz), 137.63 (I, d, *J* = 2.8 Hz), 136.73 (D', s), 136.48 (D, s), 135.54 (I', d, *J* = 2.5 Hz), 133.19 (C', s), 131.81 (C, s), 130.14 (O, s), 129.11 (O', s), 128.38 (H', s), 128.36 (H, s), 127.95 (F', s), 127.92 (F, s), 127.83 (J, d, *J* = 8.4 Hz), 127.07 (E, s), 126.84 (N, s), 126.67 (E', s), 126.62 (G', s), 126.10 (G, s), 125.93 (J, d, *J* = 8.5 Hz), 116.57 (K, d, *J* = 22.6 Hz), 115.78 (K', d, *J* = 22.5 Hz), 114.11 (P', s), 114.05 (P, s), 55.91 (R, s), 55.38 (R', s), 50.28 (B', s), 44.96 (B, s).

^{19}F{^{1}H} NMR (471 MHz, DMSO-*d*$_6$) δ = −116.08 (minor), −116.30 (major).

EI HRMS: obtained m/z 378.13921 M$^+$ (expected m/z 378.13742 M$^+$).

MP: 176–177 °C.

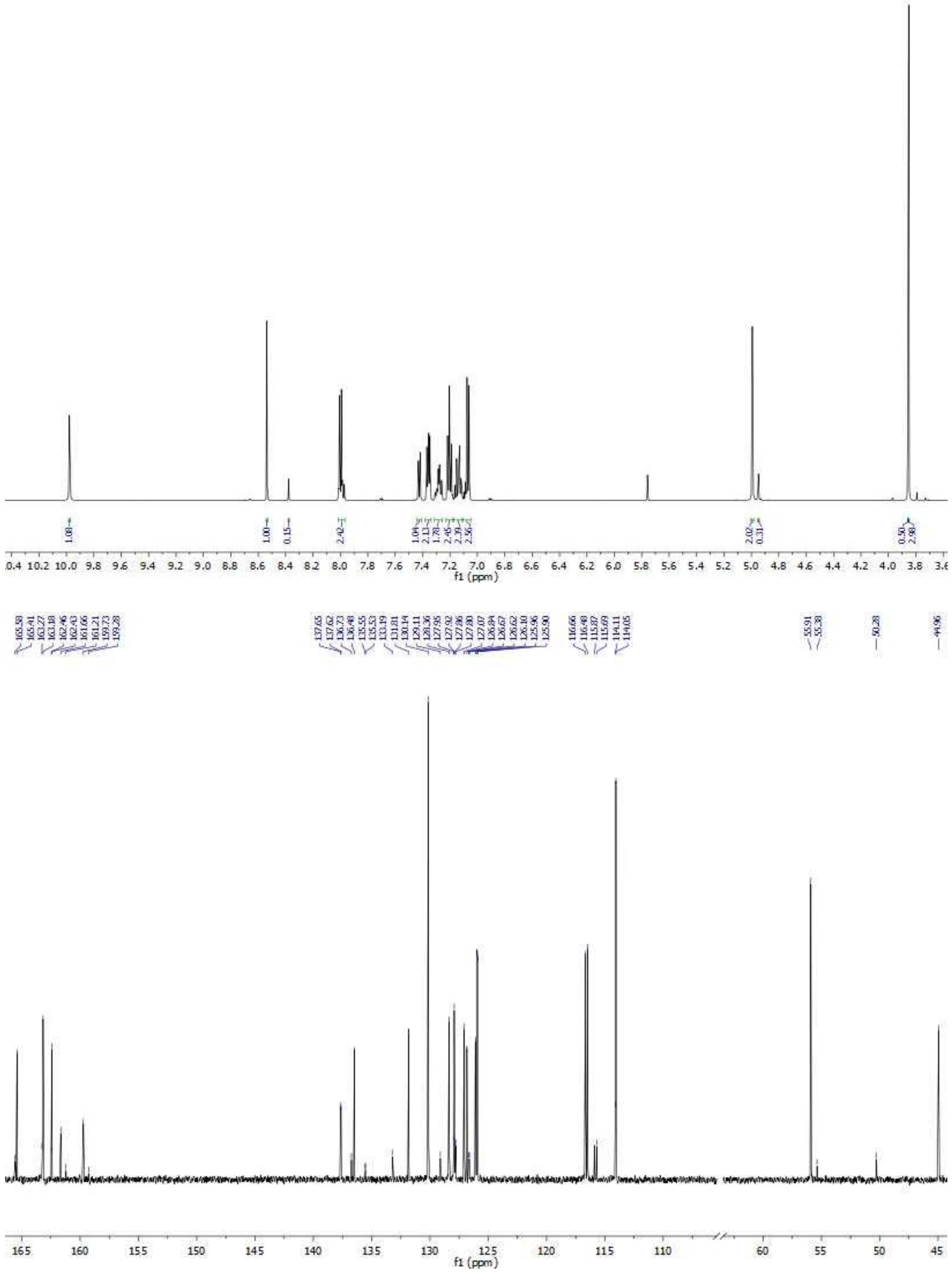

Figure S17 (Top) Partial ^{1}H NMR spectrum (500 MHz, 298 K, DMSO-d_6) and (Bottom) partial ^{13}C NMR spectrum (126 MHz, 298 K, DMSO-d_6) of compound **2**-C_1-PhOMe.

N-[2-[(4-fluoro-*N*-formyl-anilino)methyl]phenyl]pyridine-2-carboxamide **2**-C_1-Pyr

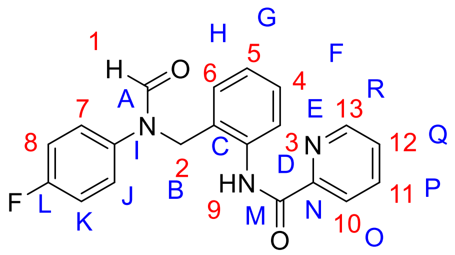

Prepared according to general propylphosphonic anhydride coupling procedure described above. DMF (4 mL), **1**-C_1-H (200 mg, 0.819 mmol), 2-picolinic acid (100 mg, 0.819 mmol), propylphosphonic anhydride (50%, mass) in ethyl acetate (540 µL, 0.900 mmol), triethylamine (100 mg, 140 µL, 0.983 mmol). Purification by flash column chromatography (SiO_2, *n*-Hex/EtOAc, 1:1, *v*/*v*) yielded a white solid (49.0 mg, 17%).

^{1}H NMR (601 MHz, DMSO-*d*$_6$) δ = 10.55 (9', s, 1H), 10.53 (9, s, 1H), 8.76–8.73 (10, m, 1H, 10', m, 1H), 8.56 (1, s, 1H), 8.50 (1', s, 1H), 8.17–8.13 (13, m, 1H, 13', m, 1H), 8.10–8.06 (12, m, 1H, 12', m, 1H), 7.72-7.67 (11, m, 1H, 11', m, 1H), 7.64–7.59 (3, m, 1H, 3', m, 1H), 7.38–7.34 (7, m, 2H), 7.33–7.25 (4, m, 1H, 7', m, 2H, 4', m, 1H), 7.22–7.20 (6, m, 1H, 6', m, 1H), 7.19–7.14 (8, m, 2H, 5, m, 1H, 5', m, 1H), 7.11–7.07 (8', m, 2H), 5.07 (2', s, 2H), 5.04 (2, s, 2H).

^{13}C NMR (151 MHz, DMSO-*d*$_6$) δ = 163.39 (A', s), 163.18 (M, s), 163.11 (A, s), 160.62 (L, d, *J* = 243.2 Hz), 150.11 (s), 149.94 (s), 148.94 (O, s), 138.63 (Q', s), 138.49 (Q, s), 137.54 (I, d, *J* = 2.7 Hz), 136.39 (s), 135.96 (s), 130.87 (s), 128.79 (H, s), 128.05 (F, s), 127.40 (P, s), 126.08 (G', s), 126.06 (G, s), 125.91 (E, s), 125.83 (J, d, *J* = 8.5 Hz), 122.81 (R', s), 122.79 (R, s), 116.52 (K, d, *J* = 22.6 Hz), 115.79 (K', d, *J* = 22.1 Hz), 50.29 (B', s), 44.88 (B, s).

EI HRMS: obtained m/z 349.12148 M^+ (expected m/z 349.12211 M^+).

MP: 79–80 °C.

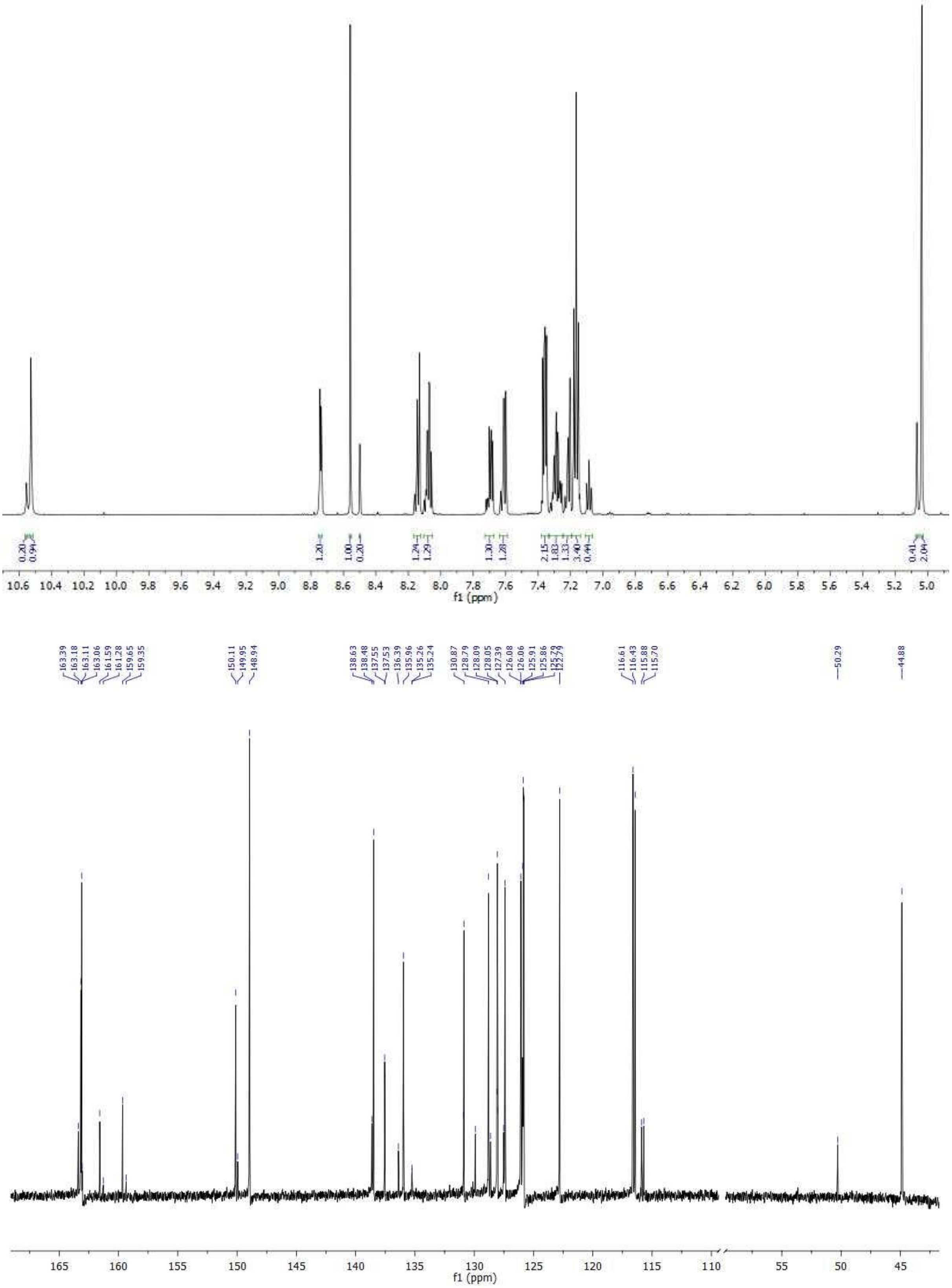

Figure S18 (Top) Partial ^{1}H NMR spectrum (500 MHz, 298 K, DMSO-d_6) and (Bottom) partial ^{13}C NMR spectrum (126 MHz, 298 K, DMSO-d_6) of compound **2**-C_1-Pyr.

5-fluoro-*N*-[2-[(4-fluoro-*N*-formyl-anilino)methyl]phenyl]pyridine-2-carboxamide **2**-C_1-PyrF

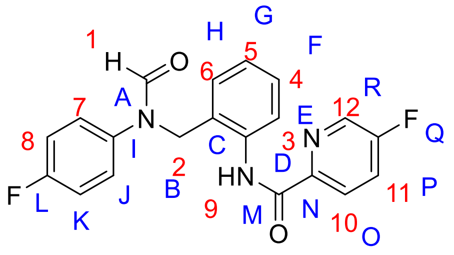

Prepared according to general propylphosphonic anhydride coupling procedure described above. DMF (4 mL), **1**-C_1-H (200 mg, 0.819 mmol), 5-fluoro-2-pyridinecarboxylic acid (110 mg, 0.819 mmol) propylphosphonic anhydride (50% mass) in ethyl acetate (540 μL, 0.900 mmol), triethylamine (100 mg, 140 μL, 0.983 mmol). Purification by flash column chromatography yielded a white solid (63.0 mg, 21%).

^{1}H NMR (601 MHz, DMSO-d_6) δ = 10.47 (9, s, 1H, 9', s, 1H), 8.74–8.73 (10, m, 1H, 10', m, 1H), 8.53 (1, s, 1H), 8.46 (1', s, 1H), 8.23–8.19 (12, m, 1H, 12', m 1H), 8.01–7.96 (11, m, 1H, 11', m, 1H), 7.55–7.52 (3, m, 1H, 3', m, 1H), 7.37–7.33 (7, m, 2H), 7.33–7.24 (4, m, 1H, 6, m, 1H, 7', m, 2H), 7.24–7.14 (8, m, 2H, 5, m, 1H, 5', m, 1H, 4', m, 1H, 6', m, 1H), 7.11–7.07 (8', 2H), 5.04 (2', s, 2H), 5.02 (2, s, 2H).

^{13}C NMR (126 MHz, DMSO-d_6) δ = 163.34 (s), 163.09 (s), 162.36 (s), 162.31 (s), 161.58 (s), 160.31 (s), 159.65 (s), 146.92 (d, *J* = 3.6 Hz), 146.78 (d, 2.5 Hz), 137.59 (d, *J* = 2.8 Hz), 137.42 (s), 137.22 (s), 136.25 (s), 135.89 (s), 135.33 (d, *J* = 3.8 Hz), 131.60 (s), 131.30 (s), 129.75 (s), 128.82 (s), 128.57 (s), 128.06 (s), 128.04 (s), 127.99 (s), 126.74 (s), 126.32 (s), 126.24 (s) 125.85 (d, J = 8.5 Hz), 125.33 (s), 125.13 (s), 125.07 (s), 125.04 (s), 116.51 (d, *J* = 22.6 Hz), 115.78 (d, *J* = 22.5 Hz), 50.27 (s), 44.93 (s).

^{19}F{^{1}H} NMR (471 MHz, DMSO-d_6) δ = −115.97 (minor), −116.41 (major), −122.26 (minor), −122.48 (major).

EI HRMS: obtained m/z 367.11162 M^+ (expected m/z 367.11268 M^+).

MP: 79–80 °C.

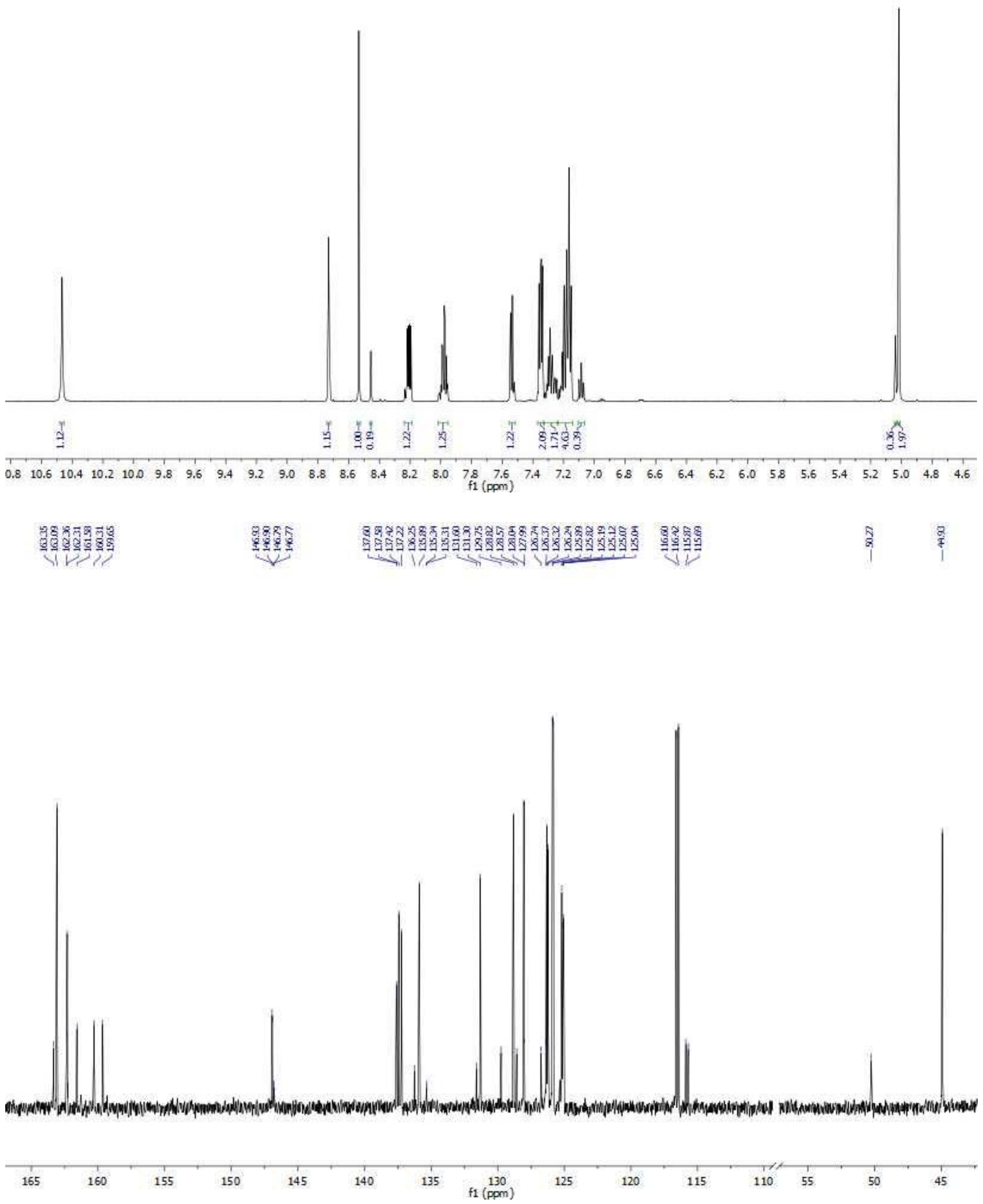

Figure S19 (Top) Partial ^{1}H NMR spectrum (500 MHz, 298 K, DMSO-d_6) and (Bottom) partial ^{13}C NMR spectrum (126 MHz, 298 K, DMSO-d_6) of compound **2**-C_1-PyrF.

S2.2.2 Ethylene (C_2) linker series

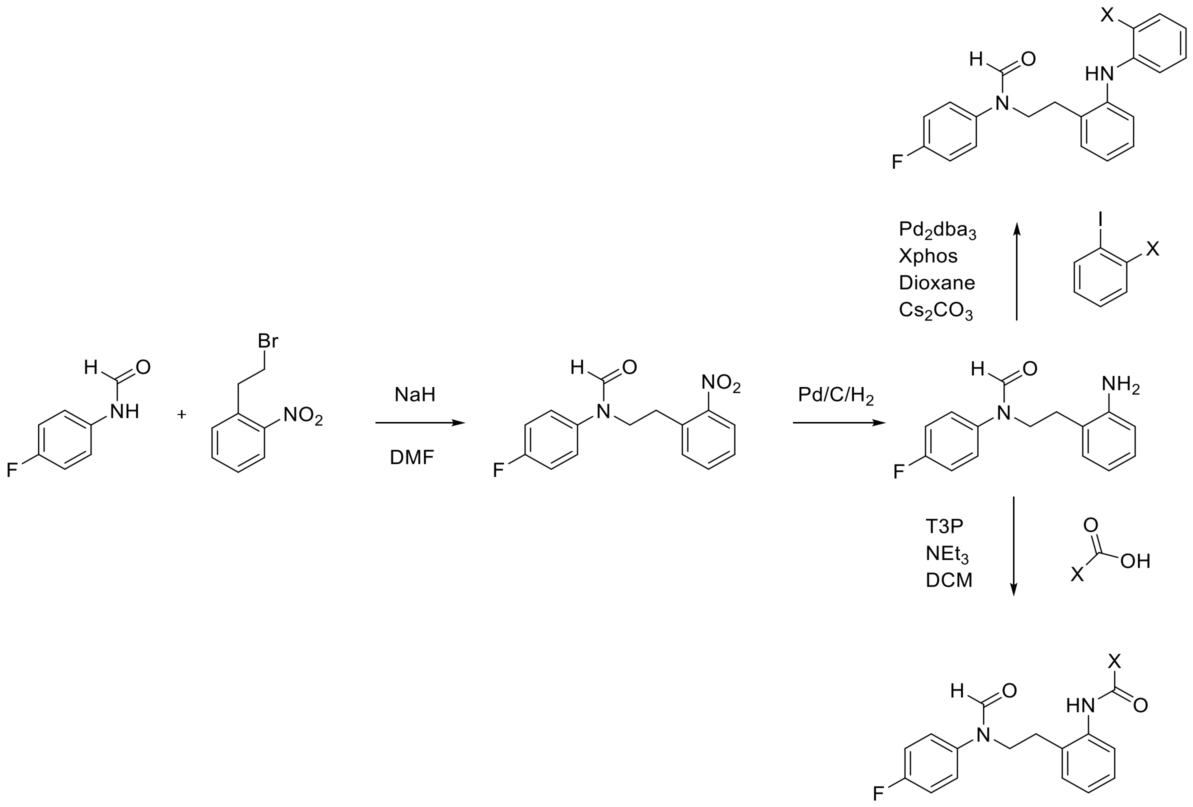

Figure S20 General scheme for the synthesis of ethylene linker balances **1**-C_2-X and **2**-C_2-Y.

N-(4-fluorophenyl)-*N*-[2-(2-nitrophenyl)ethyl]formamide **S4**

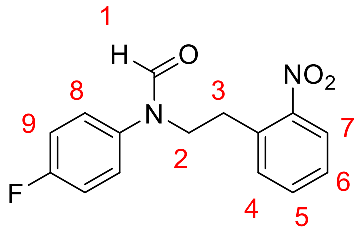

To a flask under a nitrogen atmosphere was added a solution of *N*-(4-fluorophenyl) formamide (200 mg, 1.44 mmol) in dry DMF (10 mL), 2-nitrophenethyl bromide (333 mg, 1.43 mmol) was then added and the mixture cooled to 0 °C. Sodium hydride (69.0 mg, 1.73 mmol) was then carefully added and the mixture allowed to warm to room temperature. The reaction mixture was stirred overnight, diluted in CH_2Cl_2 (10 mL) and quenched with water (10 mL). The organics were then dried with $MgSO_4$, solvent was removed under reduced pressure, and purified by flash column chromatography (*n*-Hex/EtOAc, 7:3, *v*/*v*) to yield a white solid (274 mg, 66%).

^{1}H NMR (500 MHz, $CDCl_3$) δ = 8.34 (1, s, 1H), 8.23 (1', s, 1H), 7.99 (7', m, 1H), 7.92 (7, m, 1H), 7.55 (5, 5', m, 2H), 7.44 (4, 4', m, 2H), 7.39 (6, m, 1H), 7.35–7.31 (6', m, 1H), 7.15–7.06 (8, 9, 8', 9', m, 8H), 4.14–4.09 (2, m, 2H), 4.06–4.01 (2', m, 2H), 3.21–3.16 (3, m, 2H), 3.13–3.08 (3', m, 2H).

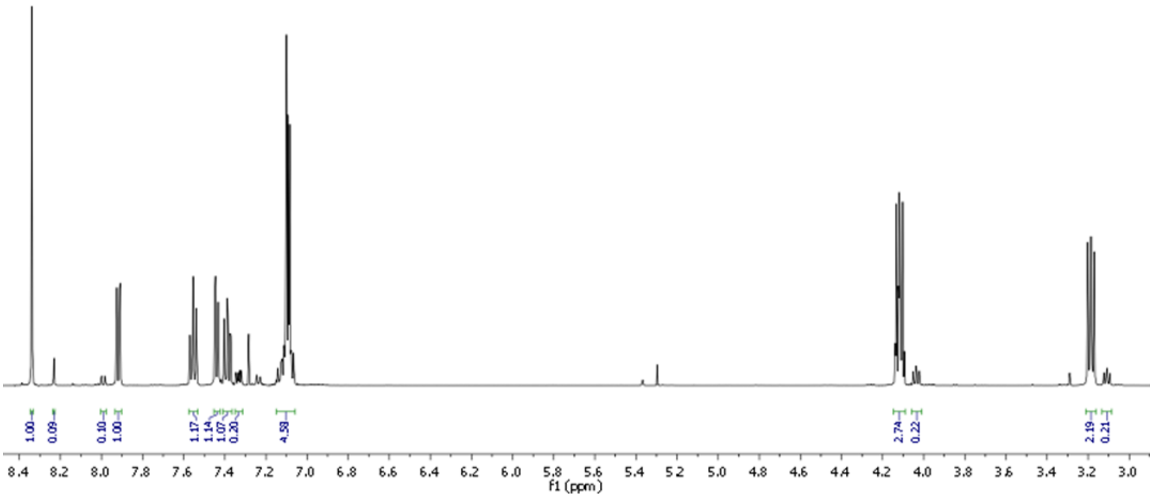

Figure S21 Partial ^{1}H NMR spectrum (500 MHz, 298 K, $CDCl_3$) of compound **S2**.

N-[2-(2-aminophenyl)ethyl]-*N*-(4-fluorophenyl)formamide **1**-C_2-H

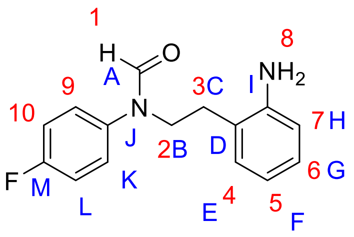

To a reaction flask under a nitrogen atmosphere was added a solution of **S4** (150 mg, 0.581 mmol) in EtOH (10 mL). Palladium on carbon (10 *wt* %) was then added and the reaction mixture placed under a hydrogen environment. The reaction mixture was monitored to completion via TLC and then filtered through Celite. The crude mixture was reduced under pressure and purified using column chromatography (SiO_2, *n*-Hex/EtOAc, 4:1, *v*/*v*) to yield a white solid (120 mg, 86%).

^{1}H NMR (601 MHz, DMSO-d_6) δ = 8.38 (1, s, 1H), 8.10 (1', s, 1H), 7.43–7.40 (9', m, 1H), 7.38–7.34 (9, m, 2H), 7.28–7.24 (10, m, 2H, 10', m, 2H), 6.94–6.90 (6, m, 1H, 6', m, 1H), 6.87 (4, dd, J = 7.4, 1.6 Hz, 1H), 6.77 (4', dd, J = 7.5, 1.6 Hz, 1H), 6.64–6.60 (7, m, 1H, 7', m, 1H), 6.49–6.45 (5, m, 1H, 5', m, 1H), 4.97 (8, s, 2H), 4.92 (8', s, 1H), 3.89–3.87 (2', m, 2H), 3.86–3.82 (2, m, 2H), 2.72–2.67 (3, m, 2H), 2.63–2.61 (3', m, 2H).

^{13}C NMR (151 MHz, DMSO-d_6) δ = 162.92 (A', s), 162.90 (A, s), 160.76 (M, d, *J* = 243.11 Hz), 160.39 (M', d, *J* = 243.11 Hz), 146.85 (I, s), 138.03 (J, d, *J* = 2.7 Hz), 135.60 (J', d, *J* = 2.8 Hz), 130.45 (E', s), 130.11 (E, s), 128.13 (K, d, J = 7.6 Hz), 127.76 (G', s), 127.71 (G, s), 126.13 (K', d, J = 8.5 Hz), 121.82 (D, s), 121.44 (D', s), 116.59 (L, d, *J* = 22.7 Hz), 116.65 (F, s), 116.65 (F', s), 116.07 (L', d, *J* = 22.5 Hz), 115.39 (H', s), 115.13 (H, s), 48.12 (B', s), 44.83 (B, s), 30.93 (C', s), 29.72 (C, s).

EI HRMS: obtained m/z 258.11552 M^+ (expected m/z 258.11629 M^+).

MP: 81–83 °C.

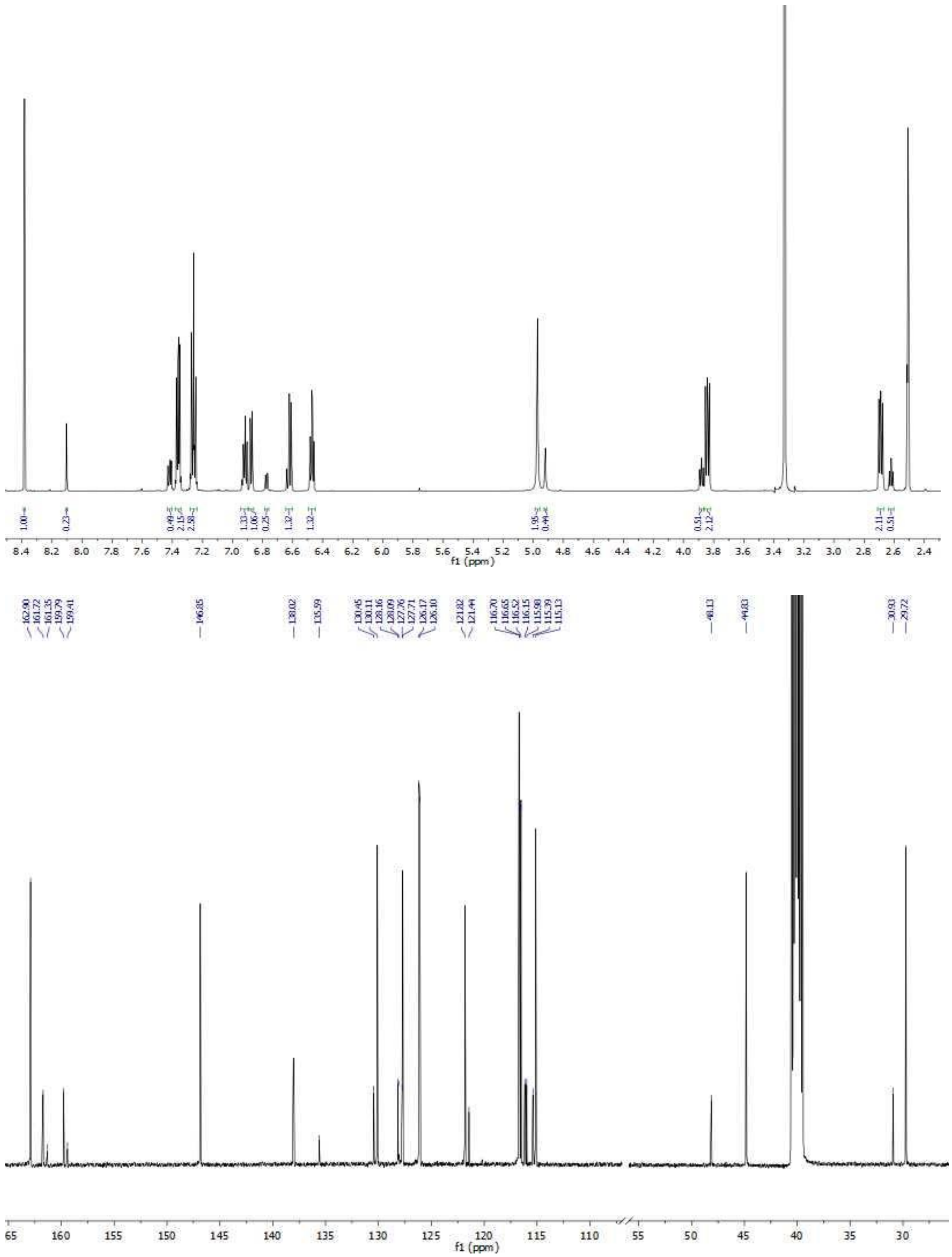

Figure S22 (Top) Partial ^{1}H NMR spectrum (500 MHz, 298 K, DMSO-d_6) and (Bottom) partial ^{13}C NMR spectrum (126 MHz, 298 K, DMSO-d_6) of compound **1**-C_2-H.

2-(4-nitrophenyl)acetaldehyde **S5**

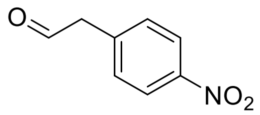

To a mixture of 2-(4-nitrophenyl)ethanol (1.00 g, 5.98 mmol) in MeCN (10 mL) was added iodoxybenzoic acid (8.4 g, 14.95 mmol). The reaction mixture was then refluxed for 2 hours. It was then filtered through Celite, washed with 1 M NaOH (10 mL) and extracted with CH_2Cl_2 (2 × 10 mL). The reaction was monitored to completion using TLC to yield an orange oil that was used used without further purification (612 mg, 62%).

4-fluoro-*N*-[2-(4-nitrophenyl)ethyl]aniline **S6**

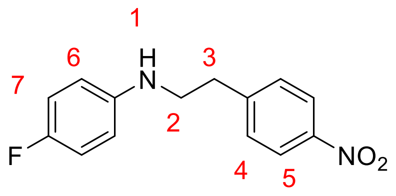

To a solution of 4-fluoroaniline (480 mg, 4.32 mmol) in dry methanol (10 mL) was added **S5** (720 mg, 4.36 mmol) and sodium cyanoborohydride (360 mg, 5.67 mmol). Acetic acid (1.31 g, 1.31 mL, 0.022 mmol) was then added dropwise and the mixture heated to reflux overnight. The reaction mixture was then reduced under pressure and dissolved in ethyl acetate (10 mL), washed with water (10 mL) and brine (10 mL). The reaction mixture was then concentrated, dried with $MgSO_4$ and purified by flash column chromatography (SiO_2, *n*-Hex/EtOAc, 7:3, *v*/*v*) to yield a yellow solid (866 mg, 77%).

^{1}H NMR (500 MHz, $CDCl_3$) δ = 8.23–8.18 (5, m, 2H), 7.42–7.37 (4, m, 2H), 6.96–6.89 (7, m, 2H), 6.60–6.54 (6, m, 2H), 3.55 (1, s, 1H), 3.45 (2, t, J = 6.9 Hz, 1H), 3.04 (3, t, J = 6.9 Hz, 1H).

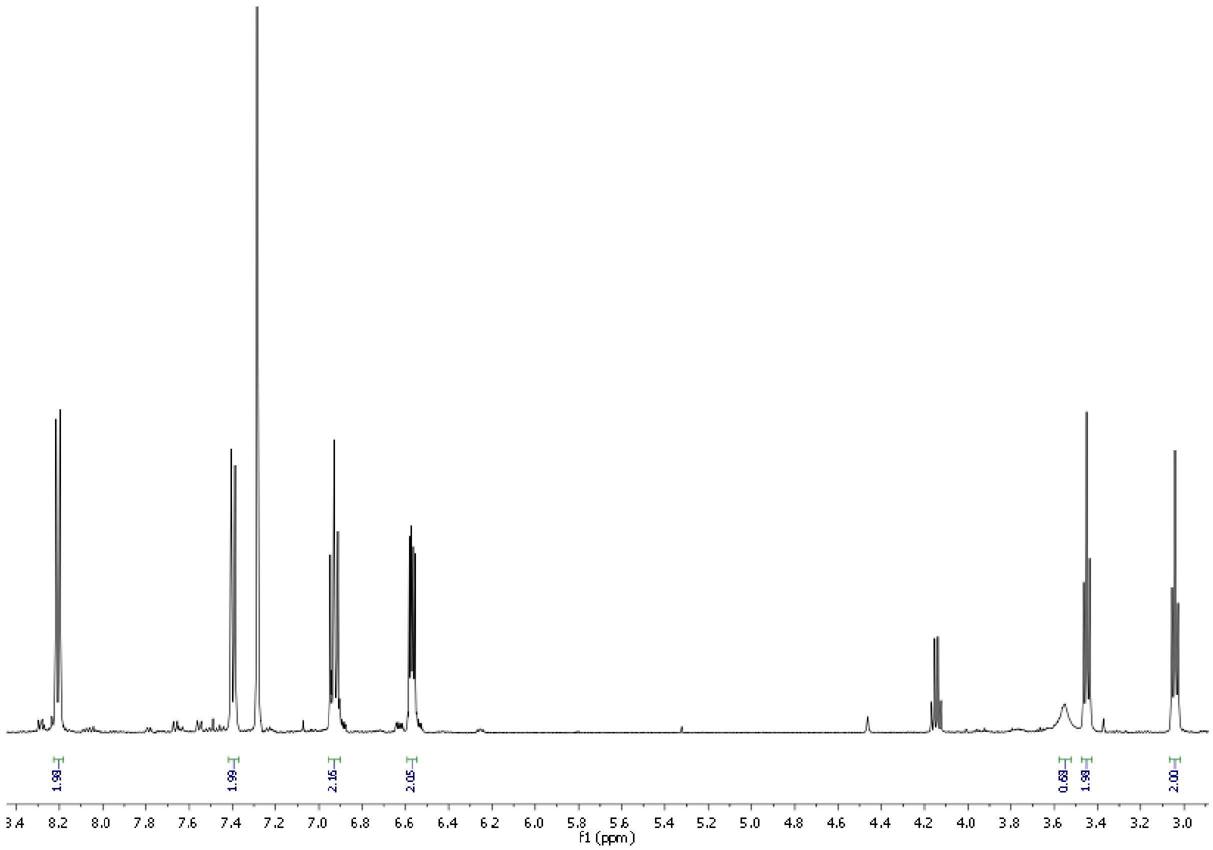

Figure S23 Partial ^{1}H NMR spectrum (500 MHz, 298 K, $CDCl_3$) of compound **S6**.

N-(4-fluorophenyl)-*N*-[2-(4-nitrophenyl)ethyl]formamide **S7**

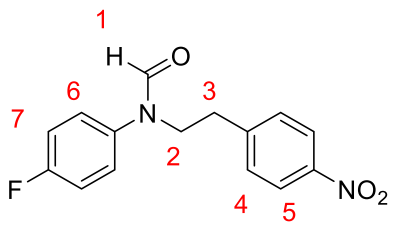

To a solution of **S6** (300 mg, 1.15 mmol) in dry CH_2Cl_2 (10 mL) was added formic acid (53 mg, 43 μL, 1.15 mmol). Propylphosphonic anhydride solution (808 mg, 755 μL, 1.27 mmol) was then added followed by trimethylamine (140 mg, 0.193 μL, 1.38 mmol). The reaction mixture was stirred overnight at room temperature and then extracted with CH_2Cl_2 (10 mL), washed with 2 M HCl (10 mL), water (10 mL) and brine (10 mL). The combined organic phases were then dried over $MgSO_4$ and concentrated in *vacuo*. The crude product was then purified by flash column chromatography (SiO_2, *n*-Hex/EtOAc, 1:1, *v*/*v*) to yield a yellow solid (288 mg, 87%).

^1H NMR (400 MHz, $CDCl_3$) δ = 8.30 (1, s, 1H), 8.18–8.14 (5, m, 2H), 7.38–7.33 (4, m, 2H), 7.16–7.05 (6, 7, m, 4H), 4.13–4.06 (2, m, 2H), 3.03–2.97 (3, m, 2H).

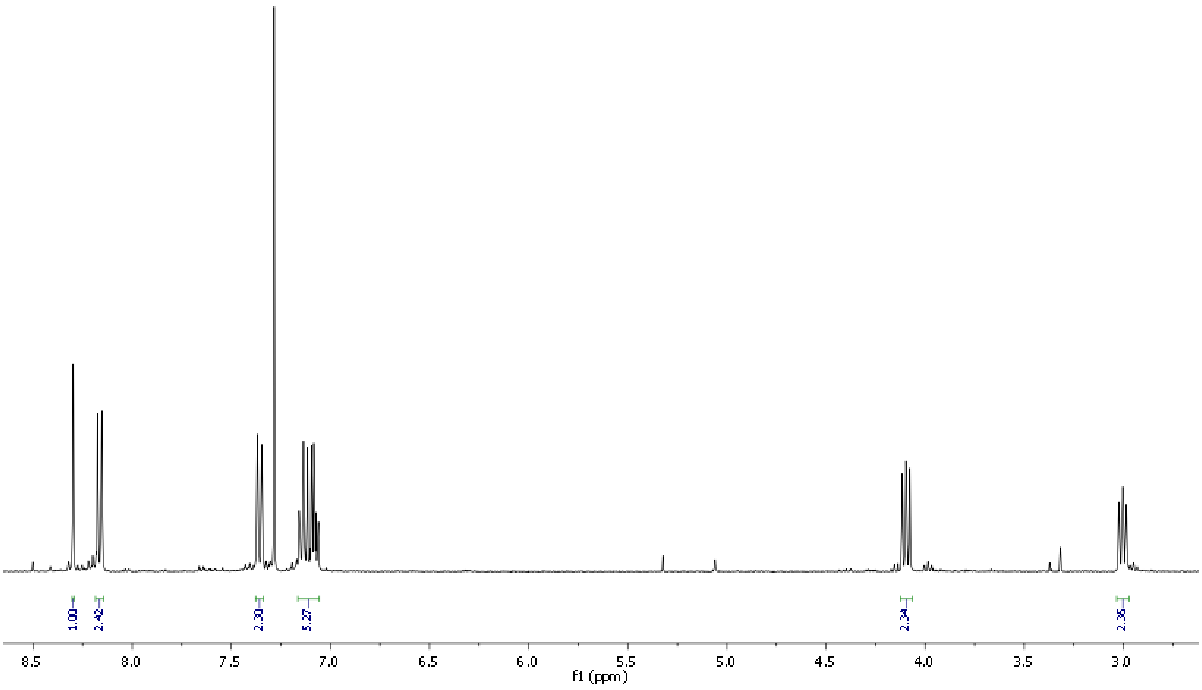

Figure S24 Partial ^1H NMR spectrum (500 MHz, 298 K, $CDCl_3$) of compound **S7**.

N-[2-(4-aminophenyl)ethyl]-*N*-(4-fluorophenyl)formamide **Control**-C_2

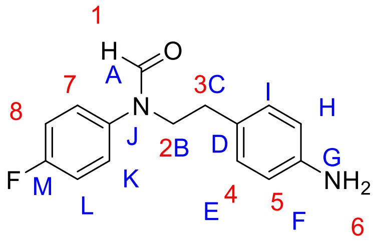

To a reaction flask under a nitrogen atmosphere was added a solution of **S7** (200 mg, 0.694 mmol) in EtOH (10 mL). Palladium on carbon (10% wt) was then added and the reaction mixture placed under a hydrogen environment. The reaction mixture was monitored to completion via TLC and then filtered through Celite. The crude mixture was reduced under pressure and purified by flash column chromatography (SiO_2, *n*-Hex/EtOAc, 4:1, *v*/*v*) to yield a white solid (132 mg, 74%).

^{1}H NMR (500 MHz, DMSO-d_6) δ = 8.32 (1, s, 1H), 8.07 (1', s, 1H), 7.40–7.37 (7', m, 2H), 7.36–7.31 (7, m, 2H), 7.29–7.24 (8', 8, m, 4H), 6.82–6.79 (4, 4', m, 4H), 6.52–6.48 (5', m, 2H), 6.48–6.44 (5, m, 2H), 4.90 (6', s, 2H), 4.87 (6, s, 2H), 3.91–3.83 (2, 2', m, 4H), 2.59–2.53 (3, 3', m, 4H).

^{13}C NMR (126 MHz, DMSO-d_6) δ = 162.85 (s), 162.42 (s), 160.72 (d, *J* = 243.2 Hz), 160.41 (d, *J* = 244.4 Hz), 147.50 (s), 147.42 (s), 137.72 (d, *J* = 2.6 Hz), 135.43 (d, *J* = 2.9 Hz), 129.71 (s), 129.48 (s), 128.21 (d, *J* = 8.5 Hz), 126.30 (d, *J* = 8.5 Hz), 125.62 (s), 125.11 (s), 116.63 (d, *J* = 22.5 Hz), 116.10 (d, *J* = 22.4 Hz), 114.48 (s), 114.44 (s), 51.05 (s), 46.46 (s), 33.98 (s), 32.80 (s).

^{19}F{^{1}H} NMR (471 MHz, DMSO-d_6) δ = −115.90 to −115.96 (minor, m), −116.39 to −116.44 (major, m).

EI HRMS: obtained m/z 258.11722 M^+ (expected m/z 258.11629 M^+).

MP: 84–86 °C.

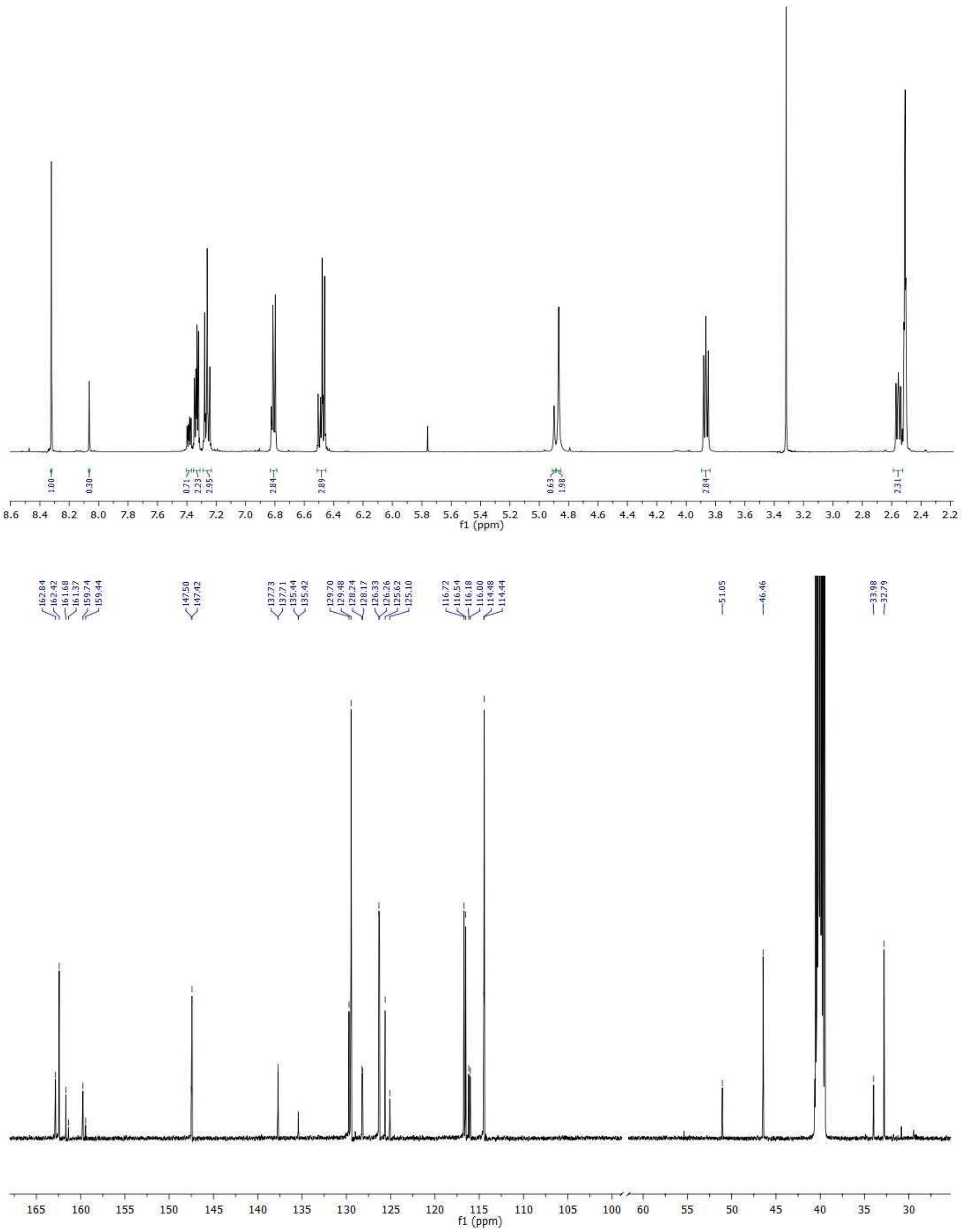

Figure S25 (Top) Partial ^{1}H NMR spectrum (500 MHz, 298 K, DMSO-d_6) and (Bottom) partial ^{13}C NMR spectrum (126 MHz, 298 K, DMSO-d_6) of compound **Control**-C_2.

General Buchwald-Hartwig coupling

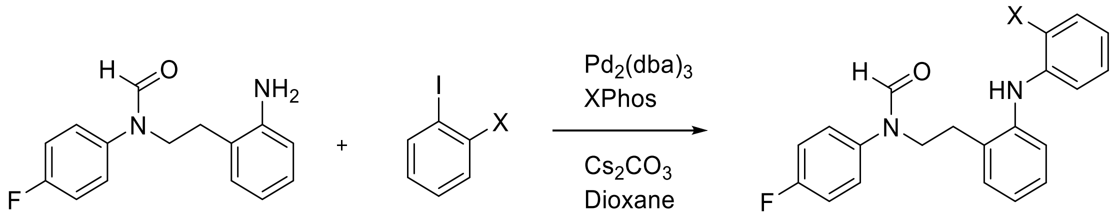

Figure S26 General scheme for Buchwald-Hartwig couplings.

To a flask under a nitrogen atmosphere was added a solution of **1**-C_2-H in degassed dioxane (10 mL). The appropriately substituted iodobenzene was then added along with caesium carbonate, XPhos and $Pd_2(dba)_3$. The reaction mixture was then refluxed overnight and filtered through celite. Water was added and then the reaction mixture was extracted with CH_2Cl_2 (2 × 10 mL), dried over $MgSO_4$, concentrated under reduced pressure, and purified by flash column chromatography.

N-[2-(2-anilinophenyl)ethyl]-*N*-(4-fluorophenyl)formamide **1**-C_2-Ph

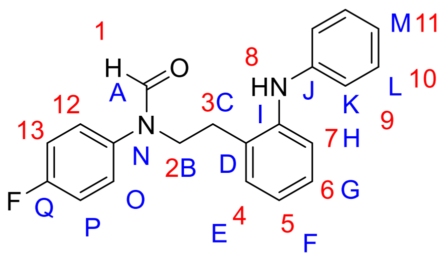

Prepared according to the general Buchwald-Hartwig coupling procedure described above. Dioxane (10 mL), **1**-C_2t-H (300 mg, 1.16 mmol), iodobenzene (400 mg, 224 µL, 1.97 mmol), Cs_2CO_3 (1.14 g, 3.48 mmol), XPhos (55.3 mg, 0.116 mmol) and $Pd_2(dba)_3$ (63.8 mg, 0.0697 mmol). Purification by flash column chromatography (SiO_2, *n*-Hex/EtOAc, 1:1, *v*/*v*) yielded a yellow solid (91 mg, 23%).

^{1}H NMR (500 MHz, DMSO-d_6) δ = 8.36 (1, s, 1H), 8.08 (1', s, 1H), 7.47 (8, s, 1H), 7.45 (8', s, 1H), 7.35–7.30 (12', m, 2H), 7.27–7.22 (12, m, 2H), 7.21–7.13 (13, m, 2H, 13', m, 2H, 4, m, 1H, 4', m, 1H, 6, m, 1H, 6', m, 1H, 5, m, 1H, 5', m, 1H), 6.99 (7', m, 1H), 6.95 (7, m, 1H), 6.84–6.70 (9, m, 2H, 9', m, 2H, 11, m, 1H, 11', m, 1H, 10, m, 1H, 10', m, 1H), 3.91–3.88 (2, 2', m, 4H), 2.88–2.81 (3, m, 2H), 2.79–2.76 (3', m, 2H).

^{13}C NMR (126 MHz, DMSO-d_6) δ = 162.75 (s), 160.64 (d, *J* = 243.2 Hz), 160.30 (d, *J* = 243.2 Hz), 146.03 (s), 145.72 (s), 141.63 (s), 141.58 (s), 137.85 (d, *J* = 2.8 Hz), 135.41 (d, *J* = 3.1 Hz), 131.42 (s), 131.39 (s), 131.17 (s), 131.00 (s), 129.54 (s), 129.48 (s), 127.99 (s), 127.89 (s), 127.83 (s), 125.82 (d, *J* = 8.5 Hz), 123.33 (s), 122.86 (s), 122.72 (s), 121.50 (s), 119.20 (s), 119.04 (s), 116.53 (d, *J* = 22.5 Hz), 116.13 (s), 115.96 (d, *J* = 22.4 Hz), 115.82 (s), 49.07 (s), 45.18 (s), 31.36 (s), 29.88 (s).

^{19}F{^{1}H} NMR (471 MHz, DMSO-d_6) δ = −116.09 (minor), −116.58 (major).

EI HRMS: obtained m/z 334.14816 M^+ (expected m/z 334.14759 M^+)

MP: 86–87 °C.

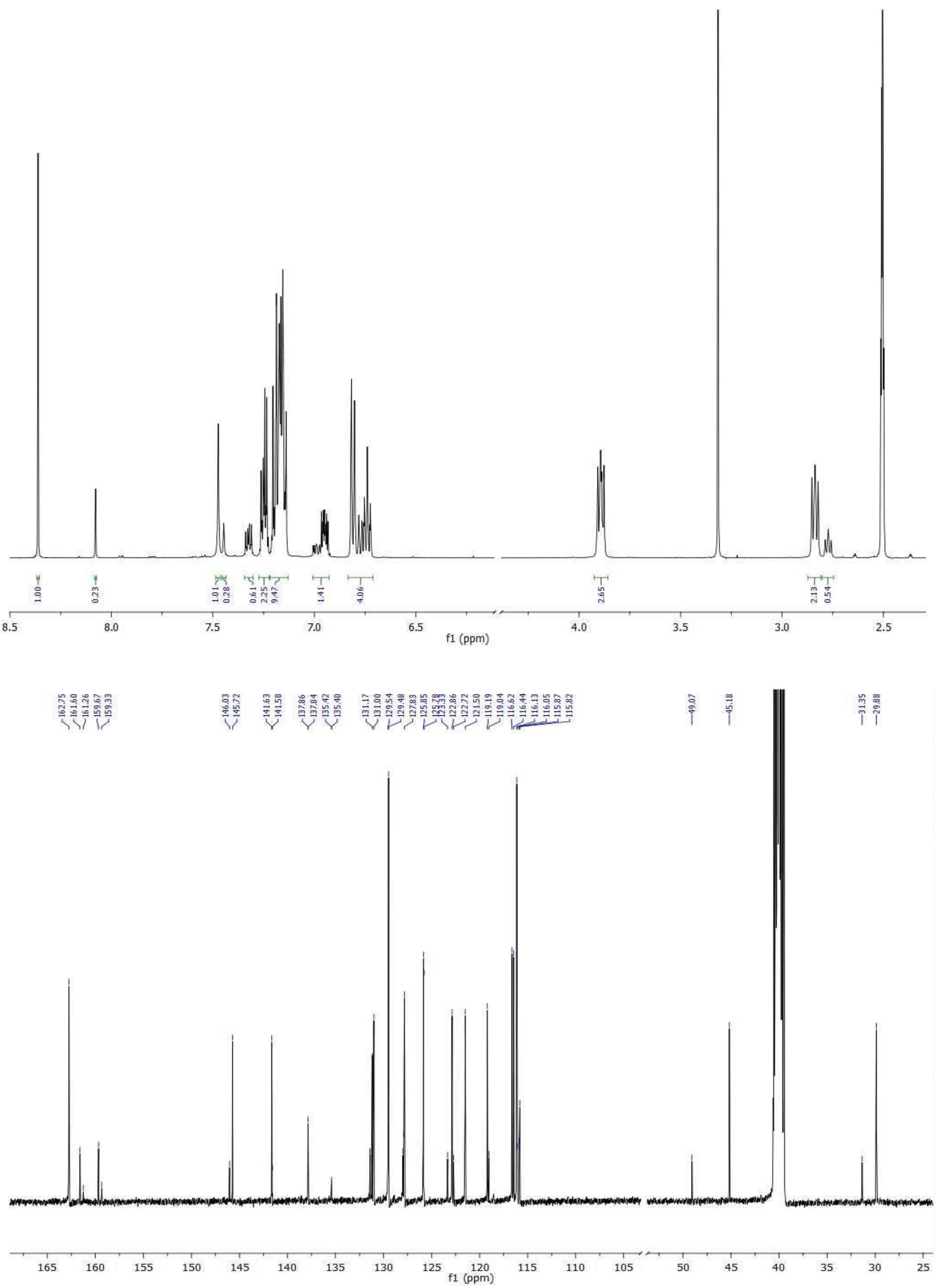

Figure S27 (Top) Partial ^{1}H NMR spectrum (500 MHz, 298 K, DMSO-d_6) and (Bottom) partial ^{13}C NMR spectrum (126 MHz, 298 K, DMSO-d_6) of compound **1**-C_2-Ph.

N-(4-fluorophenyl)-N-[2-[2-(2-methoxyanilino)phenyl]ethyl]formamide **1**-C_2-PhOMe

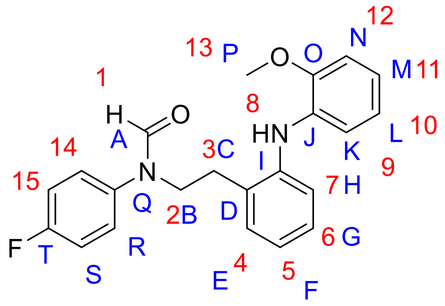

Prepared according to the general Buchwald-Hartwig coupling procedure described above. Dioxane (10 mL), **1**-C_2-H (300 mg, 1.16 mmol), 2-iodoanisole (462 mg, 257 μL, 1.97 mmol), Cs_2CO_3 (1.14 g, 3.48 mmol), XPhos (55.3 mg, 0.116 mmol) and $Pd_2(dba)_3$ (63.8 mg, 0.0697 mmol). Purification by flash column chromatography (SiO_2, n-Hex/EtOAc, 1:1, v/v) yielded a brown oil (110 mg, 26%).

^{1}H NMR (500 MHz, DMSO-d_6) δ = 8.35 (1, s, 1H), 8.09 (1', s, 1H), 7.35–7.30 (14', m, 1H), 7.27–7.21 (14, m, 2H, 15', m, 2H), 7.21–7.13 (15, m, 2H, 5, m, 1H, 5', m, 1H), 7.04–7.00 (7, m, 1H, 7', m, 1H), 7.00–6.94 (5, m, 1H, 5', m, 1H, 6, m, 1H, 6', m, 1H), 6.81–6.72 (9, m, 1H, 9', m, 1H, 11, m, 1H, 11', m, 1H), 6.66–6.58 (12, m, 1H, 12', m, 1H, 10, m, 1H, 10', m, 1H), 3.93–3.87 (2, m, 2H, 2', m, 2H), 3.81 (13, s, 3H, 13', s, 3H), 2.83–2.77 (3, m, 2H), 2.74 (3', m, 2H).

^{13}C NMR (126 MHz, DMSO-d_6) δ = 162.82 (s), 162.60 (s), 160.61 (d, J = 242.9 Hz), 149.03 (s), 148.93 (s), 141.77 (s), 137.76 (d, J = 2.6 Hz), 135.38 (d, J = 3.0 Hz), 134.91 (s), 134.67 (s), 131.46 (s), 131.33 (s), 131.16 (s), 130.85 (s), 127.98 (s), 127.84 (s), 127.76 (s), 125.77 (d, J = 8.5 Hz), 123.31 (s), 123.08 (s), 123.00 (s), 122.01 (s), 121.17 (s), 121.10 (s), 120.03 (s), 119.94 (s), 116.51 (d, J = 22.5 Hz), 115.95 (d, J = 22.4 Hz), 115.46 (s), 115.28 (s), 111.59 (s), 111.54 (s), 55.99 (s), 55.95 (s), 48.95 (s), 45.03 (s), 31.32 (s), 29.78 (s).

^{19}F{^{1}H} NMR (471 MHz, DMSO-d_6) δ = −116.11 (minor), −116.60 (major).

EI HRMS: obtained m/z 364.15891 M^+ (expected m/z 364.15816 M^+).

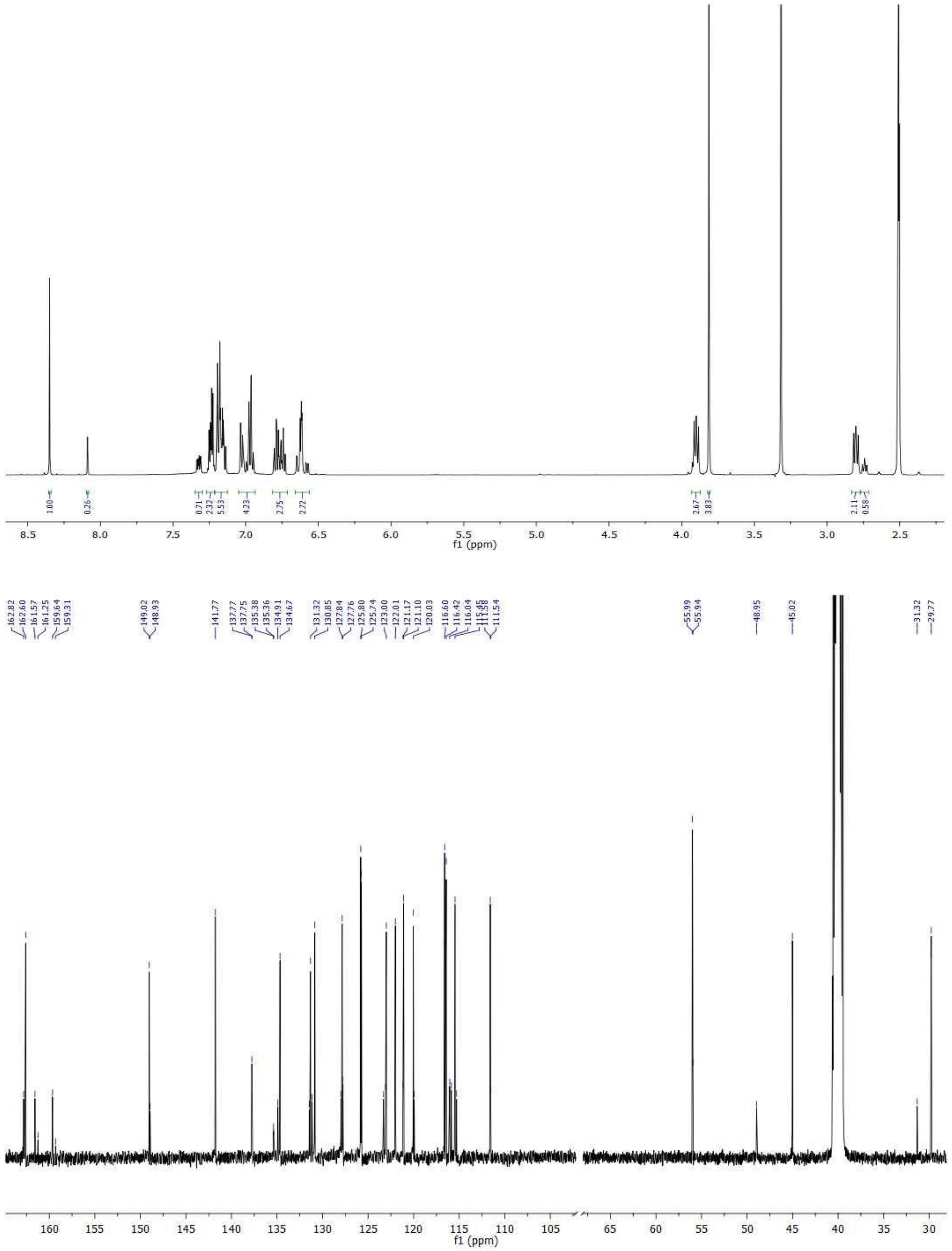

Figure S28 (Top) Partial ^{1}H NMR spectrum (500 MHz, 298 K, DMSO-d_6) and (Bottom) partial ^{13}C NMR spectrum (126 MHz, 298 K, DMSO-d_6) of compound **1**-C_2-PhOMe.

N-[2-[2-(2-cyanoanilino)phenyl]ethyl]-*N*-(4-fluorophenyl)formamide **1**-C_2-PhCN

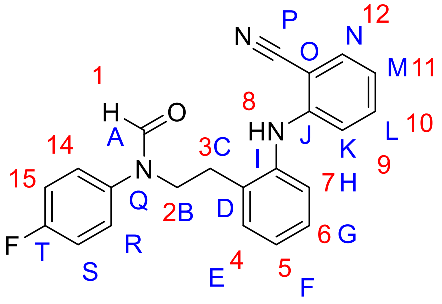

Prepared according to the general Buchwald-Hartwig coupling procedure described above. Dioxane (10 mL), **1**-C_2-H (300 mg, 1.16 mmol), 2-iodobenzonitrile (453 mg, 1.98 mmol), Cs_2CO_3 (1.14 g, 3.48 mmol), XPhos (55.3 mg, 0.116 mmol) and $Pd_2(dba)_3$ (63.8 mg, 0.0697 mmol). Purification by flash column chromatography (SiO_2, *n*-Hex/EtOAc, 1:1, *v*/*v*) yielded a yellow solid (337 mg, 81%).

^{1}H NMR (601 MHz, DMSO-*d*$_6$) δ = 8.32 (1, s, 1H), 8.14 (1', s, 1H), 7.96 (8', s, 1H), 7.90 (8, s, 1H), 7.60–7.54 (12, m, 1H, 12', m, 1H), 7.38–7.09 (15, m, 2H, 15', m, 2H, 14, m, 2H, 14', m, 2H, 6, m, 1H, 6', m, 1H, 5, m, 1H, 5', m, 1H, 4, m, 1H, 11, m, 1H, 11', m, 1H, 10, m, 1H, 10', m, 1H, 9, m, 1H, 9', m, 1H), 6.85 – 6.80 (5, m, 1H, 5', m, 1H), 6.47–6.44 (7, m, 1H, 7', m, 1H), 3.92–3.86 (2, m, 2H, 2', m, 2H), 2.76-2.73 (3, m, 2H), 2.70–2.67 (3', m, 2H).

^{13}C NMR (126 MHz, DMSO-*d*$_6$) δ = 162.81 (s), 162.49 (s), 160.60 (d, *J* = 243.0 Hz), 149.60 (s), 149.54 (s), 139.84 (s), 139.79 (s), 137.53 (d, *J* = 2.9 Hz), 135.23 (s), 134.72 (s), 134.70 (s), 134.60 (s), 134.24 (s), 134.18 (s), 134.13 (s), 131.45 (s), 131.11 (s), 128.45 (s), 128.30 (s), 127.79 (d, *J* = 8.4 Hz), 126.48 (s), 126.36 (s), 126.07 (s), 126.02 (s), 125.71 (d, *J* = 8.5 Hz), 119.12 (s), 119.00 (s), 118.22 (s), 118.18 (s), 116.58 (d, *J* = 22.5 Hz), 116.04 (d, *J* = 22.5 Hz), 115.68 (s), 115.55 (s), 98.04 (s), 97.99 (s), 49.26 (s), 44.87 (s), 31.27 (s), 29.75 (s).

EI HRMS: obtained m/z 359.14277 M^+ (expected m/z 359.14284 M^+).

MP: 102–104 °C.

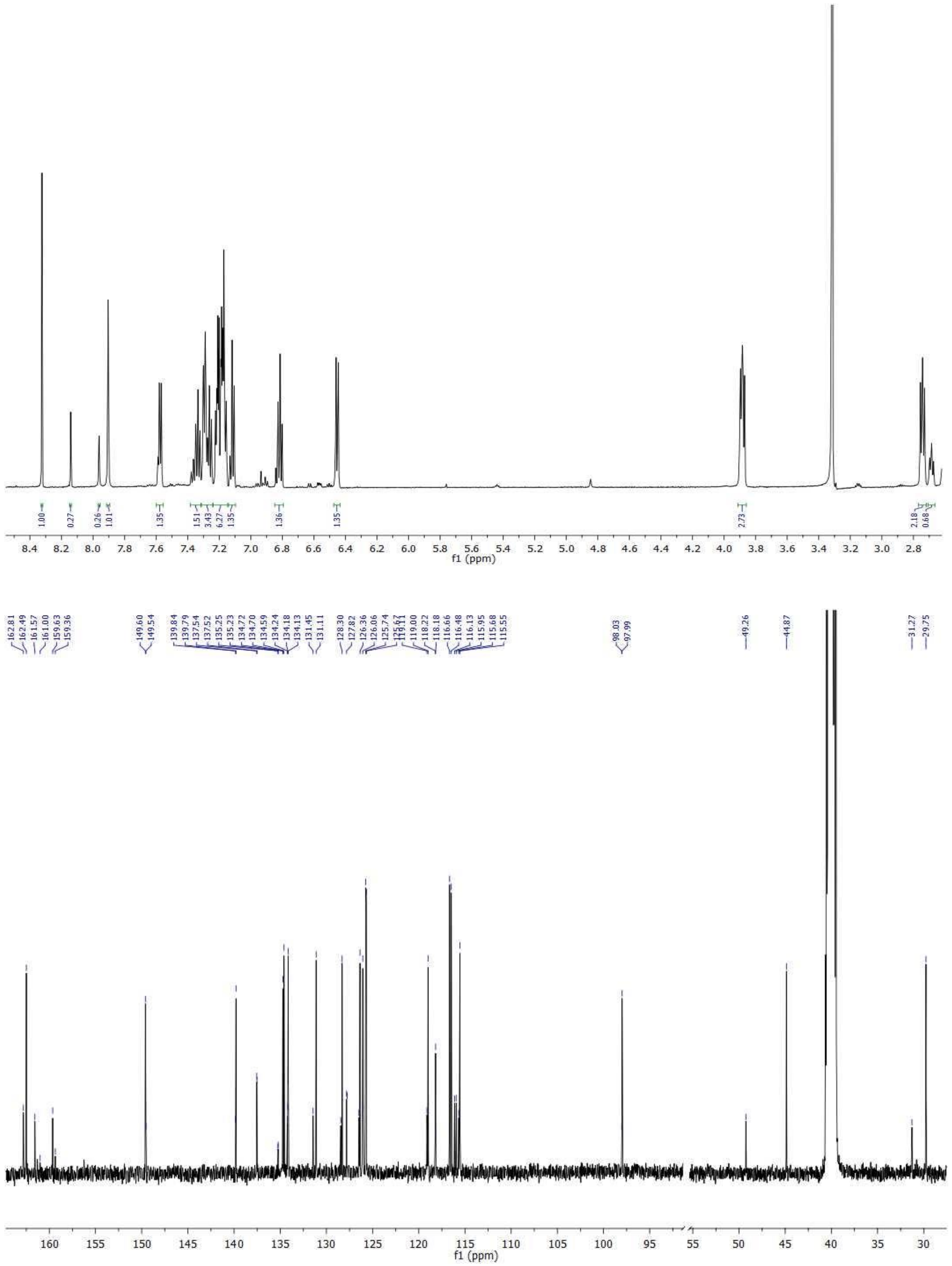

Figure S29 (Top) Partial ^{1}H NMR spectrum (500 MHz, 298 K, DMSO-d_6) and (Bottom) partial ^{13}C NMR spectrum (126 MHz, 298 K, DMSO-d_6) of compound **1**-C_2-PhCN.

General amide coupling

General propyl phosphonic anhydride coupling

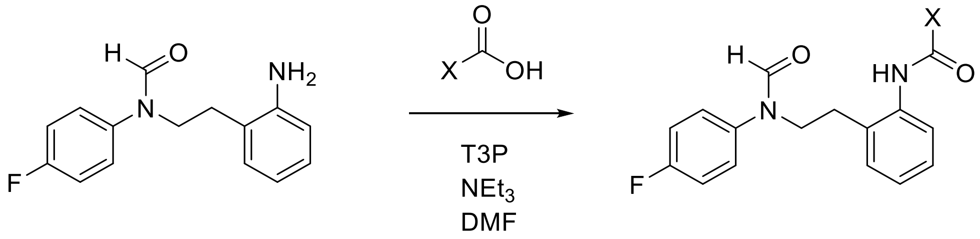

Figure S30 General procedure for propylphosphonic anhydride coupling.

To a solution of **1**-C_2-H in dry DMF was added an appropriately substituted acid. Propylphosphonic anhydride (T3P) solution was then added followed by triethylamine. The reaction mixture was stirred overnight at room temperature and then extracted with CH_2Cl_2, washed with 2 M HCl, water and brine. The combined organic phases were then dried over $MgSO_4$ and concentrated *in vacuo*. The crude product was then purified by flash column chromatography.

N-[2-[2-(4-fluoro-*N*-formyl-anilino)ethyl]phenyl]acetamide **2**-C_2-Me

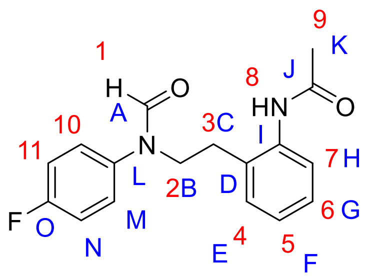

Prepared according to general propylphosphonic anhydride coupling procedure described above. DMF (4 mL), **1**-C_2-H (200 mg, 0.774 mmol), acetic acid (46.5 mg, 44.3 µL, 0.774 mmol), propylphosphonic anhydride (50%) in ethyl acetate (542 mg, 507 µL, 0.852 mmol), triethylamine (94.0 mg, 130 µL, 0.929 mmol). Purification by flash column chromatography (SiO_2, *n*-Hex/EtOAc, 1:1, *v*/*v*) yielded a white solid (160 mg, 69%).

^{1}H NMR (601 MHz, DMSO-*d*$_6$) δ = 9.35 (8, s, 1H), 8.37 (1, s, 1H), 8.14 (1', s, 1H), 7.41–7.34 (10, m, 2H, 10', m, 2H, 7, m, 1H, 7', m, 1H), 7.30–7.25 (11, m, 2H, 11', m, 2H), 7.23–7.18 (4, m, 1H, 4', m, 1H, 6, m, 1H, 6', m, 1H), 7.17–7.09 (5, m, 1H, 5', m, 1H), 3.89–3.80 (2, m, 2H, 2', m, 2H), 2.81–2.76 (3, m, 2H), 2.72 (3', t, J = 7.5 Hz, 2H), 2.01 (9, s, 3H), 1.96 (9, s, 3H).

^{13}C NMR (126 MHz, DMSO-*d*$_6$) δ = 169.07 (J, s), 162.84 (A', s), 162.70 (A, s), 160.79 (O, d, *J* = 243.18 Hz), 160.45 (O', d, *J* = 243.18 Hz), 137.76 (L, d, J = 2.5 Hz), 136.94 (I, s), 136.78 (I', s), 135.35 (L', d, *J* = 2.9 Hz), 133.44 (D', s), 133.11 (D, s), 130.67 (E', s), 130.39 (E, s), 128.10 (M', d, *J* = 8.5 Hz), 127.39 (G', s), 127.31 (G, s), 126.48 (H, s), 126.25 (F', s), 126.15 (M, d, *J* = 8.5 Hz), 125.96 (F, s), 116.65 (N, d, *J* = 22.6 Hz), 116.10 (N', d, *J* = 22.5 Hz), 49.40 (B', s), 45.52 (B, s), 31.27 (C', s), 29.86 (C, s), 23.62 (K, s), 23.48 (K', s).

^{19}F NMR (471 MHz, DMSO-*d*$_6$) δ = −115.95 (minor), −116.36 (major).

EI HRMS: obtained m/z 300.12757 M^+ (expected m/z 300.12686 M^+).

MP: 110–113 °C

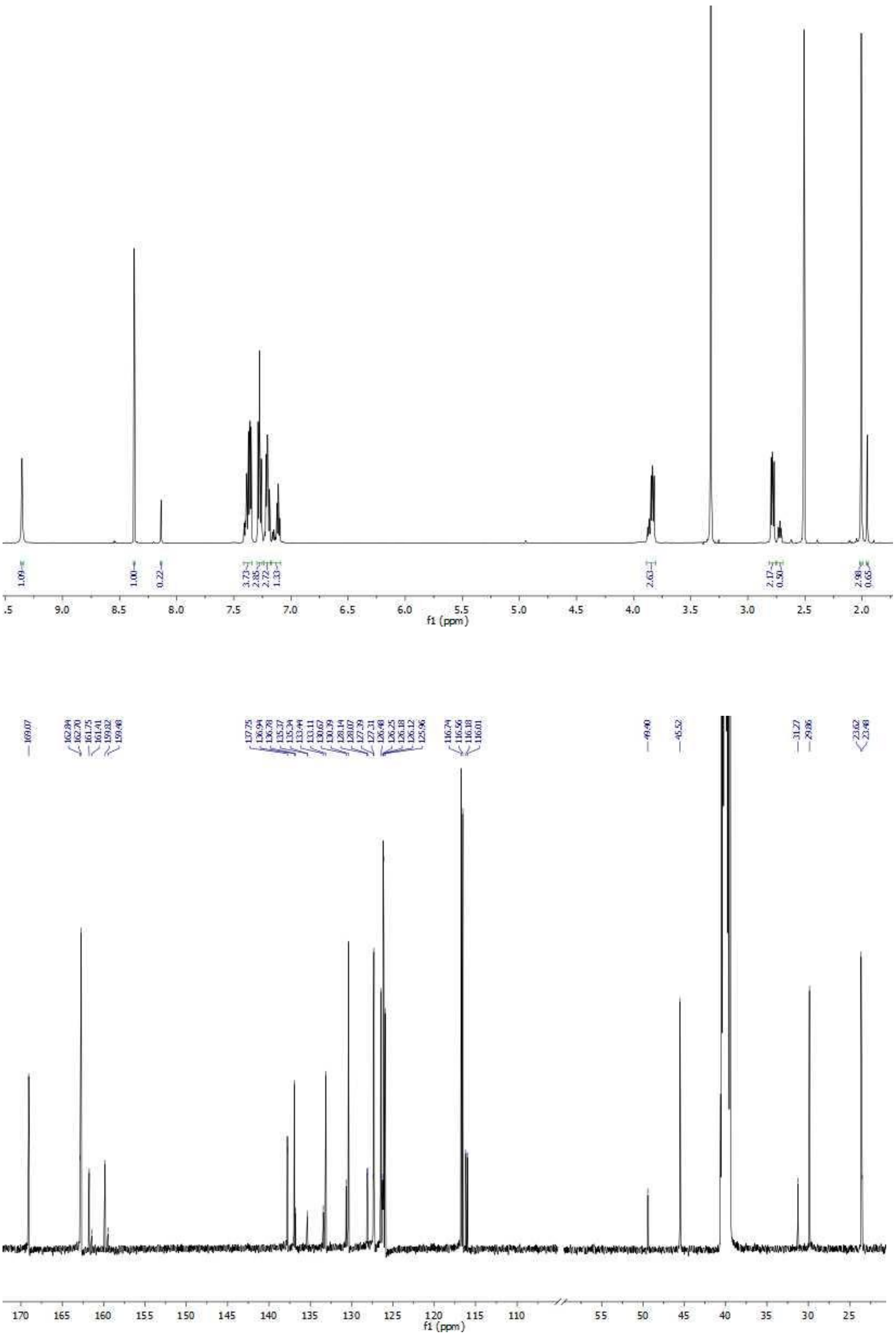

Figure S31 (Top) Partial ^{1}H NMR spectrum (500 MHz, 298 K, DMSO-d_6) and (Bottom) partial ^{13}C NMR spectrum (126 MHz, 298 K, DMSO-d_6) of compound **2**-C_2-Me.

2,2,2-trifluoro-*N*-[2-[2-(4-fluoro-*N*-formyl-anilino)ethyl]phenyl]acetamide **2**-C_2-CF_3

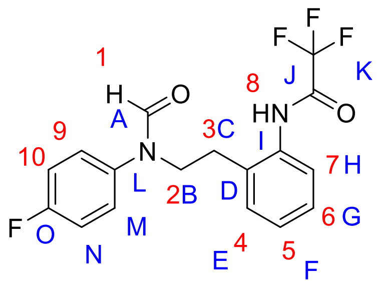

Prepared according to general propylphosphonic anhydride coupling procedure described above. DMF (4 mL), **1**-C_2-H (200 mg, 0.774 mmol), trifluoroacetic acid (88.3 mg, 59.3 µL, 0.774 mmol), propylphosphonic anhydride (50%) in ethyl acetate (542 mg, 507 µL, 0.852 mmol), triethylamine (94.0 mg, 130 µL, 0.929 mmol). Purification by flash column chromatography (SiO_2, *n*-Hex/EtOAc, 1:1, *v*/*v*) yielded a white solid (195 mg, 71%).

^{1}H NMR (601 MHz, DMSO-d_6) δ = 10.93 (8, s, 1H), 8.32 (1, s, 1H), 8.16 (1’, s, 1H), 7.38–7.29 (9, m, 2H, 9’, m, 2H, 7, m, 1H, 4, m, 1H, 7’, m, 1H, 6, m, 1H, 6’, m, 1H), 7.29–7.22 (10, m, 2H, 10’, m, 2H, 5, m, 1H, 5’, m, 1H), 3.90–3.83 (2, m, 2H, 2’, m, 2H), 2.76–2.71 (3, m, 2H), 2.70–2.65 (3’, m, 2H).

^{13}C NMR (126 MHz, DMSO-d_6) δ = 162.77 (s), 162.48 (s), 160.82 (d, *J* = 243.3 Hz), 160.57 (d, *J* = 243.18 Hz), 156.20 (q, *J* = 36.6 Hz), 137.56 (d, *J* = 2.9 Hz), 135.34 (s), 135.13 (d, *J* = 3.0 Hz), 134.87 (s), 133.83 (s), 131.01 (s), 130.74 (s), 128.44 (s), 128.23 (d, *J* = 8.5 Hz), 128.07 (s), 127.96 (s), 127.83 (s), 126.34 (d, *J* = 8.5 Hz), 119.92 (s), 117.63 (s), 116.61 (d, *J* = 22.6 Hz), 116.11 (d, *J* = 22.5 Hz), 115.33 (s), 113.04 (s), 49.39 (s), 45.30 (s), 30.93 (s), 29.42 (s).

^{19}F{^{1}H} NMR (471 MHz, DMSO-d_6) δ = −73.95 (C*F*$_3$, major), −74.00 (C*F*$_3$, minor), −115.89 (C*F*, minor), −116.35 (C*F*, major).

EI HRMS: obtained m/z 354.09803 M^+ (expected m/z 354.09859 M^+).

MP: 125–127 °C.

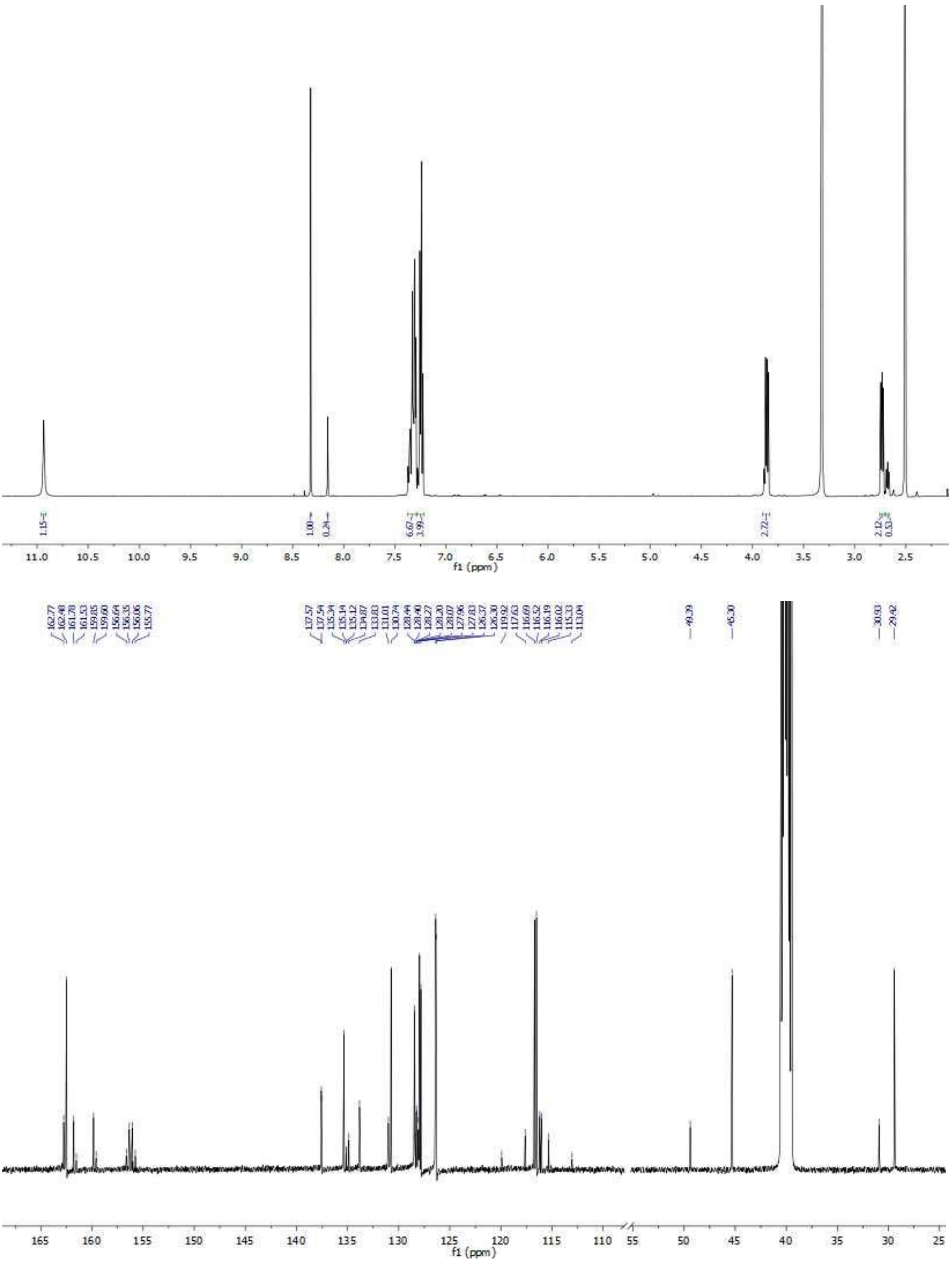

Figure S32 (Top) Partial ^{1}H NMR spectrum (500 MHz, 298 K, DMSO-d_6) and (Bottom) partial ^{13}C NMR spectrum (126 MHz, 298 K, DMSO-d_6) of compound **2**-C_2-CF_3.

N-[2-[2-(4-fluoro-*N*-formyl-anilino)ethyl]phenyl]-2,2-dimethyl-propanamide **2**-C_2-^{t}Bu

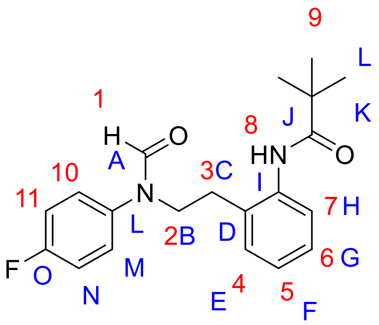

Prepared according to general propylphosphonic anhydride coupling procedure described above. DMF (4 mL), **1**-C_2-H (200 mg, 0.774 mmol), trimethylacetic acid (79 mg, 88.9 µL, 0.774 mmol), propylphosphonic anhydride (50%) in ethyl acetate (542 mg, 507 µL, 0.852 mmol), triethylamine (94.0 mg, 130 µL, 0.929 mmol). Purification by flash column chromatography (SiO_2, *n*-Hex/EtOAc, 1:1, *v*/*v*) yielded a white solid (217 mg, 82%).

^{1}H NMR (500 MHz, DMSO-*d*$_6$) δ = 8.95 (8’, s, 1H), 8.87 (8, s, 1H), 8.31 (1, s, 1H), 8.14 (1’, s, 1H), 7.38–7.32 (10, m, 2H, 10’, m, 2H), 7.28–7.09 (11, m, 2H, 11’, m, 2H, 4, m, 1H, 4’, m, 1H, 5, m, 1H, 5’, m, 1H, 6, m, 1H, 6’, m, 1H, 7, m, 1H, 7’, m, 1H), 3.90–3.84 (2, m, 2H, 2’, m, 2H), 2.71–2.63 (3, m, 2H, 3’, m, 2H), 1.12 (9, s, 9H, 9’, s, 9H).

^{13}C NMR (126 MHz, DMSO-*d*$_6$) δ = 177.42 (s), 177.35 (s), 162.90 (s), 162.46 (s), 160.91 (d, *J* = 243.18 Hz), 160.63 (d, *J* = 243.18 Hz), 137.66 (d, *J* = 2.8 Hz), 137.09 (s), 135.37 (s), 135.28 (s), 130.44 (s), 130.08 (s), 128.70 (s), 128.62 (s), 128.55 (s), 128.28 (s), 127.33 (s), 127.15 (s), 126.87 (d, *J* = 8.5 Hz), 126.73 (s), 116.62 (d, *J* = 22.4 Hz), 116.16 (d, *J* = 22.7 Hz), 67.49 (s), 56.50 (s), 49.52 (s), 45.54 (s), 38.99 (s), 31.31 (s), 29.57 (s), 27.66 (s), 25.60 (s), 19.03 (s).

^{19}F{^{1}H} NMR (471 MHz, DMSO-d$_6$) δ = −115.80 to −115.86 (minor, m), −116.17 to −116.23 (major, m).

EI HRMS: obtained m/z 342.17408 M$^+$ (expected m/z 342.17381 M$^+$).

MP: 122–125 °C.

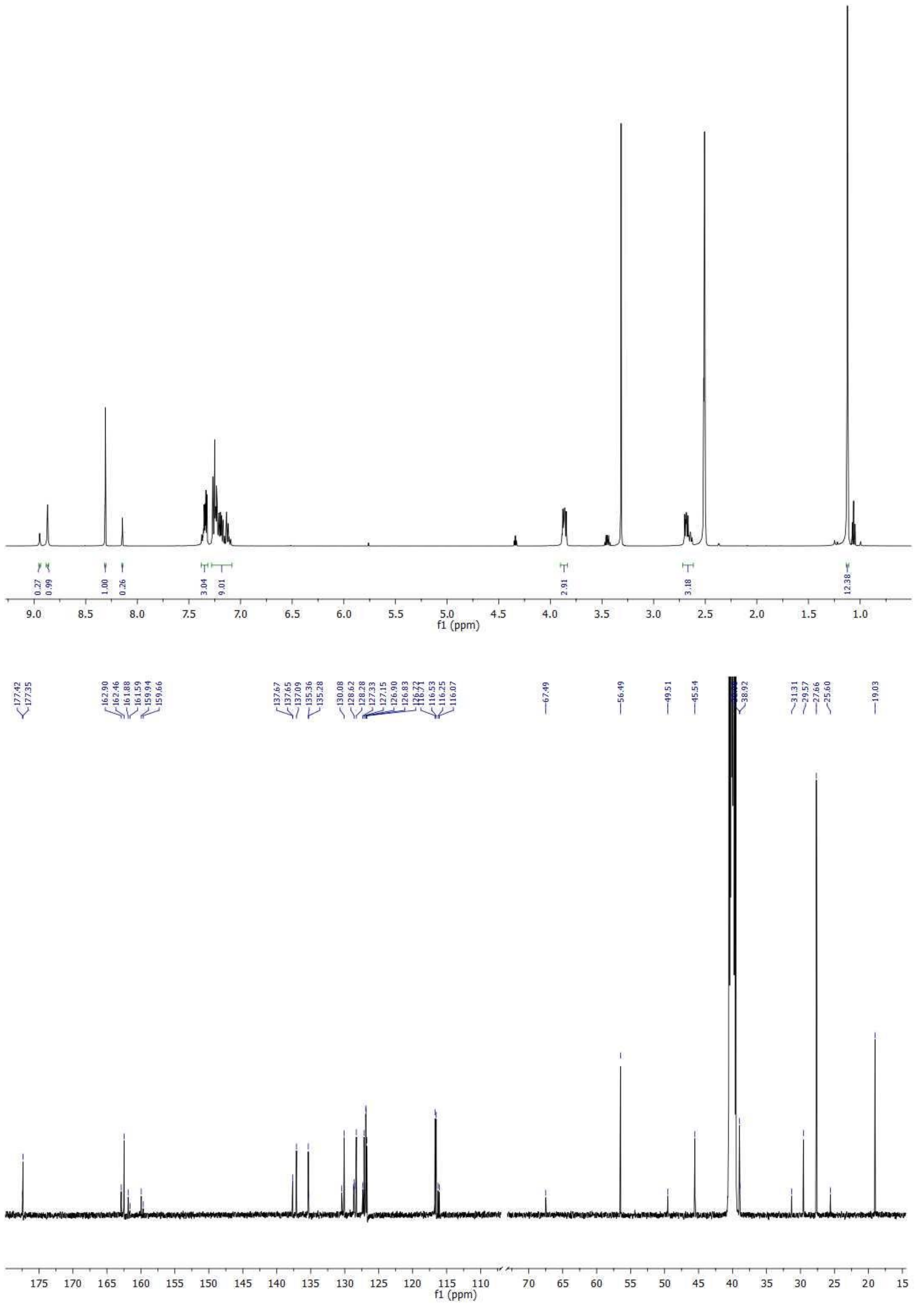

Figure S33 (Top) Partial ^{1}H NMR spectrum (500 MHz, 298 K, DMSO-d_6) and (Bottom) partial ^{13}C NMR spectrum (126 MHz, 298 K, DMSO-d_6) of compound **2**-C_2-^{t}Bu.

N-[2-[2-(4-fluoro-*N*-formyl-anilino)ethyl]phenyl]benzamide **2**-C_2-Ph

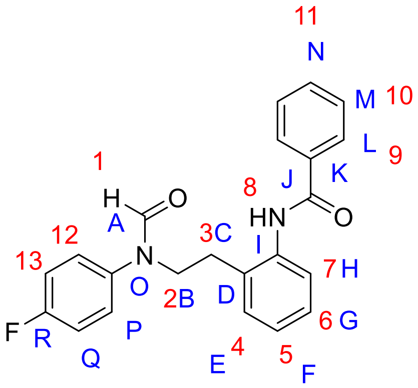

Prepared according to general propylphosphonic anhydride coupling procedure described above. DMF (4 mL), **1**-C_2-H (200 mg, 0.774 mmol), benzoic acid (94.5 mg, 0.774 mmol), propylphosphonic anhydride (50%) in ethyl acetate (542 mg, 507 μL, 0.852 mmol), triethylamine (94.0 mg, 130 μL, 0.929 mmol). Purification by flash column chromatography (SiO_2, *n*-Hex/EtOAc, 1:1, *v*/*v*) yielded a white solid (205 mg, 73%).

^{1}H NMR (601 MHz, DMSO-*d*$_6$) δ = 9.92 (8', s, 1H), 9.89 (8, s, 1H), 8.31 (1, s, 1H), 8.17 (1', s, 1H), 7.91–7.84 (9, m, 2H, 9', m, 2H), 7.64 – 7.58 (11, m, 1H, 11', m, 1H), 7.54–7.49 (10, m, 2H, 10', m, 2H), 7.34–7.21 (12, m, 2H, 12', m, 2H, 4, m, 1H, 4', m, 1H, 7, m, 1H, 7', m, 1H, 6, m, 1H, 6', m, 1H, 5, m, 1H, 5', m, 1H), 7.04–6.99 (13, m, 2H, 13', m, 2H), 3.92–3.86 (2, m, 2H, 2', m, 2H), 2.82–2.73 (3, m, 2H, 3', m, 2H).

^{13}C NMR (126 MHz, DMSO-*d*$_6$) δ = 166.35 (J, s), 162.83 (A', s), 162.43 (A, s), 160.61 (R, d, *J* = 244.44 Hz), 160.32 (R', d, *J* = 243. 18 Hz), 137.49 (O, d, J = 2.6 Hz), 136.81 (I, s), 136.78 (I', s), 135.35 (D, s), 135.18 (O, d, *J* = 1.9 Hz), 134.75 (K, s), 134.60 (K', s), 132.06 (N', s), 131.95 (N, s), 130.81 (H', s), 130.47 (H, s), 128.82 (M', s), 128.74 (M, s), 128.62 (F', s), 128.34 (F, s), 128.09 (L, s), 127.96 (L', s), 127.58 (G', s), 127.44 (G, s), 127.14 (E, s), 127.11 (E', s), 126.09 (P, d, *J* = 8.5 Hz), 116.45 (Q, d, *J* = 22.6 Hz), 115.94 (Q', d, *J* = 22.5 Hz), 49.57 (B', s), 45.37 (B, s), 31.57 (C', s), 29.91 (C, s).

^{19}F{^{1}H} NMR (471 MHz, DMSO-*d*$_6$) δ = −115.90 (minor), −116.36 (major).

EI HRMS: obtained m/z 362.14178 M$^+$ (expected m/z 362.14251 M$^+$).

MP: 128–130 °C.

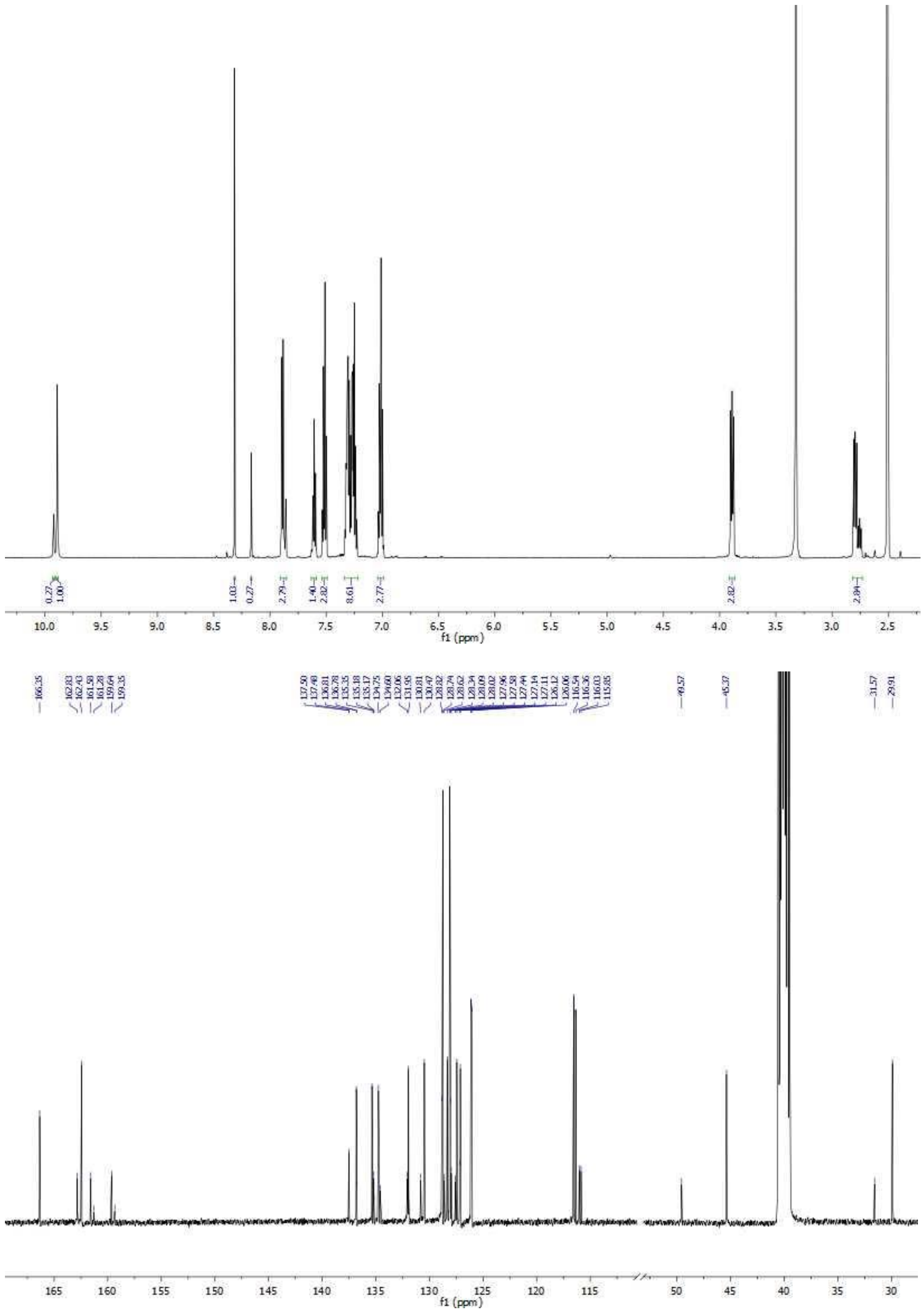

Figure S34 (Top) Partial ^{1}H NMR spectrum (500 MHz, 298 K, DMSO-d_6) and (Bottom) partial ^{13}C NMR spectrum (126 MHz, 298 K, DMSO-d_6) of compound **2**-C_2-Ph.

N-[2-[2-(4-fluoro-*N*-formyl-anilino)ethyl]phenyl]-4-methoxy-benzamide **2**-C_2-PhOMe

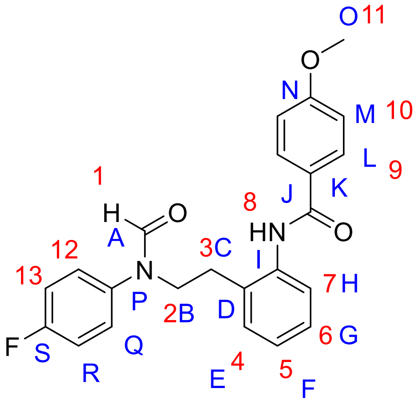

Prepared according to general propylphosphonic anhydride coupling procedure described above. DMF (4 mL), **1**-C_2-H (200 mg, 0.774 mmol), 4-methoxybenzoic acid (118 mg, 0.774 mmol), propylphosphonic anhydride (50%) in ethyl acetate (542 mg, 507 μL, 0.852 mmol), triethylamine (94.0 mg, 130 μL, 0.929 mmol). Purification by flash column chromatography (SiO_2, *n*-Hex/EtOAc, 1:1, *v*/*v*) yielded a white solid (156 mg, 51%).

^{1}H NMR (601 MHz, DMSO-*d*$_6$) δ = 9.77 (8', s, 1H), 9.73 (8, s, 1H), 8.31 (1, s, 1H), 8.15 (1', s, 1H), 7.89–7.84 (9, m, 2H, 9', 2H), 7.32–7.20 (12', m, 2H, 12, m, 2H, 4, m, 1H, 5, m, 1H, 6, m, 1H, 7, m, 1H, 4', m, s, 5', m, s, 6', m, s, 7', m, s), 7.06–6.99 (13, m, 2H, 13', m, 2H, 10, m, 2H, 10', m, 2H), 3.90–3.85 (2, m, 2H, 11, m, 3H, 2', m, 2H, 11', m, 3H), 2.81–2.72 (3, m, 2H, 3', m, 2H).

^{13}C NMR (126 MHz, DMSO-*d*$_6$) δ = 165.77 (J', s), 165.74 (J, s), 162.82 (A', s), 162.42 (A, s), 162.38 (N', s), 162.32 (N, s), 160.61 (S, d, *J* = 243.18 Hz), 160.32 (S', d, *J* = 243.18 Hz), 137.48 (P, d, *J* = 2.5 Hz), 137.01 (I, s), 136.99 (I', s), 135.34 (D, s), 135.21 (D', s), 135.18 (P', d, *J* = 2.5 Hz), 130.77 (H', s), 130.42 (H, s), 129.99 (L, s), 129.93 (L', s), 128.68 (F', s), 128.36 (F, s), 127.97 (Q', d, *J* = 8.4 Hz), 127.53 (G', s), 127.38 (G, s), 126.99 (E', s), 126.94 (E, s), 126.87 (K, s), 126.72 (K', s), 126.06 (Q, d, *J* = 8.5 Hz), 116.45 (R, d, J = 22.5 Hz), 115.95 (R', d, J = 22.5 Hz), 114.03 (M', s), 113.95 (M, s), 55.90 (O', s), 55.89 (O, s), 49.56 (B', s), 45.38 (B, s), 31.64 (C', s), 29.93 (C, s).

^{19}F{^{1}H} NMR (471 MHz, DMSO-*d*$_6$) δ = −115.92 (minor), −116.40 (major).

EI HRMS: obtained m/z 392.15346 M$^+$ (expected m/z 392.15307 M$^+$).

MP: 136–138 °C.

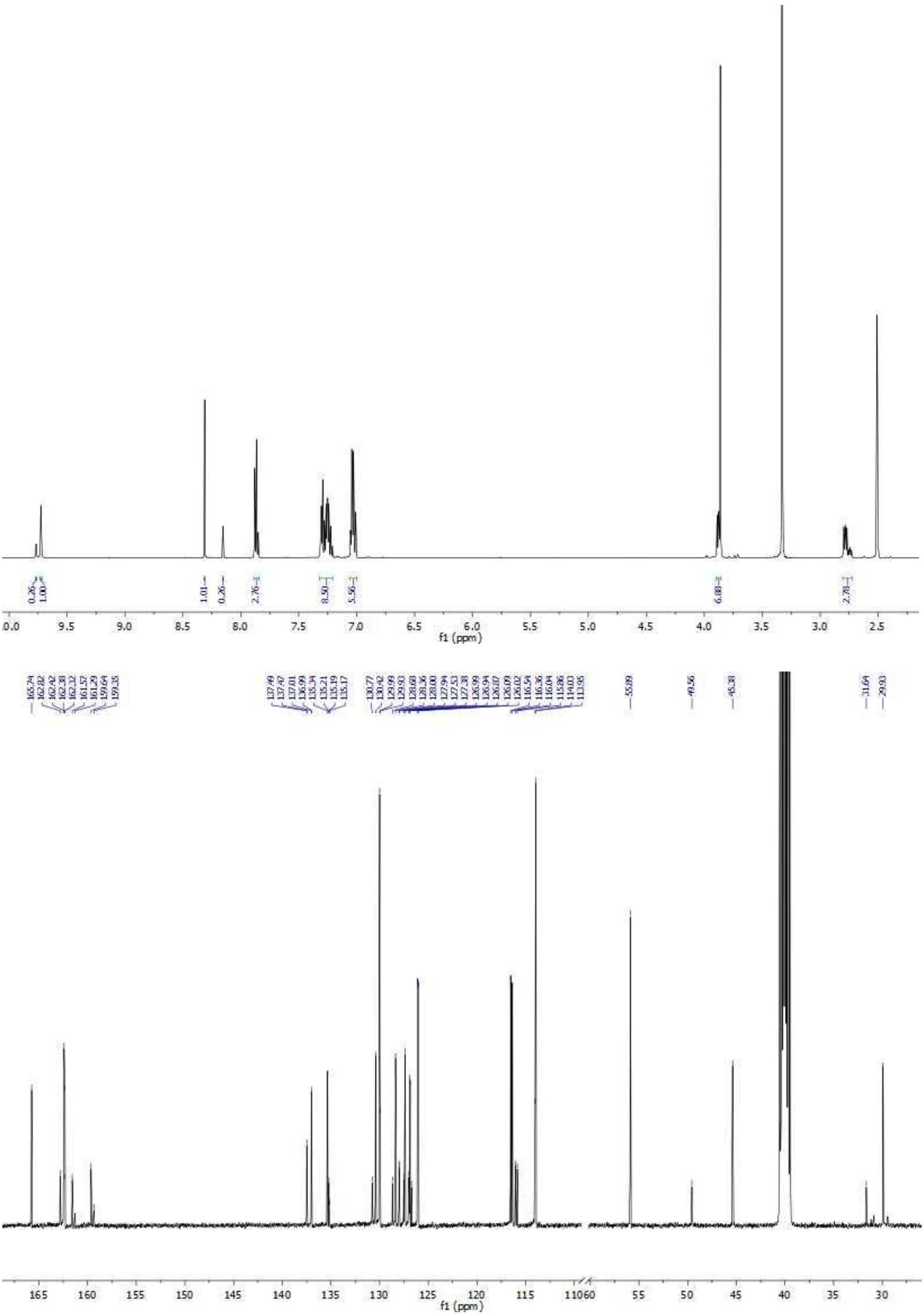

Figure S35 (Top) Partial ^{1}H NMR spectrum (500 MHz, 298 K, DMSO-d_6) and (Bottom) partial ^{13}C NMR spectrum (126 MHz, 298 K, DMSO-d_6) of compound **2**-C_2-PhOMe.

N-[2-[2-(4-fluoro-*N*-formyl-anilino)ethyl]phenyl]pyridine-2-carboxamide **2**-C_2-Pyr

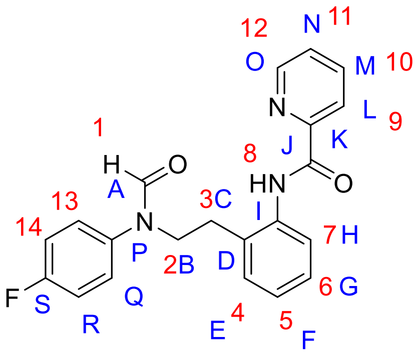

Prepared according to general propylphosphonic anhydride coupling procedure described above. DMF (4 mL), **1**-C_2-H (200 mg, 0.774 mmol), 2-picolinic acid (95.3 mg, 0.774 mmol), propylphosphonic anhydride (50%) in ethyl acetate (542 mg, 507 μL, 0.852 mmol), triethylamine (94.0 mg, 130 μL, 0.929 mmol). Purification by flash column chromatography (SiO_2, *n*-Hex/EtOAc, 1:1, *v*/*v*) yielded a white solid (104 mg, 37%).

^{1}H NMR (601 MHz, DMSO-d_6) δ = 10.31 (8’, s, 1H), 10.30 (8, m, 1H), 8.70 – 8.65 (9, m, 1H, 9’, m, 1H), 8.30 (1, s, 1H), 8.18 (1’, s, 1H), 8.12 – 8.04 (11, m, 1H, 11’, m, 1H, 12, m, 1H, 12’, m, 1H), 7.72–7.68 (10, m, 1H, 10’, m, 1H), 7.60–7.55 (7, m, 1H, 7’, m, 1H), 7.34–7.18 (13, m, 2H, 13’, m, 2H, 6, m, 1H, 6’, m, 1H, 4, m, 1H, 5, m, 1H, 4’, m, 1H, 5’, m, 1H), 7.03–6.99 (14, m, 2H, 14’, m, 2H), 3.96–3.89 (2, m, 2H, 2’, m, 2H), 2.86–2.77 (3, m, 2H, 3’, m, 2H).

^{13}C NMR (126 MHz, DMSO-d_6) δ = 163.25 (J’, s), 163.17 (J, s), 162.88 (A’, s), 162.43 (A, s), 160.55 (S, d, *J* = 243.18 Hz), 160.25 (S’, d, *J* = 243. 18 Hz), 150.04 (L’, s), 148.88 (L, s), 138.54 (O’, s), 138.50 (O, s), 137.36 (P, d, *J* = 2.9 Hz), 136.37 (I, s), 135.10 (P’, d, *J* = 3.0 Hz), 133.45 (K, s), 133.20 (K’, s), 130.78 (E’, s), 130.53 (E, s), 127.87 (Q’, d, *J* = 8.5 Hz), 127.64 (F’, s), 127.53 (F, s), 127.38 (M’, s), 127.33 (M, s), 126.50 (G, s), 126.22 (H, s), 125.95 (Q, d, *J* = 8.5 Hz), 122.72 (N’, s), 122.68 (N, s), 116.38 (R, d, *J* = 22.8 Hz), 115.86 (R’, d, *J* = 22.4 Hz), 49.35 (B’, s), 44.85 (B, s), 31.41 (C’, s), 29.80 (C, s).

^{19}F{^{1}H} NMR (471 MHz, DMSO-d_6) δ −115.94 (minor), −116.39 (major).

EI HRMS: obtained m/z 363.13657 M^+ (expected m/z 363.13665 M^+).

MP: 85–86 °C.

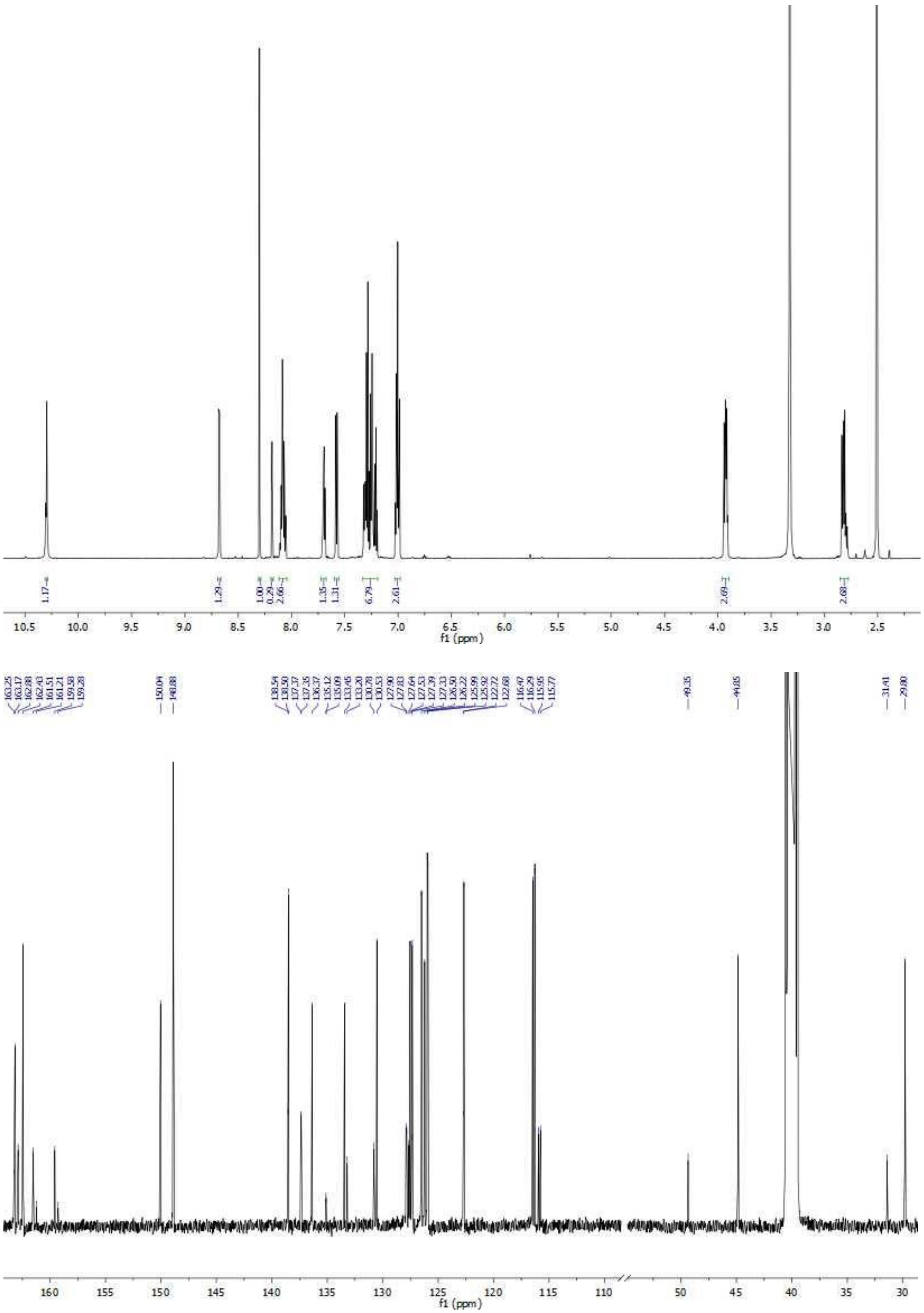

Figure S36 (Top) Partial ^{1}H NMR spectrum (500 MHz, 298 K, DMSO-d_6) and (Bottom) partial ^{13}C NMR spectrum (126 MHz, 298 K, DMSO-d_6) of compound **2**-C_2-Pyr.

5-fluoro-*N*-[2-[2-(4-fluoro-*N*-formyl-anilino)ethyl]phenyl]pyridine-2-carboxamide **2**-C_2-PyrF

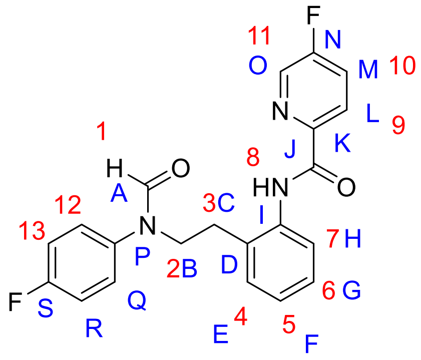

Prepared according to general propylphosphonic anhydride coupling procedure described above. DMF (4 mL), **1**-C_2-H (200 mg, 0.774 mmol), 5-fluoro-2-pyridinecarboxylic acid (109 mg, 0.774 mmol), propylphosphonic anhydride (50%) in ethyl acetate (542 mg, 507 μL, 0.852 mmol), triethylamine (94.0 mg, 130 μL, 0.929 mmol). Purification by flash column chromatography (SiO_2, *n*-Hex/EtOAc, 1:1, *v*/*v*) yielded a white solid (100 mg, 34%).

^{1}H NMR (601 MHz, DMSO-d_6) δ = 10.22 (8', s, 1H), 10.21 (8, s, 1H), 8.65 (9, m, 1H, 9', m, 1H), 8.28 (1, s, 1H), 8.20–8.09 (11, m, 1H, 11', m, 1H), 8.03–7.93 (10, m, 1H, 10', m, 1H), 7.53–7.44 (7, m, 1H, 7', m, 1H), 7.33–7.26 (12', m, 2H, 6, m, 1H, 6', m, 1H, 5, m, 1H, 5', m, 1H), 7.26–7.19 (12, m, 2H, 4, m, 1H, 4', m, 1H), 7.05–7.00 (13, m, 2H, 13', m, 2H), 3.96–3.80 (2, m, 2H, 2', m, 2H), 2.86–2.68 (3, m, 2H, 3', m, 2H).

^{13}C NMR (126 MHz, DMSO-d_6) δ = 162.85 (s), 162.43 (s), 162.40 (s), 162.36 (s), 160.58 (S, d, *J* = 243.18 Hz), 160.31 (s), 160.29 (s), 160.26 (S', d, *J* = 244.44 Hz), 146.82 (d, J = 3.8 Hz), 137.36 (s), 137.34 (s), 137.13 (s), 136.31 (s), 135.11 (d, J = 3.0 Hz), 133.99 (s), 133.79 (s), 130.75 (s), 130.51 (s), 127.96 (d, J = 8.5 Hz), 127.60 (s), 127.50 (s), 127.07 (s), 126.78 (s), 126.74 (s), 126.13 (d, J = 8.5 Hz), 125.21 (s), 125.16 (s), 125.06 (s), 124.98 (s), 124.93 (s), 116.38 (R, d, *J* = 22.8 Hz), 115.86 (R', d, *J* = 22.5 Hz), 49.47 (B', s), 45.02 (B, s), 31.37 (C', s), 29.79 (C, s).

EI HRMS: obtained m/z 381.12593 M^+ (expected m/z 381.12451 M^+).

MP: 95–96 °C.

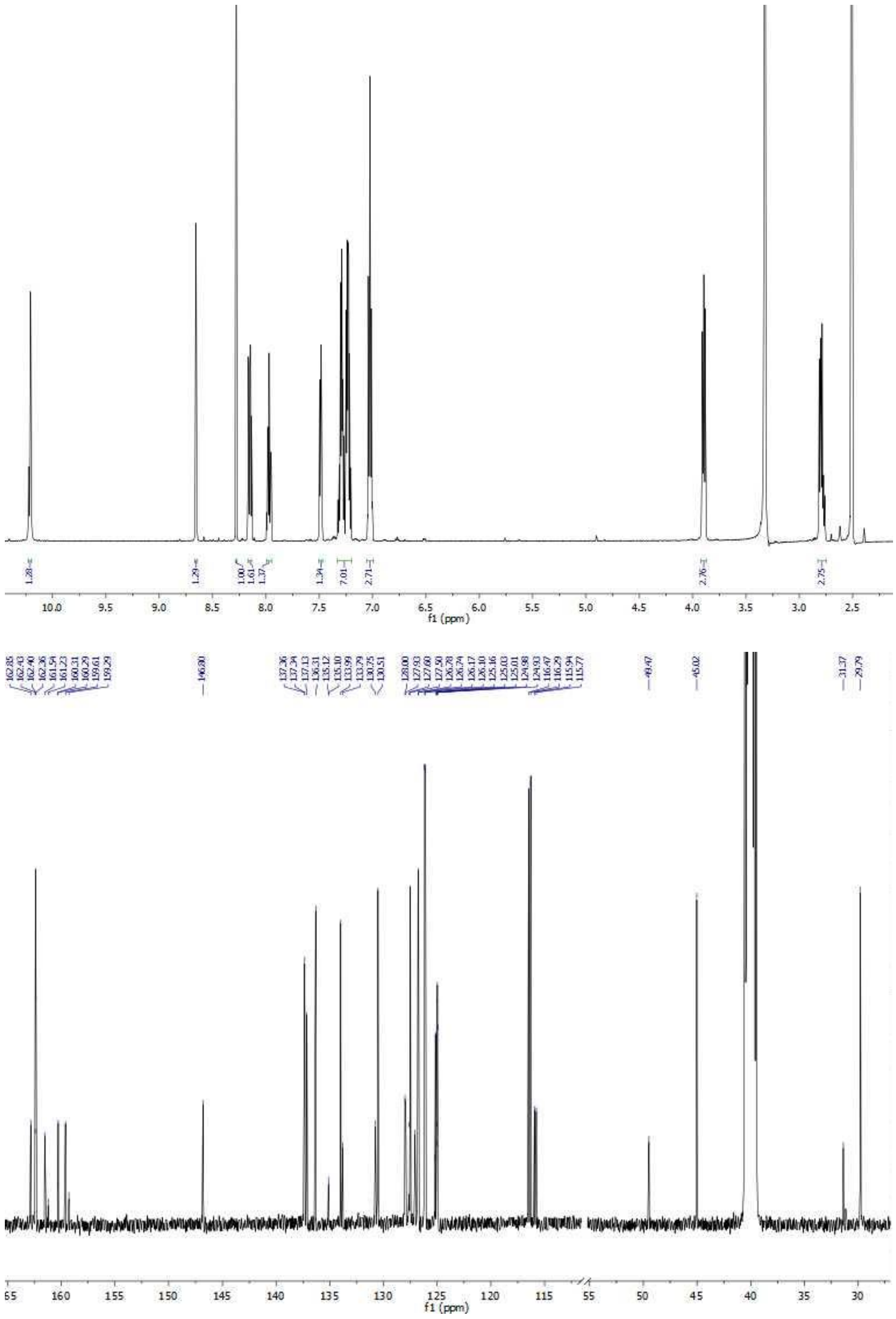

Figure S37 (Top) Partial ^{1}H NMR spectrum (500 MHz, 298 K, DMSO-d_6) and (Bottom) partial ^{13}C NMR spectrum (126 MHz, 298 K, DMSO-d_6) of compound **2**-C_2-PyrF.

S2.3 Conformer assignment by NMR spectroscopy

Figures S39–S43 present the NMR spectra (^{1}H, ^{13}C, HSQC and HMBC) of *N*-[2-[(4-fluoro-*N*-formyl-anilino)methyl]phenyl]-2,2-dimethyl-propanamide (compound **2**-C_1-^{t}Bu) in DMSO-d_6, showing the full spectral assignment for both conformers. The proton resonances have been labelled numerically and carbon resonances alphabetically. Red peaks with prime notation (') denotes a minor conformer, while blue indicates the major conformer (Figure S38).

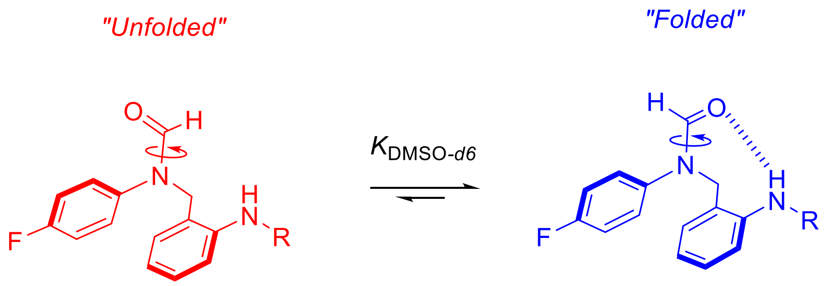

Figure S38 Equilibrium between the unfolded or open conformation and the folded or closed conformation of the molecular balances.

The major and minor conformers were unambiguously distinguished by analysing the HMBC spectra (Figure S43); H-C correlation through multiple bonds. The formyl proton has a cross peak with the *trans*-CH_2 carbon in the H-bonded conformer, whereas in the unbound conformer the formyl proton has a *trans*-cross peak with trans-quaternary aromatic carbon.

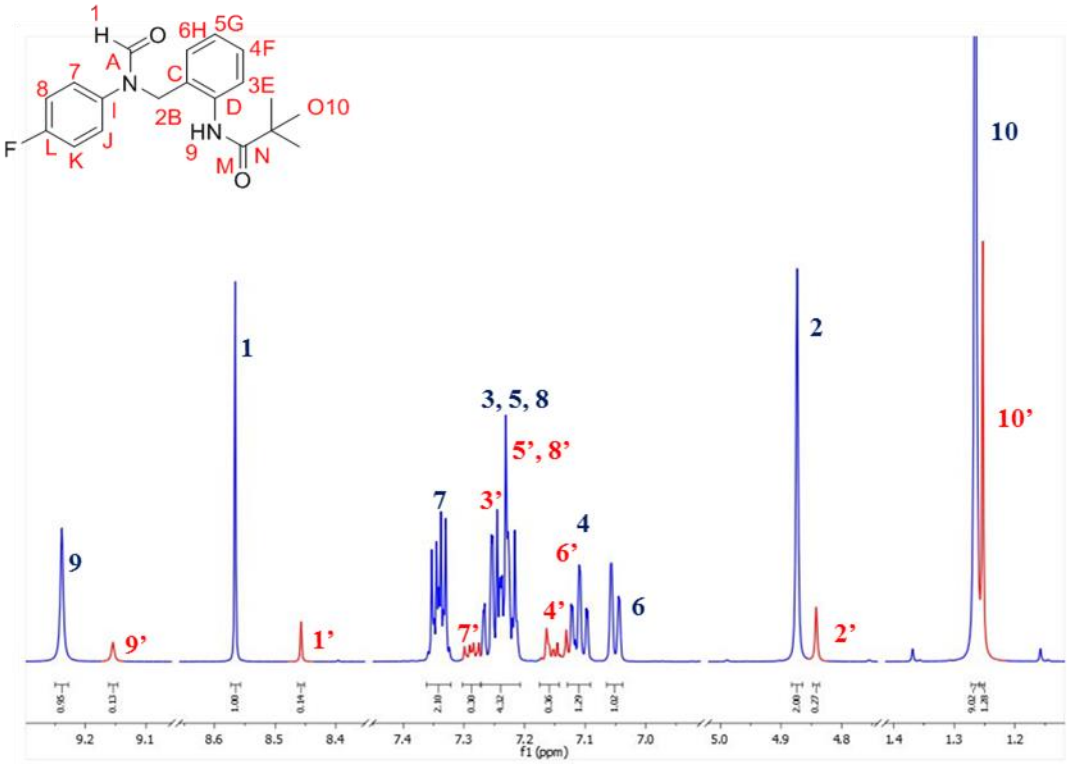

Figure S39 Partial ^{1}H NMR spectrum (500 MHz, 298 K, DMSO-d_6) of compound **2**-C_1-^{t}Bu showing full assignment of all peaks for both the open (unfolded) and closed (folded) conformers.

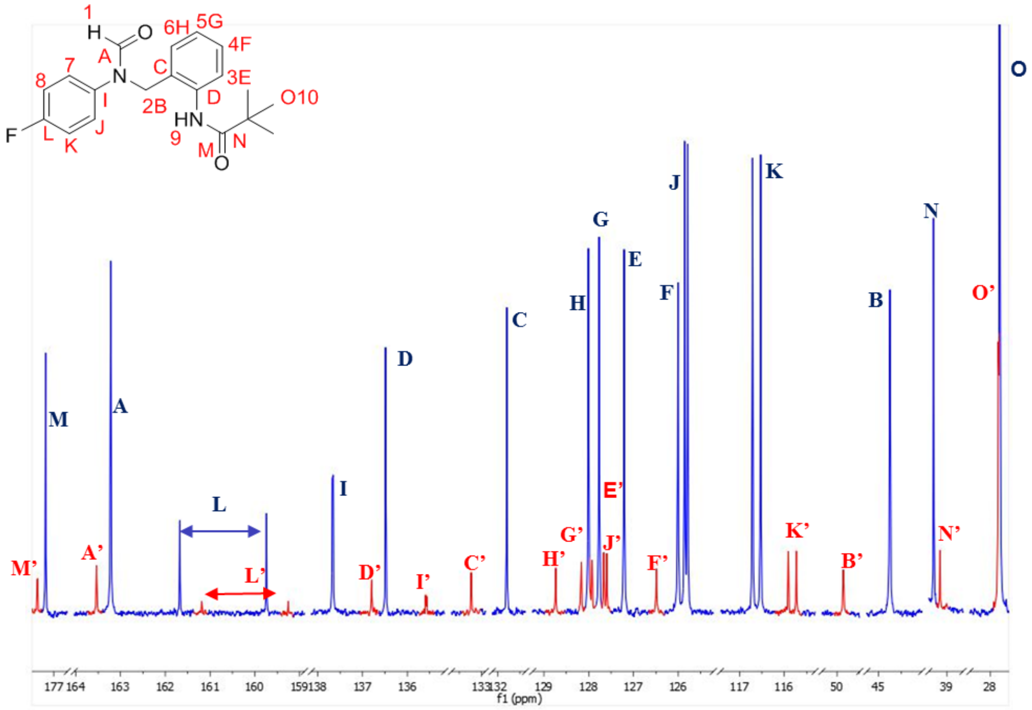

Figure S40 Partial ^{13}C NMR spectrum (126 MHz, 298 K, DMSO-d_6) of compound **2**-C_1-^{t}Bu showing full assignment of all peaks for both the open (unfolded) and closed (folded) conformers.

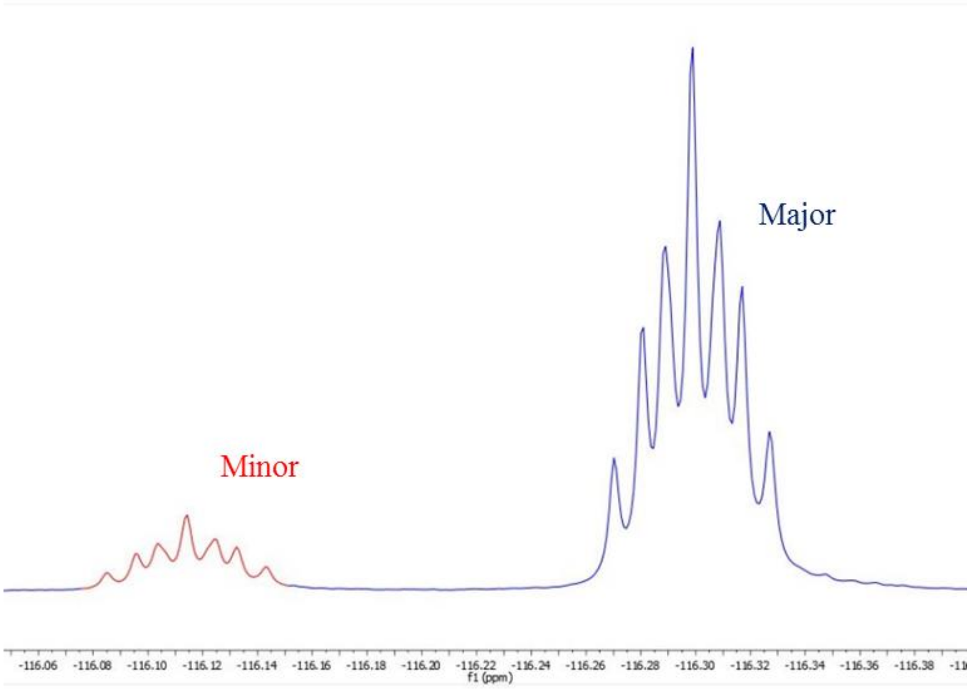

Figure S41 Partial ^{19}F NMR spectrum (471 MHz, 298 K, DMSO-d_6) of compound **2**-C_1-^tBu showing full assignment of all peaks for both the open (unfolded) and closed (folded) conformers.

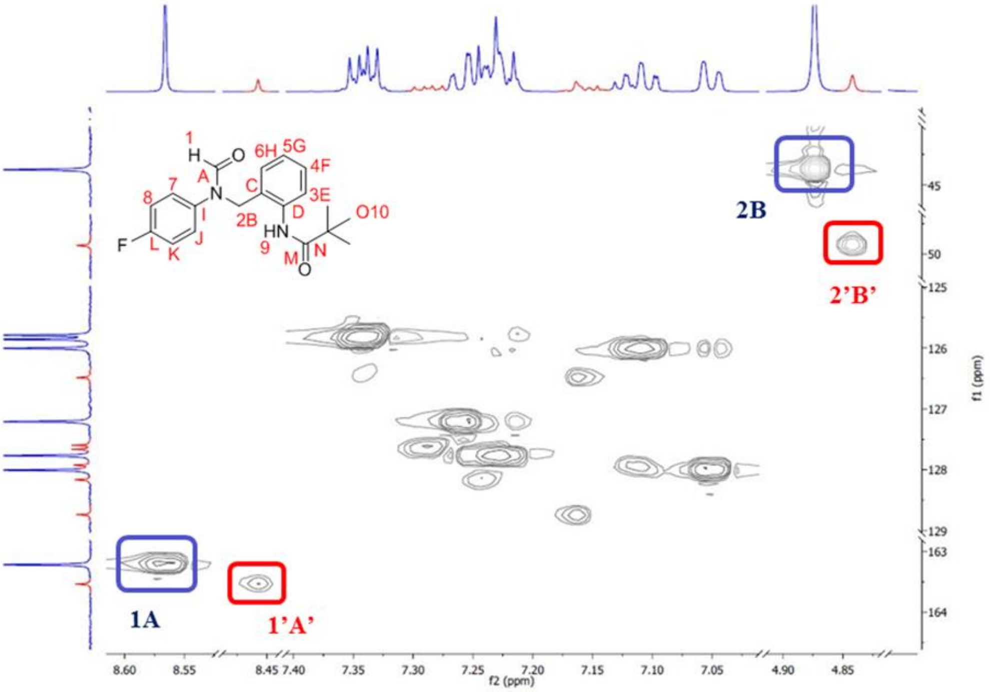

Figure S42 Partial HSQC ^1H-^{13}C NMR spectrum (500 MHz, 298 K, DMSO-d_6) of compound **2**-C_1-^tBu showing assignment of peaks for both the open (unfolded) and closed (folded) conformers.

In the example depicted in Figure S42, the major formyl proton 1 couples to carbon B, and the minor formyl proton 1' couples with I'.

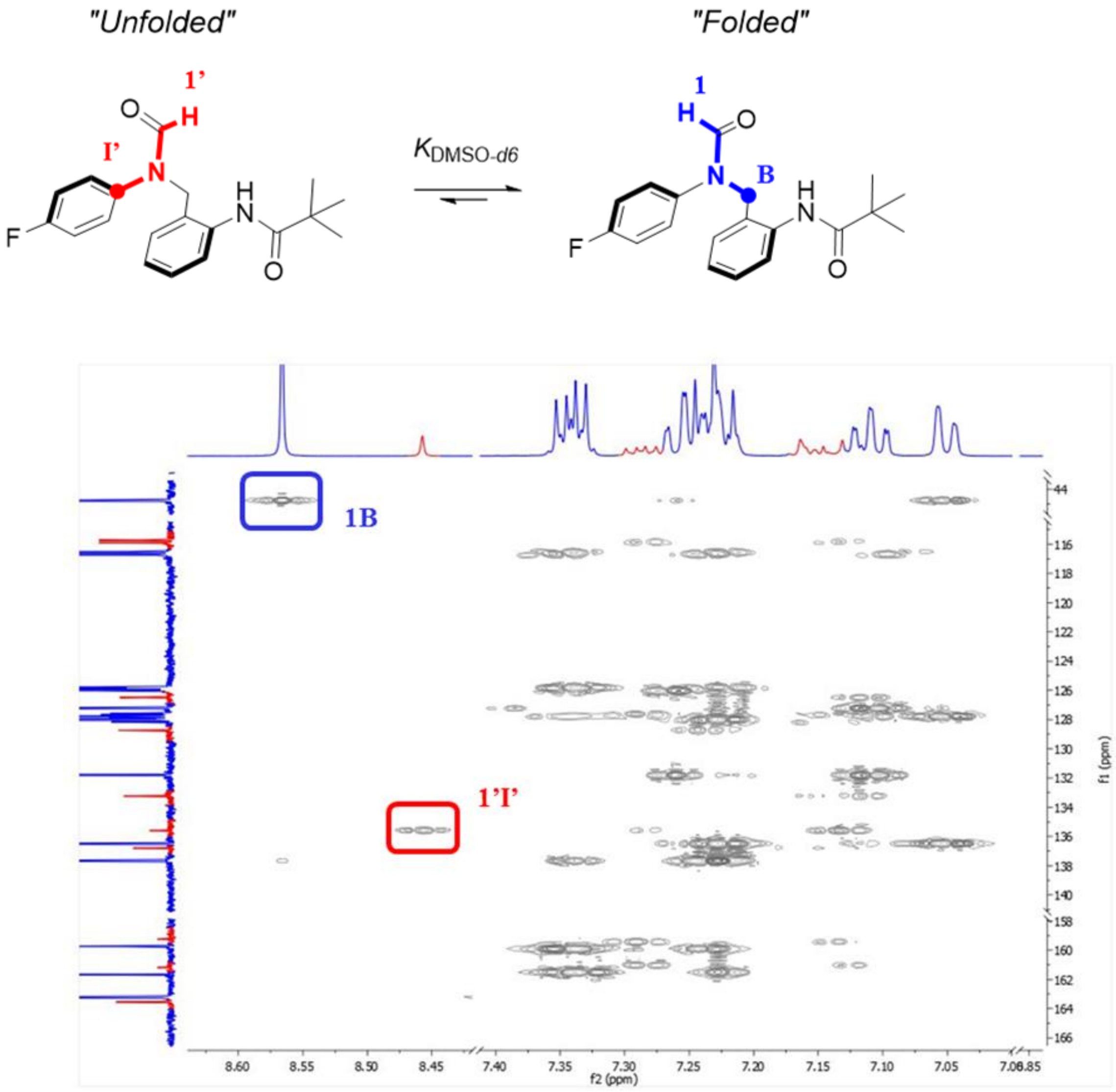

Figure S43 Partial HMBC ^{1}H-^{13}C NMR spectrum (500 MHz, 298 K, DMSO-d_6) of compound **2**-C_1-^{t}Bu showing assignment of peaks for both the open (unfolded) and closed (folded) conformers.

S2.4 Crystal Structure of 2-C_1-Me

Experimental: Single colourless block-shaped crystals of **2**-C_1-Me were recrystallized from DCM by slow evaporation. A suitable crystal with dimensions 0.34 × 0.16 × 0.05 mm^3 was selected and mounted on a MITIGEN holder in Paratone oil on a Rigaku Oxford Diffraction SuperNova diffractometer. The crystal was kept at a steady T = 120.00(10) K during data collection. The structure was solved with the **ShelXS** solution program[S7] using direct methods and by using **Olex2** 1.5-beta[S5] as the graphical interface. The model was refined with **olex2.refine** 1.5-beta.[S6] Aspherical atomic form factors were included using the NoSpherA2 module of Olex2[S8] and ORCA[S9] for wavefunction calculations.

Crystal Data: $C_{16}H_{15}FN_2O_2$, M_r = 286.308, triclinic, P-1 (No. 2), a = 7.7752(4) Å, b = 9.3986(5) Å, c = 10.6511(7) Å, α = 101.842(5)°, β = 102.717(5)°, γ = 108.474(5)°, V = 687.59(8) Å^3, T = 120.0 K, Z = 2, Z' = 1, $\mu(MoK_\alpha)$ = 0.102, 12391 reflections measured, 3454 unique (R_{int} = 0.0392) which were used in all calculations. The final wR_2 was 0.0554 (all data) and R_1 was 0.0384 (I≥2 σ(I)).

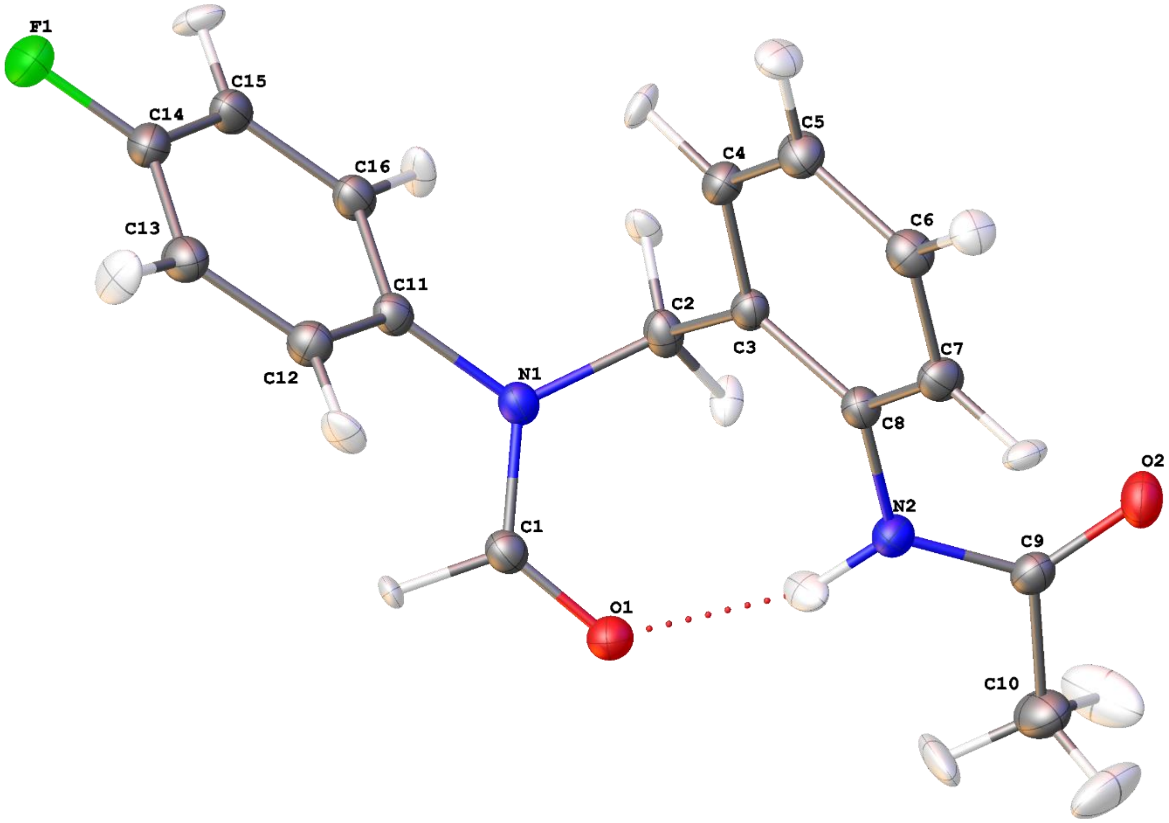

Figure S44 The molecular crystal structure of compound **2**-C_1-Me. Displacement ellipsoids are at the 50% probability level.

Table S1 Crystallographic data for molecular balance **2**-C_1-Me.

**Compound**	**2**-C_1-Me
Formula	$C_{16}H_{15}FN_2O_2$
$D_{calc.}$/ g cm^{-3}	1.383
μ/mm^{-1}	0.102
Formula Weight	286.30
Colour	colourless
Shape	block
Size/mm^3	0.34×0.16×0.05
T/K	120.0
Crystal System	triclinic
Space Group	P-1
a/Å	7.7752(4)
b/Å	9.3986(5)
c/Å	10.6511(7)
α/°	101.842(5)
β/°	102.717(5)
γ/°	108.474(5)
V/Å^3	687.59(8)
Z	2
Z'	1
Wavelength/Å	0.71073
Radiation type	MoK□
Θ_{min}/°	3.516
Θ_{max}/°	29.678
Measured Refl.	12391
Independent Refl.	3454
Reflections Used	2726
R_{int}	0.0392
Parameters	325
Restraints	0
Largest Peak	0.2588
Deepest Hole	-0.2601
GooF	1.1629
wR_2 (all data)	0.0554
wR_2	0.0508
R_1 (all data)	0.0561
R_1	0.0384

Table S2 Fractional Atomic Coordinates (×10^4) and Equivalent Isotropic Displacement Parameters (Å^2×10^3) for **2**-C_1-Me. U_{eq} is defined as 1/3 of the trace of the orthogonalized U_{ij}.

Atom	x	y	z	U_{eq}
F1	9354.7(9)	-149.7(8)	6222.1(6)	32.38(18)
O1	2905.6(9)	4351.4(8)	5526.9(7)	21.31(18)
O2	3266.0(10)	8212.3(9)	9716.9(7)	24.12(19)
N1	4699.5(11)	3201.6(10)	6618.5(8)	16.6(2)
N2	3863.6(13)	6731.8(11)	8016.6(10)	19.7(2)
C1	3881.3(14)	3542.2(12)	5529.3(11)	17.5(2)
C2	4426.9(17)	3787.0(13)	7926.2(11)	17.6(2)
C3	5926.7(14)	5377.4(12)	8762.8(10)	15.5(2)
C4	7666.0(15)	5464.2(13)	9563.7(10)	18.1(2)
C5	9074.7(16)	6895.0(13)	10375.6(11)	20.3(2)
C6	8752.0(16)	8273.3(14)	10396.2(11)	19.8(2)
C7	7031.4(15)	8220.1(13)	9617.8(11)	18.8(2)
C8	5617.1(14)	6776.6(12)	8802.9(10)	16.3(2)
C9	2795.2(14)	7459.8(12)	8533.8(11)	18.7(2)
C10	973.7(19)	7259(2)	7522.2(15)	29.1(3)
C11	5923.8(13)	2354.1(12)	6529.8(10)	16.0(2)
C12	7403.1(15)	2835.5(14)	5983.0(11)	19.1(2)
C13	8565.5(16)	1983.4(14)	5861.1(11)	21.9(3)
C14	8225.6(15)	677.2(13)	6317.1(10)	22.4(3)
C15	6793.1(16)	190.7(13)	6888.4(11)	21.2(2)
C16	5624.4(16)	1035.3(13)	6988.5(11)	18.9(2)

Table S3 Anisotropic Displacement Parameters (×10[4]) of **2**-C_1-Me. The anisotropic displacement factor exponent takes the form: $-2\pi^2[h^2a^{*2} \times U_{11} + ... + 2hka^* \times b^* \times U_{12}]$.

Atom	U_{11}	U_{22}	U_{33}	U_{23}	U_{13}	U_{12}
F1	43.2(4)	39.9(4)	28.7(4)	30.7(4)	14.3(3)	13.1(3)
O1	24.0(4)	23.1(4)	18.1(4)	12.3(4)	4.4(3)	5.5(3)
O2	26.0(4)	23.4(4)	23.5(5)	12.2(4)	9.0(3)	2.1(3)
N1	20.2(5)	15.2(5)	14.9(5)	6.8(4)	6.2(4)	4.5(4)
N2	23.0(5)	20.5(5)	16.8(5)	12.2(5)	4.0(4)	4.1(4)
H2	22(9)	71(14)	36(10)	24(9)	10(8)	17(10)
C1	19.5(6)	17.2(6)	14.8(6)	6.6(5)	4.8(5)	3.9(5)
H1	55(9)	42(9)	12(7)	32(7)	12(6)	5(6)
C2	22.1(6)	15.4(6)	16.8(6)	6.4(5)	9.6(5)	4.7(5)
H2a	14(7)	26(8)	42(9)	0(6)	9(6)	-5(6)
H2b	48(8)	25(8)	24(8)	9(7)	19(6)	13(7)
C3	19.0(5)	15.3(5)	15.1(5)	8.1(5)	7.4(4)	5.4(4)
C4	20.9(6)	18.4(6)	18.2(6)	11.0(5)	6.7(5)	5.5(5)
H4	41(8)	22(8)	51(9)	22(7)	11(7)	4(7)
C5	18.5(6)	22.8(6)	19.7(6)	9.1(6)	5.3(5)	5.0(5)
H5	37(8)	41(9)	34(9)	10(7)	-6(7)	4(7)
C6	18.8(6)	19.3(6)	18.8(6)	5.0(5)	5.3(5)	4.7(5)
H6	27(8)	30(9)	42(9)	1(7)	4(7)	6(7)
C7	22.7(6)	14.5(6)	18.7(6)	6.7(5)	5.3(5)	5.4(5)
H7	38(8)	34(9)	37(8)	20(7)	0(6)	13(7)
C8	19.2(5)	16.1(6)	14.3(5)	7.7(5)	5.3(4)	3.8(4)
C9	21.3(6)	18.5(6)	19.8(6)	10.4(5)	7.1(5)	7.8(5)
C10	27.6(7)	39.6(9)	28.1(8)	20.5(7)	8.9(6)	13.9(7)
H10a	80(13)	46(12)	120(16)	36(11)	-12(11)	19(12)
H10b	72(11)	119(16)	19(9)	54(11)	12(8)	0(10)
H10c	27(9)	170(20)	74(13)	15(11)	26(9)	57(13)
C11	18.5(5)	13.6(5)	14.0(5)	4.9(5)	4.5(4)	2.7(4)
C12	20.3(6)	19.4(6)	19.4(6)	7.7(5)	7.9(5)	7.4(5)
H12	52(9)	43(10)	29(9)	11(8)	24(7)	20(8)
C13	23.0(6)	27.5(7)	19.4(6)	13.2(6)	8.0(5)	8.3(5)
H13	38(8)	54(10)	74(11)	22(8)	45(8)	35(9)
C14	28.5(6)	25.0(6)	17.7(6)	16.1(6)	6.4(5)	5.7(5)
C15	31.4(7)	17.8(6)	17.3(6)	12.2(5)	8.2(5)	5.8(5)
H15	48(9)	49(10)	54(10)	30(8)	15(8)	36(9)
C16	25.1(6)	15.5(6)	16.6(6)	7.4(5)	7.6(5)	5.0(4)
H16	60(9)	31(9)	44(9)	17(8)	41(8)	20(7)

Table S4 Bond lengths in Å for **2**-C_1-Me.

Atom	Atom	Length/Å
F1	C14	1.3507(11)
O1	C1	1.2327(12)
O2	C9	1.2203(12)
N1	C1	1.3452(12)
N1	C2	1.4755(13)
N1	C11	1.4279(12)
N2	C8	1.4171(13)
N2	C9	1.3669(13)
C2	C3	1.5098(15)
C3	C4	1.3989(13)
C3	C8	1.4040(13)
C4	C5	1.3886(15)
C5	C6	1.3922(15)
C6	C7	1.3899(15)
C7	C8	1.3979(14)
C9	C10	1.5092(16)
C11	C12	1.3928(14)
C11	C16	1.3946(14)
C12	C13	1.3939(15)
C13	C14	1.3814(15)
C14	C15	1.3819(15)
C15	C16	1.3900(15)

Table S5 Bond angles in ° for **2**-C_1-Me.

Atom	Atom	Atom	Angle/°
C2	N1	C1	119.99(9)
C11	N1	C1	120.04(8)
C11	N1	C2	119.86(8)
C9	N2	C8	123.23(9)
N1	C1	O1	124.80(10)
C3	C2	N1	112.98(9)
C4	C3	C2	119.12(9)
C8	C3	C2	122.33(9)
C8	C3	C4	118.51(10)
C5	C4	C3	121.36(10)
C6	C5	C4	119.44(10)
C7	C6	C5	120.40(11)
C8	C7	C6	119.95(10)
C3	C8	N2	120.07(9)
C7	C8	N2	119.60(9)
C7	C8	C3	120.33(9)
N2	C9	O2	123.06(9)
C10	C9	O2	122.35(10)
C10	C9	N2	114.59(10)
C12	C11	N1	120.28(9)
C16	C11	N1	119.59(9)
C16	C11	C12	120.13(9)
C13	C12	C11	120.32(11)
C14	C13	C12	118.16(11)
C13	C14	F1	118.89(10)
C15	C14	F1	118.34(10)
C15	C14	C13	122.76(10)
C16	C15	C14	118.66(11)
C15	C16	C11	119.95(11)

Table S6 Torsion angles in ° for **2**-C_1-Me.

Atom	Atom	Atom	Atom	Angle/°
F1	C14	C13	C12	-179.16(10)
F1	C14	C15	C16	-179.94(9)
O1	C1	N1	C2	1.14(13)
O1	C1	N1	C11	-175.12(10)
O2	C9	N2	C8	0.15(13)
N1	C2	C3	C4	-84.11(10)
N1	C2	C3	C8	98.10(10)
N1	C11	C12	C13	-178.19(9)
N1	C11	C16	C15	179.10(9)
N2	C8	C3	C2	-1.48(11)
N2	C8	C3	C4	-179.28(9)
N2	C8	C7	C6	179.73(10)
C2	C3	C4	C5	-178.47(9)
C2	C3	C8	C7	178.44(10)
C3	C4	C5	C6	0.09(12)
C3	C8	C7	C6	-0.19(11)
C4	C5	C6	C7	0.38(12)
C5	C6	C7	C8	-0.33(12)
C11	C12	C13	C14	-1.10(12)
C11	C16	C15	C14	-0.68(12)
C12	C13	C14	C15	-0.21(12)
C13	C14	C15	C16	1.10(13)

Table S7 Hydrogen Fractional Atomic Coordinates (×10^4) and Equivalent Isotropic Displacement Parameters (Å^2×10^3) for **2**-C_1-Me. U_{eq} is defined as 1/3 of the trace of the orthogonalized U_{ij}.

Atom	x	y	z	U_{eq}
H2	3284(18)	5970(19)	7072(15)	41(4)
H1	4117(16)	2985(14)	4588(11)	33(3)
H2a	3008(15)	3821(13)	7748(11)	32(3)
H2b	4459(16)	2930(14)	8466(11)	31(3)
H4	7864(16)	4384(14)	9568(12)	37(3)
H5	10417(17)	6967(15)	10974(12)	43(4)
H6	9857(16)	9413(15)	11030(12)	38(3)
H7	6761(15)	9268(15)	9636(12)	36(3)
H10a	1030(20)	8350(20)	7440(18)	88(6)
H10b	685(19)	6490(20)	6566(13)	68(5)
H10c	-210(20)	6860(20)	7884(16)	92(7)
H12	7614(17)	3862(16)	5658(12)	40(4)
H13	9723(17)	2316(15)	5461(13)	47(4)
H15	6610(17)	-827(15)	7244(13)	44(4)
H16	4487(17)	643(14)	7430(12)	39(4)

Table S8 Hydrogen bond information for **2**-C_1-Me.

D	H	A	d(D-H)/Å	d(H-A)/Å	d(D-A)/Å	D-H-A/deg
N2	H2	O1	1.014(15)	1.893(16)	2.8691(12)	160.4(10)

S3. Experimental conformational free energies, ΔG_{exp} and $\Delta G_{control}$

S3.1 NMR determination of conformational free energies, ΔG_{exp} and $\Delta G_{control}$

Since rotation around the formamide bond is slow on the NMR timescale, discrete peaks corresponding to the open (unfolded) and closed (folded) conformers are observed. Thus, integration of the conformer peaks provides direct access to the conformational equilibrium constant, *K*, which can be used to determine the conformational free energy difference.

All molecular torsion balances were fully characterized in DMSO-d_6 by ^{1}H and ^{13}C-NMR, prior to the determination of experimental conformational free energies. The NMR resonances corresponding to the folded and unfolded conformers were assigned using 2D NMR methods (see Section 2.3 above). The assignment of conformer peaks was greatly simplified due to the steric differences on each side of the balance; all balances were found to prefer the folded conformation (which was supported by DFT calculations, Table S38). NMR spectra used for conformational free energy determination were determined by ^{19}F NMR spectroscopy using a Bruker Ultrashield 500 MHz, heteronuclear (512 scans). Conformer ratios were determined at balance concentrations of 3.5 mM and were found to be independent of concentration in a range of 1–4 mM (Table S9). The conformational free energy differences measured in a range of solvents are provided in Tables S9–S32.

$$\Delta G_{exp} = -RTlnK = -RTln\frac{[\text{Folded}]}{[\text{Unfolded}]}$$ **Equation S1**[S1]

Errors in conformational free energy differences (ΔG_{exp} and $\Delta G_{control}$)

Integration errors associated with NMR spectroscopy was found not to exceed 1–2% in 1998 and is even lower when using more modern spectrometers and NMR analysis software.[S3] Accordingly, a ±2% integration error (and detection threshold) in the *minor/major* peak integral ratio (i.e. ±0.02 in the *minor/major* peak integral ratio) was applied, resulting in asymmetric ΔG_{exp} error margins (listed in Tables S10–S32). This results in a conformational free energy error $\delta(\Delta G_{exp})$ for ΔG_{exp} values between −2.09 - +2.09 kJ mol^{-1} (*i.e.* conformer ratio ≥ 0.43:1) of ± 0.12 kJ mol^{-1}, in accord with prior NMR-based studies of molecular balances.[S2]

Errors in $\Delta G_{exp} - \Delta G_{control}$ were then determined as follows:

$$\delta(\Delta G_{exp} - \Delta G_{\text{control}}) = \sqrt{\left(\delta\Delta G_{exp}\right)^2 + (\delta\Delta G_{control})^2}$$ **Equation S2**

Table S9 Concentration study of molecular balances **1**-C_1-H, **2**-C_1-Me and **2**-C_1-PyrF.

**Compound**	**c / mM**	**CD_3OD ΔG_{exp} / kJ mol^{-1}**	**$CDCl_3$ ΔG_{exp}/ kJ mol^{-1}**
	1	−4.4	−6.3
**1**-C_1-H	2	−4.4	−6.3
	4	−4.4	−6.3
	1	−5.3	<−10
**2**-C_1-Me	2	−5.3	<−10
	4	−5.5	<−10
	1	−4.4	−6.6
**2**-C_1-PyrF	2	−4.4	−6.6
	4	−4.4	−6.6

Methylene (C_1) linker series

Table S10 Experimental conformational free energy differences of molecular balance **1**-C_1-H, ΔG_{exp} measured in various solvents at 298 K.Units in kJ mol^{-1}.

**1**-C_1-H						
		**Errors**		**$\Delta G_{exp} - \Delta G_{control}$**	**Errors**	
	**ΔG_{exp}**	−	+		−	+
**Chloroform-*d***	<−10[a]	-	-	<−4.9	-	-
**Acetone**	−7.4	1.2	0.8	−3.2	1.3	0.9
**Acetonitrile-*d*3**	−7.0	1.0	0.7	−3.2	1.0	0.7
**Ethyl acetate**	n.d.[b]	-	-	-	-	-
**Tetrahydrofuran**	−9.7	9.1	1.7	−4.6	9.1	1.7
**Dichloromethane**	<−10[a]	-	-	<−4.5	-	-
**Ethanol**	−7.0	1.0	0.7	−2.7	1.0	0.8
**Methanol-*d*4**	−6.6	0.8	0.6	−2.6	0.9	0.7
**DMSO-*d*6**	−5.5	0.5	0.4	−1.9	0.5	0.5

[a]Only one peak observed in NMR spectrum that was assigned to the folded conformer.

[b]Not determined due to peak overlap in the NMR spectrum.

Table S11 Experimental conformational free energy differences of molecular balance **1**-C_1-Ph, ΔG_{exp} measured in various solvents at 298 K. Units in kJ mol^{-1}.

**1**-C_1-Ph						
		**Errors**		**$\Delta G_{exp} - \Delta G_{control}$**	**Errors**	
	**ΔG_{exp}**	−	+		−	+
**Chloroform-*d***	<−10[a]	-	-	<-4.9	-	-
**Acetone**	−6.6	0.8	0.6	−2.3	0.9	0.7
**Acetonitrile-*d*3**	−6.6	0.8	0.6	−2.8	0.9	0.7
**Ethyl acetate**	−8.7	2.6	1.3	−3.6	2.7	1.3
**Tetrahydrofuran**	n.d.[b]	-	-	−4.9	-	-
**Dichloromethane**	<−10[a]	-	-	<−4.5	-	-
**Ethanol**	−6.0	0.6	0.5	−1.7	0.7	0.6
**Methanol-*d*4**	−5.1	0.4	0.4	−1.1	0.5	0.4
**DMSO-*d*6**	−4.4	0.3	0.3	−0.9	0.4	0.3

[a]Only one peak observed in NMR spectrum that was assigned to the folded conformer.

[b]Not determined due to peak overlap in the NMR spectrum.

Table S12 Experimental conformational free energy differences of molecular balance **1**-C_1-PhOMe, ΔG_{exp} measured in various solvents at 298 K. Units in kJ mol^{-1}.

**1**-C_1-PhOMe						
		**Errors**		**$\Delta G_{exp} - \Delta G_{control}$**	**Errors**	
	**ΔG_{exp}**	−	+		−	+
**Chloroform-*d***	−6.5	0.8	0.6	−1.4	0.9	0.7
**Acetone**	−5.7	0.5	0.4	−1.5	0.6	0.5
**Acetonitrile-d_3**	−5.5	0.5	0.4	−1.7	0.5	0.5
**Ethyl acetate**	−6.3	0.7	0.5	−1.2	0.8	0.7
**Tetrahydrofuran**	−6.6	0.8	0.6	−1.5	0.9	0.7
**Dichloromethane**	−6.3	0.7	0.5	−0.8	0.9	0.7
**Ethanol**	−5.1	0.4	0.4	−0.8	0.5	0.4
**Methanol-d_4**	−4.4	0.3	0.3	−0.4	0.4	0.4
**DMSO-d_6**	−4.4	0.3	0.3	−0.9	0.4	0.3

Table S13 Experimental conformational free energy differences of molecular balance **1**-C_1-PhCN, ΔG_{exp} measured in various solvents at 298 K. Units in kJ mol^{-1}.

**1**-C_1-PhCN						
		**Errors**		**$\Delta G_{exp} - \Delta G_{control}$**	**Errors**	
	**ΔG_{exp}**	−	+		−	+
**Chloroform-*d***	−8.7	2.6	1.3	−3.6	2.7	1.3
**Acetone**	−6.3	0.7	0.5	−2.0	0.8	0.6
**Acetonitrile-d_3**	−5.7	0.5	0.4	−2.0	0.6	0.5
**Ethyl acetate**	−7.0	1.0	0.7	−1.9	1.1	0.8
**Tetrahydrofuran**	−7.0	1.0	0.7	−1.9	1.1	0.8
**Dichloromethane**	−8.0	1.7	1.0	−2.5	1.7	1.1
**Ethanol**	−5.7	0.5	0.4	−1.5	0.6	0.5
**Methanol-d_4**	−5.3	0.4	0.4	−1.3	0.5	0.4
**DMSO-d_6**	−4.4	0.3	0.3	−0.9	0.4	0.3

Table S14 Experimental conformational free energy differences of molecular balance **2**-C_1-Me, ΔG_{exp} measured in various solvents at 298 K. Units in kJ mol^{-1}.

**2**-C_1-Me						
		**Errors**		**$\Delta G_{exp} - \Delta G_{control}$**	**Errors**	
	**ΔG_{exp}**	−	+		−	+
**Chloroform-*d***	<−10[a]	-	-	<−4.9	-	-
**Acetone**	−8.0	1.7	1.0	−3.7	1.7	1.0
**Acetonitrile-*d*$_3$**	−7.0	1.0	0.7	−3.2	1.0	0.7
**Ethyl acetate**	−9.7	9.1	1.7	−4.6	9.1	1.7
**Tetrahydrofuran**	−9.7	9.1	1.7	−4.6	9.1	1.7
**Dichloromethane**	<−10[a]	-	-	<−4.5	-	-
**Ethanol**	−6.0	0.6	0.5	−1.7	0.7	0.6
**Methanol-*d*$_4$**	−5.5	0.5	0.4	−1.5	0.6	0.5
**DMSO-*d*$_6$**	−4.5	0.3	0.3	−1.0	0.4	0.4

Table S15 Experimental conformational free energy differences of molecular balance **2**-C_1-CF_3, ΔG_{exp} measured in various solvents at 298 K. Units in kJ mol^{-1}.

**2**-C_1-CF_3						
		**Errors**		**$\Delta G_{exp} - \Delta G_{control}$**	**Errors**	
	**ΔG_{exp}**	−	+		−	+
**Chloroform-*d***	<−10[a]	-	-	<−4.9	-	-
**Acetone**	−8.7	2.6	1.3	−4.4	2.6	1.3
**Acetonitrile-*d*$_3$**	−8.0	1.7	1.0	−4.2	1.7	1.0
**Ethyl acetate**	−9.7	9.1	1.7	−4.6	9.1	1.7
**Tetrahydrofuran**	−9.7	9.1	1.7	−4.6	9.1	1.7
**Dichloromethane**	<−10[a]	-	-	<−4.5	-	-
**Ethanol**	−6.3	0.7	0.5	−2.0	0.8	0.6
**Methanol-*d*$_4$**	−5.7	0.5	0.4	−1.7	0.6	0.5
**DMSO-*d*$_6$**	−4.9	0.4	0.3	-1.3	0.4	0.4

[a]Only one peak observed in NMR spectrum that was assigned to the folded conformer.

Table S16 Experimental conformational free energy differences of molecular balance **2**-C_1-^tBu, ΔG_{exp} measured in various solvents at 298 K. Units in kJ mol^{-1}.

**2**-C_1-^tBu						
		**Errors**		**ΔG_{exp} – $\Delta G_{control}$**	**Errors**	
	**ΔG_{exp}**	–	+		–	+
**Chloroform-*d***	<−10[a]	-	-	<−4.9	-	-
**Acetone**	−7.4	1.2	0.8	−3.2	1.3	0.9
**Acetonitrile-*d*$_3$**	−6.3	0.7	0.5	−2.5	0.7	0.6
**Ethyl acetate**	−9.7	9.1	1.7	−4.6	9.1	1.7
**Tetrahydrofuran**	−8.7	2.6	1.3	−3.6	2.7	1.3
**Dichloromethane**	<−10[a]	-	-	<−4.5	-	-
**Ethanol**	−6.3	0.7	0.5	−2.0	0.8	0.6
**Methanol-*d*$_4$**	−5.5	0.5	0.4	−1.5	0.6	0.5
**DMSO-*d*$_6$**	−4.7	0.4	0.3	−1.2	0.4	0.4

[a]Only one peak observed in NMR spectrum that was assigned to the folded conformer.

Table S17 Experimental conformational free energy differences of molecular balance **2**-C_1-Ph, ΔG_{exp} measured in various solvents at 298 K. Units in kJ mol^{-1}.

**2**-C_1-Ph						
		**Errors**		**ΔG_{exp} – $\Delta G_{control}$**	**Errors**	
	**ΔG_{exp}**	–	+		–	+
**Chloroform-*d***	<−10[a]	-	-	<−4.9	-	-
**Acetone**	−7.4	1.2	0.8	−3.2	1.3	0.9
**Acetonitrile-*d*$_3$**	−7.0	1.0	0.7	−3.2	1.0	0.7
**Ethyl acetate**	−9.7	9.1	1.7	−4.6	9.1	1.7
**Tetrahydrofuran**	−8.7	2.6	1.3	−3.6	2.7	1.3
**Dichloromethane**	<−10[a]	-	-	<−4.5	-	-
**Ethanol**	−6.0	0.6	0.5	−1.7	0.7	0.6
**Methanol-*d*$_4$**	−5.3	0.4	0.4	−1.3	0.5	0.4
**DMSO-*d*$_6$**	−4.5	0.3	0.3	−1.0	0.4	0.4

[a]Only one peak observed in NMR spectrum that was assigned to the folded conformer.

Table S18 Experimental conformational free energy differences of molecular balance **2**-C_1-PhOMe, ΔG_{exp} measured in various solvents at 298 K. Units in kJ mol^{-1}.

**2**-C_1-PhOMe						
		**Errors**		**$\Delta G_{exp} - \Delta G_{control}$**	**Errors**	
	**ΔG_{exp}**	−	+		−	+
**Chloroform-*d***	<−10[a]	-	-	<−4.9	-	-
**Acetone**	−7.4	1.2	0.8	−3.2	1.3	0.9
**Acetonitrile-*d*$_3$**	−7.0	1.0	0.7	−3.2	1.0	0.7
**Ethyl acetate**	−9.7	9.1	1.7	−4.6	9.1	1.7
**Tetrahydrofuran**	−8.7	2.6	1.3	−3.6	2.7	1.3
**Dichloromethane**	<−10[a]	-	-	<−4.5	-	-
**Ethanol**	−5.7	0.6	0.5	−1.7	0.7	0.6
**Methanol-*d*$_4$**	−5.3	0.4	0.4	−1.3	0.5	0.4
**DMSO-*d*$_6$**	−4.5	0.3	0.3	−1.0	0.4	0.4

[a]Only one peak observed in NMR spectrum that was assigned to the folded conformer.

Table S19 Experimental conformational free energy differences of molecular balance **2**-C_1-Pyr, ΔG_{exp} measured in various solvents at 298 K. Units in kJ mol^{-1}.

**2**-C_1-Pyr						
		**Errors**		**$\Delta G_{exp} - \Delta G_{control}$**	**Errors**	
	**ΔG_{exp}**	−	+		−	+
**Chloroform-*d***	−6.0	0.6	0.5	−0.9	0.7	0.6
**Acetone**	−4.5	0.3	0.3	−0.3	0.4	0.4
**Acetonitrile-*d*$_3$**	−4.4	0.3	0.3	−0.6	0.4	0.3
**Ethyl acetate**	−5.3	0.4	0.4	−0.2	0.6	0.5
**Tetrahydrofuran**	−5.3	0.4	0.4	−0.2	0.6	0.5
**Dichloromethane**	−5.5	0.5	0.4	0.0	0.7	0.6
**Ethanol**	−4.5	0.3	0.3	−0.3	0.4	0.4
**Methanol-*d*$_4$**	−3.9	0.2	0.2	0.1	0.4	0.3
**DMSO-*d*$_6$**	−3.9	0.2	0.2	−0.3	0.3	0.3

Table S20 Experimental conformational free energy differences of molecular balance **2**-C_1-PyrF, ΔG_{exp} measured in various solvents at 298 K. Units in kJ mol^{-1}.

**2**-C_1-PyrF						
		**Errors**		$\Delta G_{exp} - \Delta G_{control}$	**Errors**	
	ΔG_{exp}	−	+		−	+
**Chloroform-*d***	−6.6	0.8	0.6	−1.5	0.9	0.7
**Acetone**	−4.9	0.4	0.3	−0.6	0.5	0.4
**Acetonitrile-*d*$_3$**	−4.8	0.4	0.3	−1.1	0.4	0.4
**Ethyl acetate**	−5.7	0.5	0.4	−0.7	0.7	0.6
**Tetrahydrofuran**	−5.5	0.5	0.4	−0.4	0.6	0.5
**Dichloromethane**	−6.0	0.6	0.5	−0.5	0.8	0.6
**Ethanol**	−4.9	0.4	0.3	−0.6	0.5	0.4
**Methanol-*d*$_4$**	−4.2	0.3	0.3	−0.3	0.4	0.4
**DMSO-*d*$_6$**	−4.0	0.3	0.2	−0.5	0.3	0.3

Ethylene (C_2) linker series

Table S21 Experimental conformational free energy differences of molecular balance **1**-C_2-H, ΔG_{exp} measured in various solvents at 298 K. Units in kJ mol^{-1}.

**1**-C_2-H						
		**Errors**		$\Delta G_{exp} - \Delta G_{control}$	**Errors**	
	ΔG_{exp}	−	+		−	+
**Chloroform-*d***	−7.4	1.2	0.8	−2.9	1.3	0.9
**Acetone**	−5.5	0.5	0.4	−1.9	0.5	0.5
**Acetonitrile-*d*$_3$**	−4.7	0.4	0.3	−1.7	0.4	0.3
**Ethyl acetate**	−6.6	0.8	0.6	−2.2	0.9	0.7
**Tetrahydrofuran**	−6.6	0.8	0.6	−1.9	0.9	0.7
**Dichloromethane**	−7.0	1.0	0.7	−3.0	1.0	0.7
**Ethanol**	−4.5	0.3	0.3	−1.0	0.4	0.3
**Methanol-*d*$_4$**	−4.1	0.3	0.2	−1.0	0.3	0.3
**DMSO-*d*$_6$**	−3.5	0.2	0.2	−0.7	0.3	0.2

Table S22 Experimental conformational free energy differences of molecular balance **1**-C_2-Ph, ΔG_{exp} measured in various solvents at 298 K. Units in kJ mol^{-1}.

**1**-C_2-Ph						
		**Errors**		$\Delta G_{exp} - \Delta G_{control}$	**Errors**	
	ΔG_{exp}	−	+		−	+
**Chloroform-*d***	−8.0	1.7	1.0	−3.5	1.7	1.0
**Acetone**	−5.5	0.5	0.4	−1.9	0.5	0.5
**Acetonitrile-*d*$_3$**	−4.7	0.4	0.3	−1.7	0.4	0.3
**Ethyl acetate**	−7.0	1.0	0.7	−2.6	1.0	0.8
**Tetrahydrofuran**	−7.0	1.0	0.7	−2.3	1.0	0.8
**Dichloromethane**	−7.0	1.0	0.7	−3.0	1.0	0.7
**Ethanol**	−4.4	0.3	0.3	−0.9	0.4	0.3
**Methanol-*d*$_4$**	−3.9	0.2	0.2	−0.8	0.3	0.3
**DMSO-*d*$_6$**	−3.4	0.2	0.2	−0.6	0.3	0.2

Table S23 Experimental conformational free energy differences of molecular balance **1**-C_2-PhOMe, ΔG_{exp} measured in various solvents at 298 K. Units in kJ mol^{-1}.

1-C_2-PhOMe

		Errors		$\Delta G_{exp} - \Delta G_{control}$	Errors	
	ΔG_{exp}	−	+		−	+
**Chloroform-*d***	−5.1	0.4	0.4	−0.6	0.5	0.5
**Acetone**	−4.5	0.3	0.3	−0.9	0.4	0.4
**Acetonitrile-*d*$_3$**	−3.8	0.2	0.2	−0.8	0.3	0.3
**Ethyl acetate**	−5.1	0.4	0.4	−0.7	0.5	0.4
**Tetrahydrofuran**	−5.3	0.4	0.4	−0.6	0.6	0.5
**Dichloromethane**	−4.7	0.4	0.3	−0.7	0.4	0.4
**Ethanol**	−4.0	0.3	0.2	−0.5	0.3	0.3
**Methanol-*d*$_4$**	−3.4	0.2	0.2	−0.3	0.3	0.3
**DMSO-*d*$_6$**	−3.2	0.2	0.2	−0.4	0.2	0.2

Table S24 Experimental conformational free energy differences of molecular balance **1**-C_2-PhCN, ΔG_{exp} measured in various solvents at 298 K. Units in kJ mol^{-1}.

1-C_2-PhCN

		Errors		$\Delta G_{exp} - \Delta G_{control}$	Errors	
	ΔG_{exp}	−	+		−	+
**Chloroform-*d***	−6.0	0.6	0.5	−1.5	0.7	0.6
**Acetone**	−4.7	0.4	0.3	−1.1	0.4	0.4
**Acetonitrile-*d*$_3$**	−3.9	0.2	0.2	−0.9	0.3	0.3
**Ethyl acetate**	n.d.[a]	-	-	-	-	-
**Tetrahydrofuran**	−5.3	0.4	0.4	−0.6	0.6	0.5
**Dichloromethane**	−5.7	0.5	0.4	−1.7	0.6	0.5
**Ethanol**	−4.4	0.3	0.3	−0.9	0.4	0.3
**Methanol-*d*$_4$**	−3.8	0.2	0.2	−0.7	0.3	0.3
**DMSO-*d*$_6$**	−3.2	0.2	0.2	−0.4	0.2	0.2

[a]Not determined due to peak overlap in the NMR spectrum.

Table S25 Experimental conformational free energy differences of molecular balance **2**-C_2-Me, ΔG_{exp} measured in various solvents at 298 K. Units in kJ mol^{-1}.

2-C_2-Me

		Errors		$\Delta G_{exp} - \Delta G_{control}$	Errors	
	ΔG_{exp}	−	+		−	+
**Chloroform-*d***	<−10[a]	-	-	<−5.5	-	-
**Acetone**	−6.0	0.6	0.5	−2.4	0.7	0.5
**Acetonitrile-*d*$_3$**	−5.1	0.4	0.4	−2.1	0.4	0.4
**Ethyl acetate**	−8.7	2.6	1.3	−4.3	2.6	1.3
**Tetrahydrofuran**	−8.7	2.6	1.3	−4.0	2.7	1.3
**Dichloromethane**	<−10[a]	-	-	<−6.0	10.0	0.0
**Ethanol**	−4.9	0.4	0.3	−1.4	0.4	0.4
**Methanol-*d*$_4$**	−4.4	0.3	0.3	−1.3	0.4	0.3
**DMSO-*d*$_6$**	−3.5	0.2	0.2	−0.7	0.3	0.2

[a]Only one peak observed in NMR spectrum that was assigned to the folded conformer.

Table S26 Experimental conformational free energy differences of molecular balance **2**-C_2-CF_3, ΔG_{exp} measured in various solvents at 298 K. Units in kJ mol^{-1}.

2-C_2-CF_3

		Errors		$\Delta G_{exp} - \Delta G_{control}$	Errors	
	ΔG_{exp}	−	+		−	+
**Chloroform-*d***	<−10[a]	-	-	<−5.5	10.0	0.0
**Acetone**	−5.1	0.4	0.4	−1.5	0.5	0.4
**Acetonitrile-*d*$_3$**	−4.5	0.3	0.3	−1.5	0.4	0.3
**Ethyl acetate**	−6.3	0.7	0.5	−1.9	0.8	0.6
**Tetrahydrofuran**	−6.3	0.7	0.5	−1.6	0.8	0.6
**Dichloromethane**	<−10[a]	-	-	<−6.0	-	-
**Ethanol**	−4.4	0.3	0.3	−0.9	0.4	0.3
**Methanol-*d*$_4$**	−3.8	0.2	0.2	−0.7	0.3	0.3
**DMSO-*d*$_6$**	−3.4	0.2	0.2	−0.6	0.3	0.2

[a]Only one peak observed in NMR spectrum that was assigned to the folded conformer.

Table S27 Experimental conformational free energy differences of molecular balance **2**-C_2-^tBu, ΔG_{exp} measured in various solvents at 298 K. Units in kJ mol^{-1}.

2-C_2-^tBu

		Errors		$\Delta G_{exp} - \Delta G_{control}$	Errors	
	ΔG_{exp}	−	+		−	+
**Chloroform-*d***	−7.4	1.2	0.8	−2.9	1.3	0.9
**Acetone**	−4.5	0.3	0.3	−0.9	0.4	0.4
**Acetonitrile-d_3**	−3.9	0.2	0.2	−0.9	0.3	0.3
**Ethyl acetate**	−6.3	0.7	0.5	−1.9	0.8	0.6
**Tetrahydrofuran**	−6.3	0.7	0.5	−1.6	0.8	0.6
**Dichloromethane**	−7.0	1.0	0.7	−3.0	1.0	0.7
**Ethanol**	−4.4	0.3	0.3	−0.9	0.4	0.3
**Methanol-d_4**	−3.9	0.2	0.2	−0.8	0.3	0.3
**DMSO-d_6**	−3.1	0.2	0.2	−0.3	0.2	0.2

Table S28 Experimental conformational free energy differences of molecular balance **2**-C_2-Ph, ΔG_{exp} measured in various solvents at 298 K. Units in kJ mol^{-1}.

2-C_2-Ph

		Errors		$\Delta G_{exp} - \Delta G_{control}$	Errors	
	ΔG_{exp}	−	+		−	+
**Chloroform-*d***	−8.0	1.7	1.0	-3.5	1.7	1.0
**Acetone**	−4.7	0.4	0.3	-1.1	0.4	0.4
**Acetonitrile-d_3**	−4.4	0.3	0.3	-1.4	0.4	0.3
**Ethyl acetate**	−6.0	0.6	0.5	-1.6	0.7	0.6
**Tetrahydrofuran**	−6.0	0.6	0.5	-1.3	0.7	0.6
**Dichloromethane**	−7.4	1.2	0.8	-3.4	1.3	0.9
**Ethanol**	−4.1	0.3	0.2	-0.6	0.3	0.3
**Methanol-d_4**	−4.7	0.2	0.2	-0.7	0.3	0.3
**DMSO-d_6**	−3.2	0.2	0.2	-0.4	0.2	0.2

Table S29 Experimental conformational free energy differences of molecular balance **2**-C_2-PhOMe, ΔG_{exp} measured in various solvents at 298 K. Units in kJ mol^{-1}.

2-C_2-PhOMe

	ΔG_{exp}	Errors −	Errors +	$\Delta G_{exp} - \Delta G_{control}$	Errors −	Errors +
**Chloroform-*d***	−4.2	1.7	1.0	−3.5	1.7	1.0
**Acetone**	−4.0	0.3	0.3	−0.6	0.4	0.3
**Acetonitrile-*d*$_3$**	−6.0	0.3	0.2	−1.0	0.3	0.3
**Ethyl acetate**	−6.3	0.6	0.5	−1.6	0.7	0.6
**Tetrahydrofuran**	−7.0	0.7	0.5	−1.6	0.8	0.6
**Dichloromethane**	−4.1	1.0	0.7	−3.0	1.0	0.7
**Ethanol**	−3.8	0.3	0.2	−0.6	0.3	0.3
**Methanol-*d*$_4$**	−3.1	0.2	0.2	−0.7	0.3	0.3
**DMSO-*d*$_6$**	−4.2	0.2	0.2	−0.3	0.2	0.2

Table S30 Experimental conformational free energy differences of molecular balance **2**-C_2-Pyr, ΔG_{exp} measured in various solvents at 298 K. Units in kJ mol^{-1}.

2-C_2-Pyr

	ΔG_{exp}	Errors −	Errors +	$\Delta G_{exp} - \Delta G_{control}$	Errors −	Errors +
**Chloroform-*d***	−4.9	0.4	0.3	-0.4	0.5	0.4
**Acetone**	−4.0	0.3	0.2	-0.4	0.3	0.3
**Acetonitrile-*d*$_3$**	−3.4	0.2	0.2	-0.4	0.3	0.2
**Ethyl acetate**	n.d.[a]	-	-	-	-	-
**Tetrahydrofuran**	−4.7	0.4	0.3	0.0	0.5	0.4
**Dichloromethane**	−4.4	0.3	0.3	-0.4	0.4	0.4
**Ethanol**	−4.1	0.3	0.2	-0.6	0.3	0.3
**Methanol-*d*$_4$**	−3.6	0.2	0.2	-0.5	0.3	0.3
**DMSO-*d*$_6$**	−3.1	0.2	0.2	-0.3	0.2	0.2

[a]Only one peak observed in NMR spectrum that was assigned to the folded conformer.

Table S31 Experimental conformational free energy differences of molecular balance **2**-C_2-PyrF, ΔG_{exp} measured in various solvents at 298 K. Units in kJ mol^{-1}.

2-C_2-PyrF

		**Errors**		**ΔG_{exp} – $\Delta G_{control}$**	**Errors**	
	**ΔG_{exp}**	−	+		−	+
**Chloroform-*d***	−5.3	0.4	0.4	−0.8	0.6	0.5
**Acetone**	−4.1	0.3	0.2	−0.5	0.4	0.3
**Acetonitrile-*d*$_3$**	−3.5	0.2	0.2	−0.5	0.3	0.3
**Ethyl acetate**	−5.1	0.4	0.4	−0.7	0.5	0.4
**Tetrahydrofuran**	−5.3	0.4	0.4	−0.6	0.6	0.5
**Dichloromethane**	−4.7	0.4	0.3	−0.7	0.4	0.4
**Ethanol**	−4.2	0.3	0.3	−0.7	0.4	0.3
**Methanol-*d*$_4$**	−3.8	0.2	0.2	−0.7	0.3	0.3
**DMSO-*d*$_6$**	−3.1	0.2	0.2	−0.3	0.2	0.2

Control series

Table S32 Experimental conformational free energy differences of the **Control**-C_1 and **Control**-C_2 molecular balances, $\Delta G_{control}$ measured in various solvents at 298 K. Units in kJ mol^{-1}.

Control-C_1

		Error	
	**$\Delta G_{control}$**	−	+
**Chloroform-*d***	−5.1	0.4	0.4
**Acetone**	−4.2	0.3	0.3
**Acetonitrile-d_3**	−3.8	0.2	0.2
**Ethyl acetate**	−5.1	0.4	0.4
**Tetrahydrofuran**	−5.1	0.4	0.4
**Dichloromethane**	−5.5	0.5	0.4
**Ethanol**	−4.2	0.3	0.3
**Methanol-d_4**	−4.0	0.3	0.2
**DMSO-d_6**	−3.5	0.2	0.2

Control-C_2

		Error	
	**$\Delta G_{control}$**	−	+
**Chloroform-*d***	−4.5	0.3	0.3
**Acetone**	−3.6	0.2	0.2
**Acetonitrile-d_3**	−3.0	0.2	0.2
**Ethyl acetate**	−4.4	0.3	0.3
**Tetrahydrofuran**	−4.7	0.4	0.3
**Dichloromethane**	−4.0	0.3	0.2
**Ethanol**	−3.5	0.2	0.2
**Methanol-d_4**	−3.1	0.2	0.2
**DMSO-d_6**	−2.8	0.2	0.1

S3.3 Van't Hoff analyses of compounds 2-C_1-Ph and 2-C_2-Ph

Van't Hoff analysis was carried out on ~3.5 mM samples of compounds **2**-C_1-Ph and **2**-C_2-Ph in DMSO-*d_6*. Samples were prepared and placed in an air-tight Wilmad-cap NMR tube. Spectra were obtained at a minimum of seven temperatures, beginning with the coldest. Samples were equilibrated at each temperature for 30 minutes within the spectrometer. Results are shown in Table S33, S34 and Figure S45 showing the derivation of thermodynamic parameters from the gradient and intercept according to the equation $\Delta G = \Delta H - T\Delta S$.

Favourable entropy contributions were observed for both balances, which is consistent with release of (H-bonded) solvent molecules into the bulk upon formation of the intramolecular H-bond. However, the non-linear temperature dependencies of the van't Hoff analyses meant that quantitative comparisons between the methylene and ethylene linked series could not be made.

Table S33 Van't Hoff analysis of **2**-C_1-Ph and **2**-C_2-Ph.

		**2**-C_1-Ph	**2**-C_2-Ph
***T*/ K**	**1/*T***	***K***	***K***
300	0.00333	1.845	1.277
305	0.00328	1.808	1.273
310	0.00323	1.766	1.266
315	0.00317	1.760	1.259
320	0.00313	1.772	1.248
325	0.00308	one peak	1.241
330	0.00303	one peak	1.245

Table S34 Dissection of ΔH and $T\Delta S$ at 300 K for **2**-C_1-Ph and **2**-C_2-Ph.

Balance	$-T\Delta S$/ kJ mol^{-1}	ΔH/ kJ mol^{-1}	ΔG/ kJ mol^{-1}
**2**-C_1-Ph	−1.5 (+1.1, −1.2)	−3.1 ± 1.0	−4.6 (+0.4, −0.5)
**2**-C_2-Ph	−2.1 ± 0.3	−1.0 ± 0.1	−3.2 ± 0.3

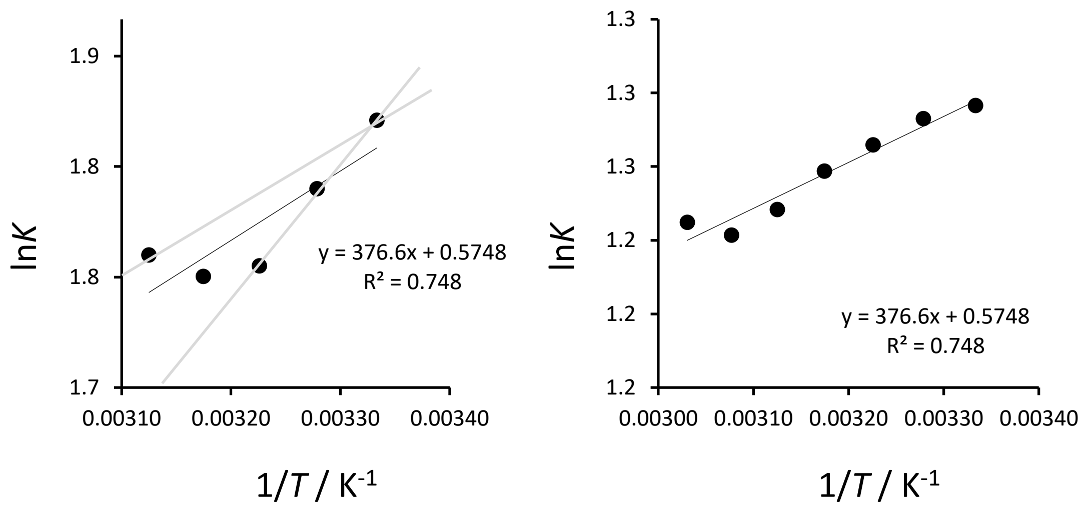

Figure S45 Van't Hoff analysis for compounds **1**-C_1-Ph (left) and **1**-C_2-Ph (right).

S3.4 Dissection of interaction energies using Hunter's solvation model

The data can be further analysed using the Hunter solvation model[S1, S4] (Figure S46 and Equation S3), where α_s and β_s describe the H-bond donor and acceptor ability of the solvent, respectively.

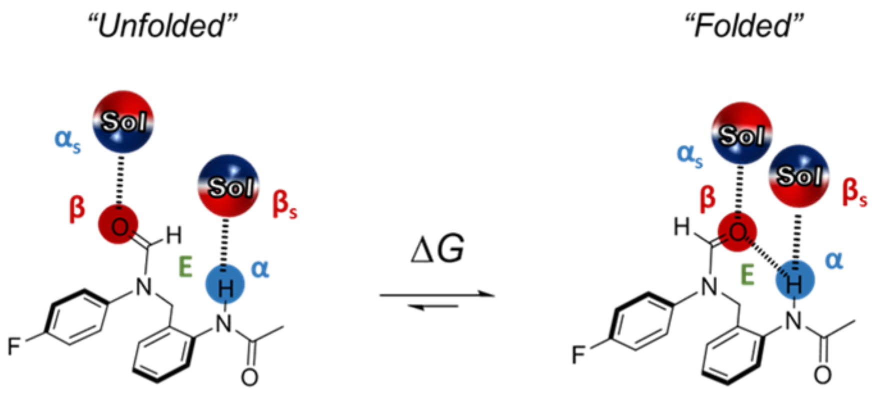

Figure S46 Hunter's solvation model adapted for the formamide balances.[S1, S4] E_{fold} and E_{unfold} correspond to intramolecular interactions in the folded and unfolded conformers, respectively. α_s and β_s are the H-bond donor and acceptor constants of the solvent, respectively. α_{fold} and β_{fold} correspond to H-bond donor and acceptor constants of the folded conformer, respectively. α_{unfold} and β_{unfold} correspond to H-bond donor and acceptor constants of the unfolded conformer, respectively.

Equation S3 describes the Hunter solvation model as adapted for formamide balances.

$$\Delta G_{\alpha/\beta\ model} = E_{fold} - E_{unfold} + \alpha_s(\beta_{unfold} - \beta_{fold}) + \beta_s(\alpha_{unfold} - \alpha_{fold})$$

$$= \Delta E + \alpha_s \Delta\beta + \beta_s \Delta\alpha \qquad \textbf{Equation S3}$$

ΔE_{HB} is the intramolecular H-bond (*i.e.* dominated by the H-bond for these balances), and $\Delta\alpha$ and $\Delta\beta$ are the differences in the H-bond donor and acceptor constants between the folded and unfolded conformers (understood globally for each conformer, i.e. Boltzmann-averaged).

Assumptions and considerations: Note that the entropic constant that is normally included for intermolecular complexes in the Hunter model is not included for this intramolecular system, since only a single formyl C-N bond rotation is required to form the intramolecular H-bond.[S1] In addition, the solvophobic term $\alpha_s.\beta_s$ that is normally included in the Hunter solvation model is not included.[S1, S7a, S7b]

Equation S3 can be modified to include solvophobic effects by including the $-\ \alpha_s\beta_s$ term as follows:

$$\Delta G_{\alpha/\beta\ model} = \Delta E + \alpha_s \Delta\beta + \beta_s \Delta\alpha - \ \alpha_s\beta_s$$

$$\Delta G_{\alpha/\beta\ model} = \Delta E + \alpha_s(\Delta\beta - \beta_s) + \beta_s(\Delta\alpha - \ \alpha_s)$$

Hence, the occurrence of solvophobic effects, but still fitting to Equation S3 (without the solvophobic term included) would result in the apparent $\Delta\alpha$ and $\Delta\beta$ (solvent-sensitivity) terms being underestimated relative to ΔE.

We have previously found that including a solvophobic term did not improve the dissection of solvent effects in closely related formamide molecular balances.[S1] Nonetheless, regression fitting for the present work was initially attempted including the $\alpha_s.\beta_s$ term, but this was not found to significantly improve the correlations. Moreover, the fitting (uncertainty) associated with the output coefficients $\Delta E, \Delta\beta$ and $\Delta\alpha$ were substantially increased upon inclusion of the solvophobic $\alpha_s.\beta_s$ term. Contrasting with previous work with Wilcox molecular balances,[S10] exchange between the folded and unfolded conformation in these balances only involves the rotation of a formyl group and results in a negligible change in solvent accessible area. This conformational change is unlikely to be associated with large changes in solvent cavitation or to displace solvents into the bulk. Instead, it is more likely that there will be a more subtle restructuring of the solvent around the molecular balance upon conformational exchange, Furthermore, any residual entropic of solvophobic contributions affecting the conformation of the balances that is constant between ΔG_{exp} and $\Delta G_{control}$ would be cancelled upon subtraction of the energy terms from one another prior to the regression fitting performed as described below. Nonetheless, we cannot fully exclude the possibility of solvophobic contributions being encoded within the apparent $\Delta\beta$ and $\Delta\alpha$ values, which would manifest in these estimated solvent-sensitivities being underestimated (since solvophobic effects would provide a favourable energetic contribution).

Data fitting: Multiple linear regression was performed for each balance across all solvents exampled in *Microsoft Excel* using the Regression tool provided in the Data Analysis tool pack. For regression fitting presented in the main text, the $\Delta G_{exp} - \Delta G_{control}$ values measured for each balance across the range of solvents examined was set as the Y-variable and the corresponding α_s and β_s values for each solvent set as the X variables (listed in Table S35). The output coefficients from the linear regression ΔE (intercept), $\Delta\alpha$ (X variable 2) and $\Delta\beta$ (X variable 1) are listed in Table 1 in the main text.

We point out that prior to performing the regression fitting outlined above, initial linear regressions excluded data obtained at our defined 2% unfolded/folded NMR integration cut-off (*i.e.* where ΔG_{exp} <–10 kJ/mol). However, we found that the extrapolated $\Delta G_{\alpha/\beta\,model}$ values for the excluded data points after fitting were within ±2 kJ/mol of –10 kJ/mol (and often within ±1 kJ/mol). Hence, subsequent regression fitting included all data, including where ΔG_{exp} <–10 kJ/mol. Moreover, when we took this more inclusive approach, the quality of correlations (and the associated fitting errors) presented throughout the manuscript improved compared to excluding these data points. The implication is that while the ΔG_{exp} <–10 kJ/mol values technically represent the lower limit of the actual values, the ΔG_{exp} values obtained in the present study were in fact close to –10 kJ/mol. However, such an inclusive approach may not be universally applicable to future studies and would need to be assessed on a case-by-case basis (by fitting with and without data obtained at the limits of integration accuracy, as outlined above).

Errors in $\Delta G_{\alpha/\beta\ \mathrm{model}}$ ($\delta\Delta G_{\alpha/\beta\ \mathrm{model}}$) are listed in Table S36 and were calculated as follows:

$$\delta\Delta G_{\alpha/\beta\ \mathrm{model}} = \sqrt{(\delta\Delta E)^2 + (\delta\Delta\alpha.\beta_s)^2 + (\delta\Delta\beta.\alpha_s)^2}$$ **Equation S4**

where $\delta\Delta E$, $\delta\Delta\alpha$ and $\delta\Delta\beta$ are the standard errors in the ΔE, $\Delta\alpha$ and $\Delta\beta$ coefficients output by the Regression tool from the Data Analysis tool pack in *Excel*.

Table S35 Solvent α_s and β_s H-bond constants used in linear regression.

Solvent	α_s	β_s
Chloroform	2.2	0.9
Acetone	1.5	5.8
Acetonitrile	1.7	5.1
Ethyl acetate	1.5	5.3
THF	0.9	5.9
CH_2Cl_2	1.9	1.1
Ethanol	2.7	5.3
Methanol	2.7	5.3
DMSO	2.2	8.7

Table S36 Fitted energies $\Delta G_{\alpha/\beta\ model}$ for methylene (**1**-C_1-X and **2**-C_1-Y) and ethylene (**1**-C_2-X and **2**-C_2-Y) molecular balance series determined by fitting experimental $\Delta G_{exp} - \Delta G_{control}$ values to Equation S3. Errors in $\Delta G_{exp} - \Delta G_{control}$ are listed in Tables S28-S32. Units in kJ mol^{-1}.

Compound	Solvent	$\Delta G_{exp} - \Delta G_{control}$	$\Delta G_{\alpha/\beta\ model}$	$\delta\Delta G_{\alpha/\beta\ model}$
**1**-C_1-H	**Chloroform-*d***	<–4.9[a]	–4.6	0.8
	**Acetone**	–3.2	–3.6	0.8
	**Acetonitrile-*d*$_3$**	–3.2	–3.6	0.8
	**Ethyl acetate**	n.d.[b]	–3.7	0.8
	**Tetrahydrofuran**	–4.6	–4.1	0.7
	**Dichloromethane**	<–4.5[a]	–4.8	0.8
	**Ethanol**	–2.7	–2.6	1.0
	**Methanol-*d*$_4$**	–2.6	–2.6	1.0
	**DMSO-*d*$_6$**	–1.9	–1.9	1.0
**1**-C_1-Ph	**Chloroform-*d***	<–4.9[a]	–4.4	1.2
	**Acetone**	–2.3	–3.3	1.1
	**Acetonitrile-*d*$_3$**	–2.8	–3.2	1.1
	**Ethyl acetate**	–3.6	–3.5	1.1
	**Tetrahydrofuran**	<–4.9[a]	–4.3	1.1
	**Dichloromethane**	<–4.5[a]	–4.9	1.1
	**Ethanol**	–1.7	–1.4	1.4
	**Methanol-*d*$_4$**	–1.1	–1.4	1.4
	**DMSO-*d*$_6$**	–0.9	–0.6	1.4
**1**-C_1-PhOMe	**Chloroform-*d***	–1.4	–1.1	0.7
	**Acetone**	–1.5	–1.3	0.7
	**Acetonitrile-*d*$_3$**	–1.7	–1.2	0.7
	**Ethyl acetate**	–1.2	–1.4	0.6
	**Tetrahydrofuran**	–1.5	–1.7	0.6
	**Dichloromethane**	–0.8	–1.2	0.6
	**Ethanol**	–0.8	–0.7	0.8
	**Methanol-*d*$_4$**	–0.4	–0.7	0.8
	**DMSO-*d*$_6$**	–0.9	–0.9	0.8
**1**-C_1-PhCN	**Chloroform-*d***	–3.6	–3.0	0.7
	**Acetone**	–2.0	–1.8	0.7
	**Acetonitrile-*d*$_3$**	–2.0	v1.9	0.7
	**Ethyl acetate**	–1.9	–2.0	0.7
	**Tetrahydrofuran**	–1.9	–2.0	0.7
	**Dichloromethane**	–2.5	–3.0	0.7
	**Ethanol**	–1.5	–1.5	0.9
	**Methanol-*d*$_4$**	–1.3	–1.5	0.9
	**DMSO-*d*$_6$**	–0.9	–0.7	0.9
**2**-C_1-Me	**Chloroform-*d***	–<4.9[a]	–4.5	0.9

	**Acetone**	–3.7	–3.7	0.8
	**Acetonitrile-d_3**	–3.2	–3.6	0.8
	**Ethyl acetate**	–4.6	–3.9	0.8
	**Tetrahydrofuran**	–4.6	–4.8	0.8
	**Dichloromethane**	<–4.5[a]	–5.0	0.8
	**Ethanol**	–1.7	–1.6	1.0
	**Methanol-d_4**	–1.5	–1.6	1.0
	**DMSO-d_6**	–1.0	–1.1	1.0
**2**-C_1-CF_3	**Chloroform-*d***	<–4.9[a]	–4.6	0.9
	**Acetone**	–4.4	–4.0	0.9
	**Acetonitrile-d_3**	–4.2	–3.9	0.9
	**Ethyl acetate**	–4.6	–4.2	0.8
	**Tetrahydrofuran**	–4.6	–5.1	0.8
	**Dichloromethane**	<–4.5[a]	–5.1	0.8
	**Ethanol**	–2.0	–1.9	1.0
	**Methanol-d_4**	–1.7	–1.9	1.0
	**DMSO-d_6**	–1.3	–1.6	1.0
**2**-C_1-^tBu	**Chloroform-*d***	<–4.9[a]	–4.5	1.3
	**Acetone**	–3.2	–3.2	1.2
	**Acetonitrile-d_3**	–2.5	–3.3	1.3
	**Ethyl acetate**	–4.6	–3.5	1.2
	**Tetrahydrofuran**	–3.6	–4.0	1.2
	**Dichloromethane**	<–4.5[a]	–4.8	1.2
	**Ethanol**	–2.0	–1.9	1.5
	**Methanol-d_4**	–1.5	–1.9	1.5
	**DMSO-d_6**	–1.2	–1.0	1.5
**2**-C_1-Ph	**Chloroform-*d***	<–4.9[a]	–4.6	1.1
	**Acetone**	–3.2	–3.3	1.1
	**Acetonitrile-d_3**	–3.2	–3.3	1.1
	**Ethyl acetate**	–4.6	–3.5	1.1
	**Tetrahydrofuran**	–3.6	–4.2	1.0
	**Dichloromethane**	<–4.5[a]	–4.9	1.1
	**Ethanol**	–1.7	–1.7	1.3
	**Methanol-d_4**	–1.3	–1.7	1.3
	**DMSO-d_6**	–1.0	–0.9	1.4
**2**-C_1-PhOMe	**Chloroform-*d***	<–4.9[a]	–4.5	1.2
	**Acetone**	–3.2	–3.3	1.1
	**Acetonitrile-d_3**	–3.2	–3.3	1.1
	**Ethyl acetate**	–4.6	–3.5	1.1
	**Tetrahydrofuran**	–3.6	–4.2	1.0
	**Dichloromethane**	<–4.5[a]	–4.9	1.1

	**Ethanol**	–1.5	–1.6	1.4
	**Methanol-*d*$_4$**	–1.3	–1.6	1.4
	**DMSO-*d*$_6$**	–1.0	–0.8	1.4
**2**-C$_1$-Pyr	**Chloroform-*d***	–0.9	–0.4	0.7
	**Acetone**	–0.3	–0.3	0.7
	**Acetonitrile-*d*$_3$**	–0.6	–0.3	0.7
	**Ethyl acetate**	–0.2	–0.3	0.6
	**Tetrahydrofuran**	–0.2	–0.3	0.6
	**Dichloromethane**	0.0	–0.4	0.6
	**Ethanol**	–0.3	–0.3	0.8
	**Methanol-*d*$_4$**	0.1	–0.3	0.8
	**DMSO-*d*$_6$**	–0.3	–0.2	0.8
**2**-C$_1$-PyrF	**Chloroform-*d***	–1.5	–1.0	0.7
	**Acetone**	–0.6	–0.6	0.7
	**Acetonitrile-*d*$_3$**	–1.1	–0.7	0.7
	**Ethyl acetate**	–0.7	–0.6	0.7
	**Tetrahydrofuran**	–0.4	–0.6	0.7
	**Dichloromethane**	–0.5	–1.0	0.7
	**Ethanol**	–0.6	–0.6	0.9
	**Methanol-*d*$_4$**	–0.3	–0.6	0.9
	**DMSO-*d*$_6$**	–0.5	–0.3	0.9
**1**-C$_2$-H	**Chloroform-*d***	–2.9	–2.8	0.4
	**Acetone**	–1.9	–1.8	0.4
	**Acetonitrile-*d*$_3$**	–1.7	–1.9	0.4
	**Ethyl acetate**	–2.2	–1.9	0.4
	**Tetrahydrofuran**	–1.9	–2.1	0.4
	**Dichloromethane**	–3.0	–2.9	0.4
	**Ethanol**	–1.0	–1.2	0.5
	**Methanol-*d*$_4$**	–1.0	–1.2	0.5
	**DMSO-*d*$_6$**	–0.7	–0.5	0.5
**1**-C$_2$-Ph	**Chloroform-*d***	–3.5	–3.0	0.7
	**Acetone**	–1.9	–1.9	0.7
	**Acetonitrile-*d*$_3$**	–1.7	–2.0	0.7
	**Ethyl acetate**	–2.6	–2.1	0.7
	**Tetrahydrofuran**	–2.3	–2.5	0.6
	**Dichloromethane**	–3.0	–3.2	0.7
	**Ethanol**	–0.9	–1.0	0.8
	**Methanol-*d*$_4$**	–0.8	–1.0	0.8
	**DMSO-*d*$_6$**	–0.6	–0.3	0.9
**1**-C$_2$-PhOMe	**Chloroform-*d***	–0.6	–0.6	0.3
	**Acetone**	–0.9	–0.7	0.3

	**Acetonitrile-*d*$_3$**	–0.8	–0.6	0.3
	**Ethyl acetate**	–0.7	–0.7	0.3
	**Tetrahydrofuran**	–0.6	–0.8	0.3
	**Dichloromethane**	–0.7	–0.7	0.3
	**Ethanol**	–0.5	–0.5	0.4
	**Methanol-*d*$_4$**	–0.3	–0.5	0.4
	**DMSO-*d*$_6$**	–0.4	–0.5	0.4
**1**-C$_2$-PhCN	**Chloroform-*d***	–1.5	–1.6	0.4
	**Acetone**	–1.1	–0.8	0.4
	**Acetonitrile-*d*$_3$**	–0.9	–0.9	0.4
	**Ethyl acetate**	n.d.[b]	–0.9	0.4
	**Tetrahydrofuran**	–0.6	–0.8	0.4
	**Dichloromethane**	–1.7	–1.5	0.4
	**Ethanol**	–0.9	–0.8	0.5
	**Methanol-*d*$_4$**	–0.7	–0.8	0.5
	**DMSO-*d*$_6$**	–0.4	–0.3	0.5
**2**-C$_2$-Me	**Chloroform-*d***	<–5.5[a]	–5.3	1.5
	**Acetone**	–2.4	–3.1	1.4
	**Acetonitrile-*d*$_3$**	–2.1	–3.2	1.4
	**Ethyl acetate**	–4.3	–3.4	1.4
	**Tetrahydrofuran**	–4.0	–3.9	1.3
	**Dichloromethane**	<–6.0[a]	–5.6	1.4
	**Ethanol**	–1.4	–1.5	1.7
	**Methanol-*d*$_4$**	–1.3	–1.5	1.7
	**DMSO-*d*$_6$**	–0.7	0.0	1.8
**2**-C$_2$-CF$_3$	**Chloroform-*d***	<–5.5[a]	–5.2	1.8
	**Acetone**	–1.5	–1.7	1.7
	**Acetonitrile-*d*$_3$**	–1.5	–2.1	1.7
	**Ethyl acetate**	–1.9	–2.1	1.7
	**Tetrahydrofuran**	–1.6	–1.9	1.6
	**Dichloromethane**	<–6.0[a]	–5.1	1.7
	**Ethanol**	–0.9	–1.5	2.1
	**Methanol-*d*$_4$**	–0.7	–1.5	2.1
	**DMSO-*d*$_6$**	–0.6	0.9	2.1
**2**-C$_2$-^{t}Bu	**Chloroform-*d***	–2.9	–2.8	0.7
	**Acetone**	–0.9	–1.3	0.7
	**Acetonitrile-*d*$_3$**	–0.9	–1.4	0.7
	**Ethyl acetate**	–1.9	–1.5	0.7
	**Tetrahydrofuran**	–1.6	–1.5	0.6
	**Dichloromethane**	–3.0	–2.8	0.7
	**Ethanol**	–0.9	–0.9	0.8

	**Methanol-d_4**	–0.8	–0.9	0.8
	**DMSO-d_6**	–0.3	0.1	0.9
**2**-C_2-Ph	**Chloroform-*d***	–3.5	–3.2	0.8
	**Acetone**	–1.1	–1.3	0.7
	**Acetonitrile-d_3**	–1.4	–1.5	0.7
	**Ethyl acetate**	–1.6	–1.5	0.7
	**Tetrahydrofuran**	–1.3	–1.5	0.6
	**Dichloromethane**	–3.4	–3.2	0.7
	**Ethanol**	–0.6	–1.0	0.9
	**Methanol-d_4**	–0.7	–1.0	0.9
	**DMSO-d_6**	–0.4	0.3	0.8
**2**-C_2-PhOMe	**Chloroform-*d***	–3.5	–3.0	0.9
	**Acetone**	–0.6	–1.2	0.9
	**Acetonitrile-d_3**	–1.0	–1.4	0.9
	**Ethyl acetate**	–1.6	–1.4	0.9
	**Tetrahydrofuran**	–1.6	–1.4	0.8
	**Dichloromethane**	–3.0	–3.0	0.8
	**Ethanol**	–0.6	–0.9	1.1
	**Methanol-d_4**	–0.7	–0.9	1.1
	**DMSO-d_6**	–0.3	0.4	1.1
**2**-C_2-Pyr	**Chloroform-*d***	–0.4	–0.5	0.2
	**Acetone**	–0.4	–0.2	0.2
	**Acetonitrile-d_3**	–0.4	–0.3	0.2
	**Ethyl acetate**	n.d.[b]	–0.3	0.2
	**Tetrahydrofuran**	0.0	–0.1	0.2
	**Dichloromethane**	–0.4	–0.4	0.2
	**Ethanol**	–0.6	–0.5	0.3
	**Methanol-d_4**	–0.5	–0.5	0.3
	**DMSO-d_6**	–0.3	–0.4	0.3
**2**-C_2-PyrF	**Chloroform-*d***	–0.8	–0.8	0.2
	**Acetone**	–0.5	–0.5	0.2
	**Acetonitrile-d_3**	–0.5	–0.6	0.2
	**Ethyl acetate**	–0.7	–0.6	0.2
	**Tetrahydrofuran**	–0.6	–0.5	0.2
	**Dichloromethane**	–0.7	–0.8	0.2
	**Ethanol**	–0.7	–0.6	0.2
	**Methanol-d_4**	–0.7	–0.6	0.2
	**DMSO-d_6**	–0.3	–0.4	0.2

[a]Only one peak observed in NMR spectrum that was assigned to the folded conformer. [b]Not determined due to peak overlap in the NMR spectrum.

S4. Computational methods and data

S4.1 Geometry minimisation and calculated conformational free energies

Geometry minimization and calculated conformational energies

The computationally optimized energies of the folded and unfolded conformers were determined by carrying out conformer distribution searches in the gas phase using Spartan ’14 and DFT/B3LYP/6-31G* to obtain the minimized geometries of the unfolded and folded conformers (Figures S47-S50). The resulting gas phase energies and corresponding energy differences, ΔE_{DFT} in each conformer are reported in Tables S37 and Table S38. Good agreement was found between the computed minimized geometry of the folded conformation of balance **1**-C_1-Me and the X-ray crystal structure of the same compound (Figure S51).

Electrostatic potential calculations

The phenyl fragments of balance including the aniline-X, or amide-Y substituents (as shown in Figure 3C of the main text) were drawn in Spartan ’14 and equilibrium geometry calculations were performed using DFT/B3LYP/6-31G*. Electrostatic potential surfaces were calculated at the 0.002 electrons/Å^3 isosurface and the electrostatic potential taken directly over the axis of each N-H bond donor (ESP_{N-H}, Table S37).

Table S37 Calculated energies of methylene molecular balance series (**1**-C_1-X and **2**-C_1-Y) determined from computationally minimized structures (DFT/B3LYP/6-31G*).

Compound	Energy folded conformer / kJ mol^{-1}	Energy unfolded conformer / kJ mol^{-1}	ΔE_{DFT} / kJ mol^{-1}	ESP_{N-H} /kJ mol^{-1}
**1**-C_1-H	−2168382.72	−2168357.04	−25.68	175.5
**1**-C_1-Ph	−2775006.32	−2774975.31	−31.01	198.1
**1**-C_1-PhOMe	−3075676.88	−3075665.46	−11.42	126.4
**1**-C_1-PhCN	−3017187.93	−3017170.65	−17.28	138.8
**2**-C_1-Me	−2569202.53	−2569168.79	−33.74	228.9
**2**-C_1-CF_3	−3350833.51	−3350803.99	−29.52	227.4
**2**-C_1-^{t}Bu	−2878852.66	−2878820.25	−32.41	185.5
**2**-C_1-Ph	−3072608.36	−3072578.70	−29.66	215.5
**2**-C_1-PhOMe	−3373291.82	−3373263.77	−28.05	205.4
**2**-C_1-Pyr	−3114720.65	−3114712.84	−7.81	53.7
**2**-C_1-PyrF	−3375251.93	−3375239.88	−12.05	74.3

Table S38 Calculated energies of ethylene molecular balance series (**1**-C_2-X and **2**-C_2-Y) determined from computationally minimized structures (DFT/B3LYP/6-31G*).

Compound	Energy folded conformer / kJ mol^{-1}	Energy unfolded conformer / kJ mol^{-1}	ΔE_{DFT} / kJ mol^{-1}	ESP_{N-H} / kJ mol^{-1}
**1**-C_2-H	−2271602.61	−2271581.46	−21.15	175.5
**1**-C_2-Ph	−2878225.95	−2878200.78	−25.17	198.1
**1**-C_2-PhOMe	−3178896.34	−3178882.10	−14.24	126.4
**1**-C_2-PhCN	−3120409.74	−3120395.91	−13.83	138.8
**2**-C_2-Me	−2672423.69	−2672391.95	−31.74	228.9
**2**-C_2-CF_3	−3454051.86	−3454031.22	−20.64	227.4
**2**-C_2-^{t}Bu	−2982069.38	−2982042.84	−26.54	185.5
**2**-C_2-Ph	−3175823.57	−3175801.70	−21.87	215.5
**2**-C_2-PhOMe	−3476505.30	−3476489.23	−16.07	205.4
**2**-C_2-Pyr	−3217944.33	−3217934.66	−9.67	53.7
**2**-C_2-PyrF	−3478472.28	−3478465.21	−7.07	74.3

Optimized geometries of molecular balances

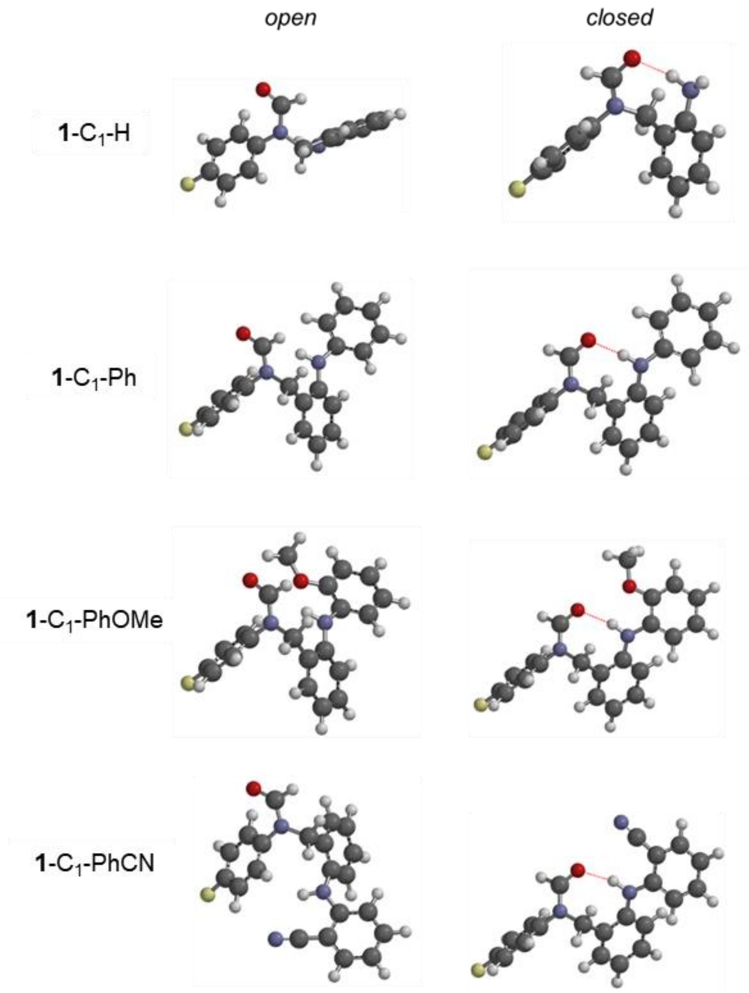

Figure S47 DFT/B3LYP/6-31G* minimized structures of balance series **1**-C_1-X in both the open (unfolded) and closed (folded) conformers.

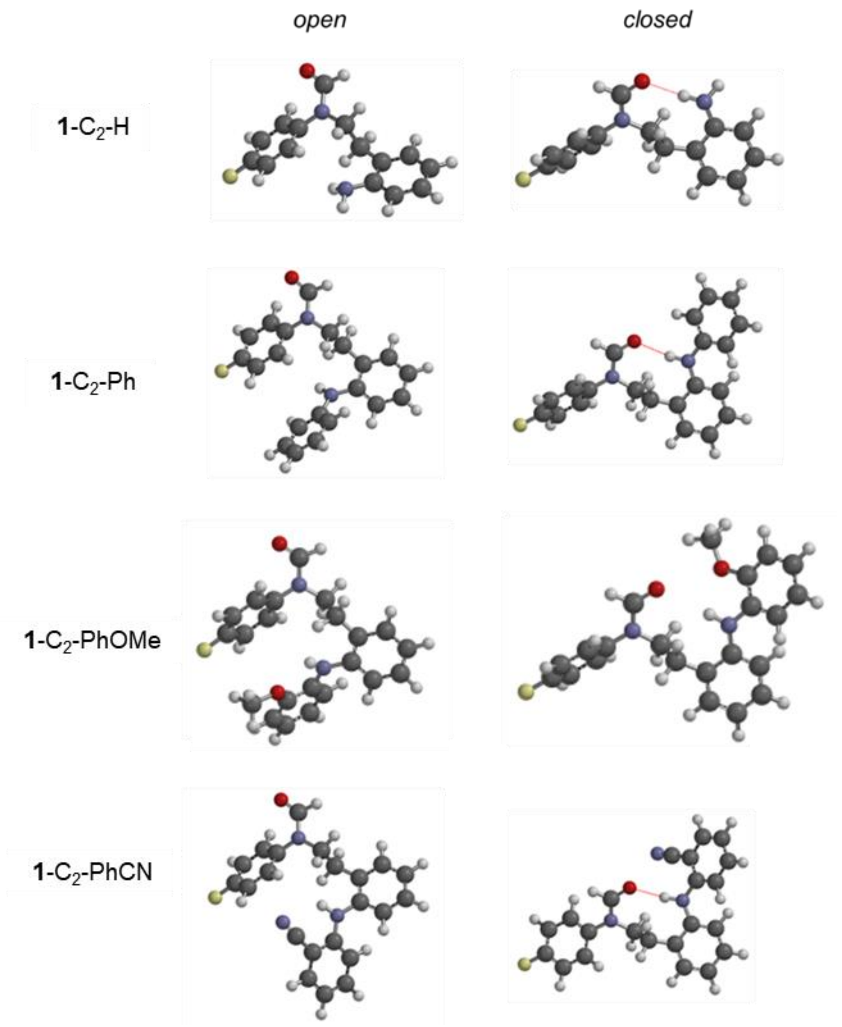

Figure S48 DFT/B3LYP/6-31G* minimized structures of balance series **1**-C_2-X in both the open (unfolded) and closed (folded) conformers.

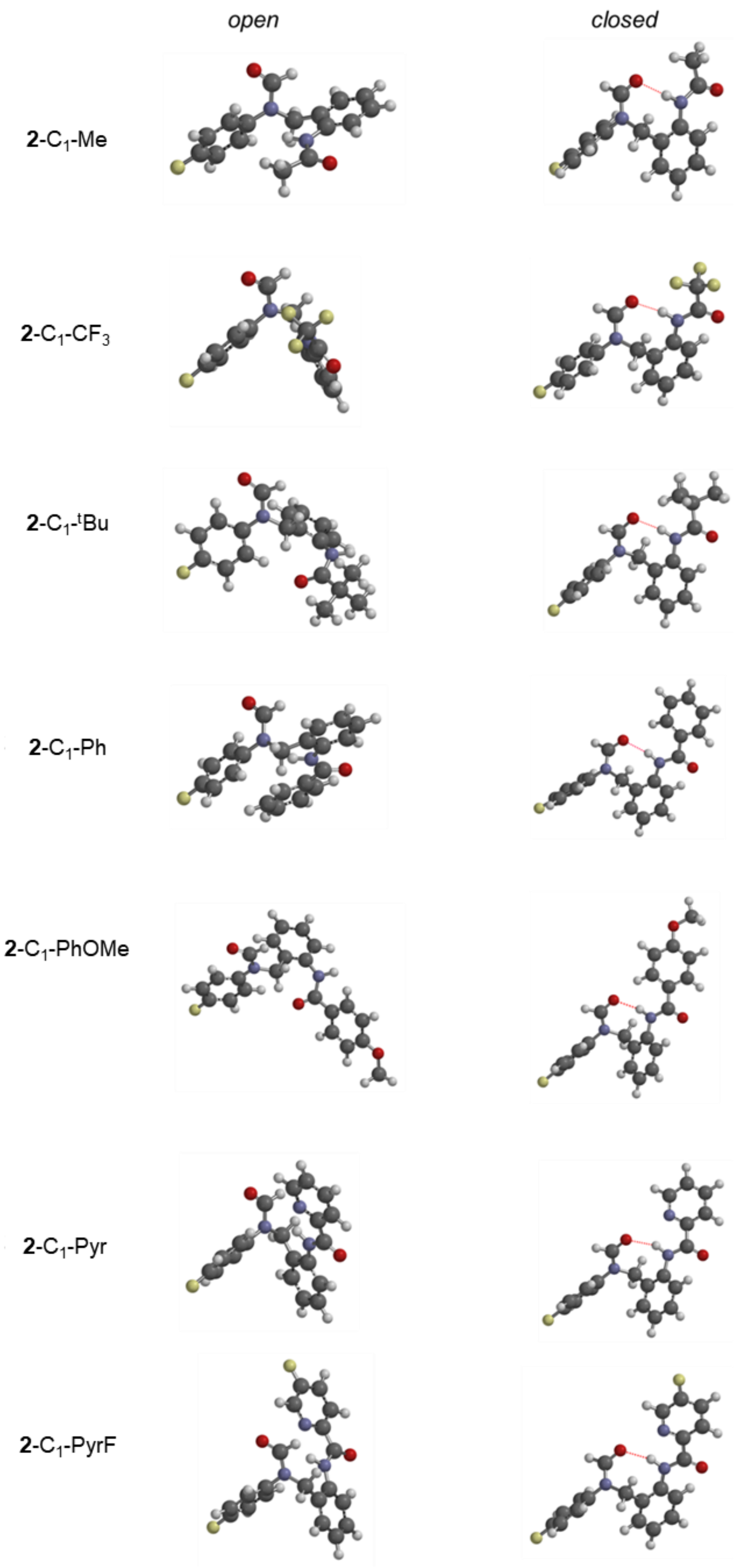

Figure S49 DFT/B3LYP/6-31G* minimized structures of balance series **2**-C_1-Y in both the open (unfolded) and closed (folded) conformers.

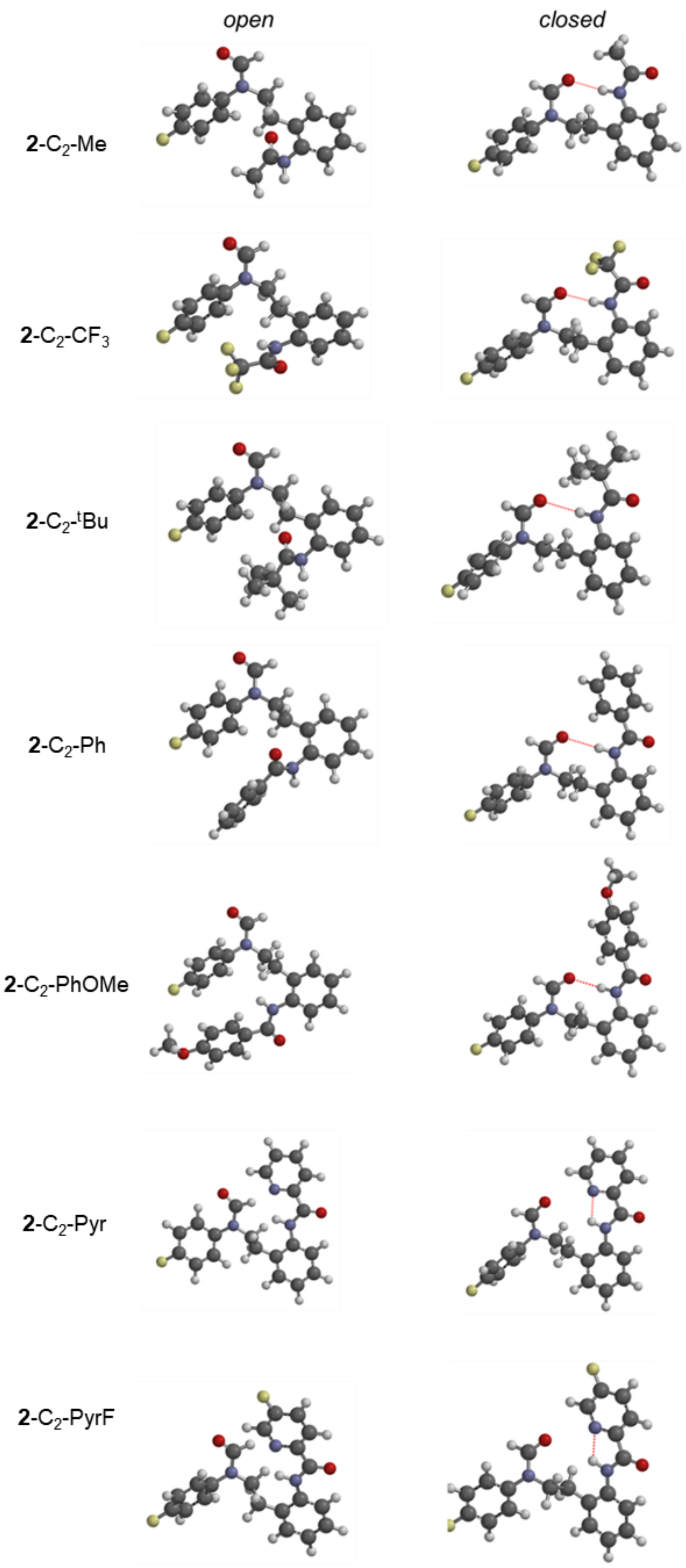

Figure S50 DFT/B3LYP/6-31G* minimized structures of balance series **2**-C_2-Y in both the open (unfolded) and closed (folded) conformers.

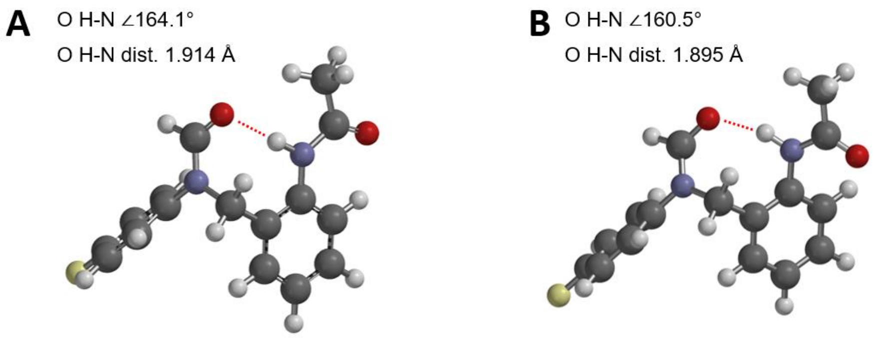

Figure S51 (**A**) Calculated structure of folded conformer of molecular balance **2**-C_1-Me (DFT/B3LYP/6-31G*). (**B**) Obtained crystal structure of **2**-C_1-Me.

S4.2 Barrier to rotation calculation

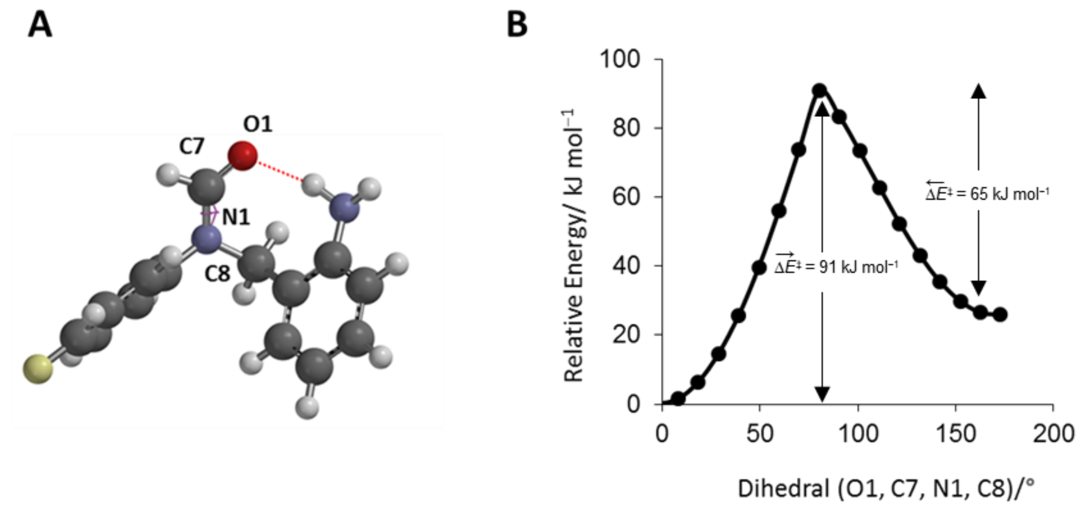

Figure S52 Determination of the barrier to rotation by performing an energy profile calculation (DFT/B3LYP/6-31G*) for molecular balance **1**-C_1-H (see Table S39).

Table S39 Energy profile calculation performed for molecular balance **1**-C_1-H (DFT/B3LYP/6-31G*).

Relative energy / kJ mol^{-1}	Dihedral / °
0.0	−2.0
1.5	8.3
6.5	18.6
14.6	28.9
25.7	39.2
39.8	49.5
56.1	59.8
73.7	70.1
90.8	80.3
105.8	90.6
116.9	100.9
123.4	111.2
67.1	121.5
57.7	131.8
49.5	142.1
43.0	152.3
39.0	162.6
36.3	172.9
25.9	−176.8
26.6	−166.5
29.9	−156.2
35.5	−146.0
43.1	−135.7
52.4	−125.4
62.8	−115.1
73.4	−104.8
83.5	−94.5
92.0	−84.2
98.0	−74.0
99.6	−63.7
57.0	−53.4
43.9	−43.1
33.1	−32.8
25.3	−22.5
20.1	−12.2
18.3	−2.0

S3.2 Experimental energies vs. calculated properties

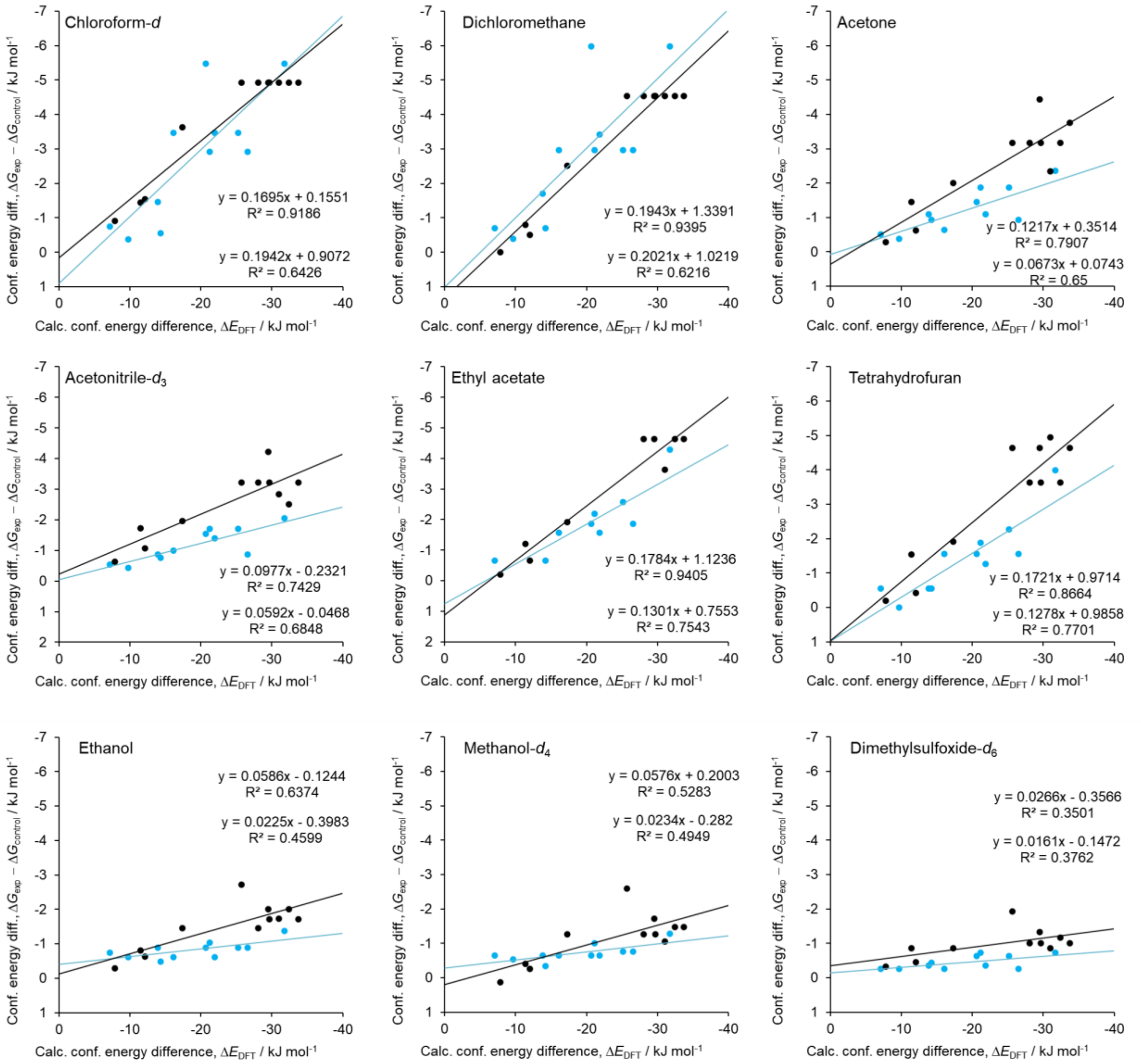

Figure S53 Correlations of ΔG_{exp} measured in different solvents vs. the calculated conformational energy difference calculated using DFT (B3LYP/6-31G*). Black points correspond to the methylene-linker series, and blue to the ethylene-linked series.

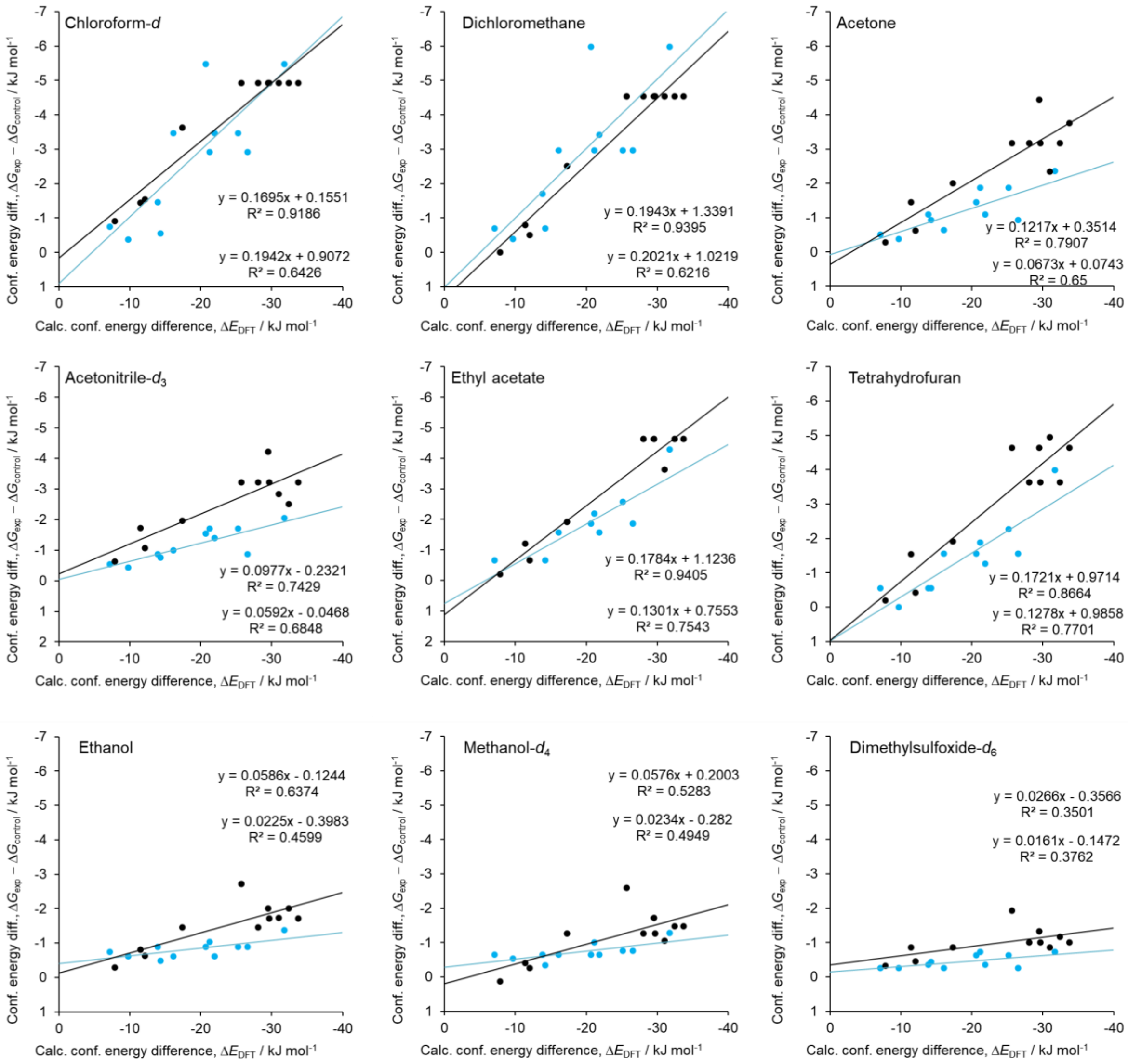

Figure S54 Correlation of $\Delta G_{exp} - \Delta G_{exp}$ vs. the calculated electrostatic potential of the H-bond donor N-H (B3LYP/6-31G*) from the minimized fragments shown in Figure 3C of the main text. Black points correspond to the methylene-linker series, and blue to the ethylene-linked series.

S5. References

[S1] I. K. Mati, C. Adam, S. L. Cockroft, *Chem. Sci.* **2013**, *4*, 3965–3972.

[S2] B. W. Gung, B. U. Emenike, M. Lewis, K. Kirschbaum, *Chem. Eur. J.* **2010**, *16*, 12357–12362.

[S3] M. Bauer, A. Bertario, G. Boccardi, X. Fontaine, R. Rao, D. Verrier, *J. Pharm. Biomed. Anal.* **1998**, *17*, 419–425.

[S4] a) C. A. Hunter, *Angew. Chem. Int. Ed.* **2004**, *43*, 5310–5324; *Angew. Chem.* **2004**, *116*, 5424–5439; b) R. Cabot, C. A. Hunter, L. M. Varley, *Org. Biomol. Chem.* **2010**, *8*, 1455–1462.

[S5] O. V. Dolomanov, L. J. Bourhis, R. J. Gildea, J. A. K. Howard, H. Puschmann, *J. Appl. Cryst.*, **2009**, *42*, 339–341.

[S6] L. J. Bourhis, O. V. Dolomanov, R. J. Gildea, J. A. K. Howard, H. Puschmann, *Acta Cryst.* **2015**, *A71*, 59–75.

[S7] G. M. Sheldrick, *Acta Cryst.* **2008**, *A64*,112–122.

[S8] F. Kleemiss, O. V. Dolomanov, M. Bodensteiner, N. Peyerimhoff, L. Midgley, L. J. Bourhis, A. Genoni, L. A. Malaspina, D. Jayatilaka, J. L. Spencer, F. White, B. Grundkötter-Stock, S. Steinhauer, D. Lentz, H. Puschmann, S. Grabowsky, *Chem. Sci.* **2021**,*12*, 1675–1692.

[S9] F. Neese, *WIREs Comput. Mol. Sci.*, **2012**, *2*, 73–78.

[S10] L. Yang, C. Adam, S. L. Cockroft, *J. Am. Chem. Soc.* **2015**, *137*, 10084–10087.
